# Experimentally Validated Ab Initio Crystal Structure Prediction of Novel Metal–Organic Framework Materials

**DOI:** 10.1021/jacs.2c12095

**Published:** 2023-01-31

**Authors:** Yizhi Xu, Joseph M. Marrett, Hatem M. Titi, James P. Darby, Andrew J. Morris, Tomislav Friščić, Mihails Arhangelskis

**Affiliations:** † Faculty of Chemistry, University of Warsaw; 1 Pasteura Street, Warsaw 02-093, Poland; ‡ Department of Chemistry, McGill University; 801 Sherbrooke Street West, Montréal, Québec H3A 0B8, Canada; § Department of Engineering, University of Cambridge; Trumpington Street, Cambridge CB2 1PZ, UK; ∥ School of Metallurgy and Materials, University of Birmingham; Edgbaston, Birmingham B15 2TT, UK; ⊥ School of Chemistry, University of Birmingham; Edgbaston, Birmingham B15 2TT, UK

## Abstract

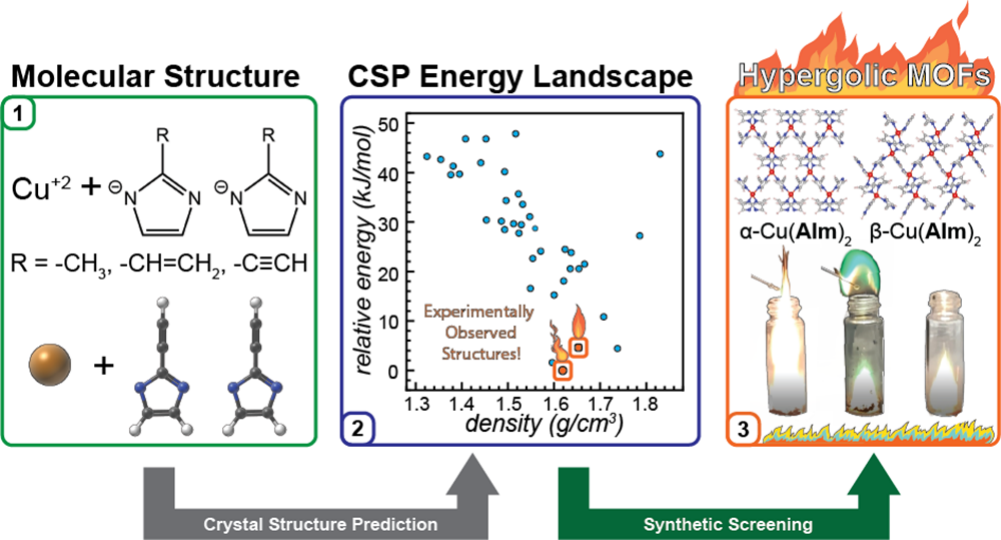

First-principles crystal structure prediction (CSP) is the most powerful approach for materials discovery, enabling the prediction and evaluation of properties of new solid phases based only on a diagram of their underlying components. Here, we present the first CSP-based discovery of metal–organic frameworks (MOFs), offering a broader alternative to conventional techniques, which rely on geometry, intuition, and experimental screening. Phase landscapes were calculated for three systems involving flexible Cu(II) nodes, which could adopt a potentially limitless number of network topologies and are not amenable to conventional MOF design. The CSP procedure was validated experimentally through the synthesis of materials whose structures perfectly matched those found among the lowest-energy calculated structures and whose relevant properties, such as combustion energies, could immediately be evaluated from CSP-derived structures.

## Introduction

Metal–organic frameworks (MOFs) are a highly popular and diverse class of functional solid materials whose modularity makes them applicable to an increasingly large set of applications. To date, MOFs have been developed for purposes including gas sorption^1^ and separation,^2^ sensing,^3^ drug delivery,^4^ aerospace propulsion,^5,6^ catalysis,^7,8^ and more.^9^ The design of MOFs is based on considerations of geometry and chemical intuition, embodied in well-established isoreticular^10^ or node-and-linker approaches, in which the coordination-driven assembly of pre-designed building blocks leads to the formation of networks with predictable connectivities. Such design approaches, however, are limited to components with very rigid structures and predictable binding geometries. Even so, the inherent flexibility of coordination bonds can give rise to new structures and polymorphs that cannot be predicted through node-and-linker considerations alone.^11^

The described challenge in MOF design is exacerbated for materials classes such as zeolitic imidazolate frameworks (ZIFs), where the zeolite-like connectivity of building blocks can give rise to a myriad of possible network topologies whose formation is dictated by a variety of factors, including not only the choice of node and linker but also the selection and distribution of linker substituents, synthetic methodology, and more. Given the central importance that the structure holds for the function of a MOF, it is expected that a general, reliable, and universal first-principles (ab initio) approach for the crystal structure prediction (CSP) of such materials should provide a major advance needed to make the next step in their design and development. While recent extensive studies using periodic density functional theory (DFT) calculations have attempted to address the issue of predicting the structures and polymorphism of MOFs, they have been confined to experimental structures found in the Cambridge Structural Database (CSD)^12^ or their topological equivalents obtained by metal and/or ligand replacements.^13−16^

The scope of theoretical MOF design based on experimental databases as a source of structural candidates remains limited, however, as it confines the potential for materials discovery and development to the already known topological space, largely eliminating the opportunities to discover materials based on previously unreported topologies. The scale of the opportunities to be found by applying CSP to MOFs can be envisaged by considering the progress that the method has brought to the other areas of materials chemistry, such as battery electrode materials,^17,18^ semiconductors, and phase transformations occurring under extreme conditions.^19,20^ In pharmaceutical materials science, CSP is now an invaluable tool to evaluate potential for polymorphism^21^ and for systematic design of drug solid forms.^22^ CSP has also become a powerful tool in designing porous molecular materials^23^ and organic semiconductors.^24^

The development of fully ab initio methods to predict MOF structures has been hindered by their extended hybrid organic–inorganic makeup. Whereas force-field methods are often used for preliminary energy minimization in the CSP of molecular solids in which the putative crystal structures differ only in terms of non-covalent intermolecular interactions, CSP for MOFs requires an accurate description of covalent bond formation between metal nodes and organic linkers. This consideration necessitates the use of periodic DFT methods for energy minimization. Because the unit cell dimensions of MOFs are often significantly larger than those of inorganic systems, the computational cost of such an approach is very high. In order to best utilize the often-encountered high symmetry of MOF structures and reduce the cost of the calculations, we have previously developed an approach that combines the ab initio random structure search (AIRSS) method^25^ with the Wyckoff Alignment of Molecules (WAM) procedure for assigning space group symmetry to putative structures.^26^ The WAM procedure relates the point group symmetry of MOF building blocks to the space group symmetry of the randomly generated structures, thus greatly improving the efficiency of the structure search and reducing its computational cost. The introduction of WAM is key for successful CSP of MOFs, which was recently verified^26^ through computational generation of structures matching the archetypes of several important MOF families, including hexafluorosilicate (SIFSIX), carboxylate (MOF-74 or CPO-27) and metal azolate frameworks (MAFs). That initial report of CSP for MOFs, however, has focused on already known materials, all based on zinc(II) nodes with filled *d*-orbital shells, and has not tackled the challenge of predicting and/or experimentally validating the structures of not yet reported systems.

Here, we present the first experimentally-validated ab initio CSP and discovery of previously not reported MOF compositions, based on Cu^2+^ ions as nodes. Due to their *d*^9^ electronic configuration, the Cu^2+^ nodes are prone to significant distortions from idealized coordination geometries; therefore, their use may lead to unusual framework geometries and network topologies. Specifically, we demonstrate the successful CSP for three examples of rare copper(II)-based ZIFs selected for their potential to exhibit hypergolic behavior, which is a key property in the development of space propulsion technologies and was only recently observed in MOFs.^5,27^ The ab initio predictions were validated through subsequent synthesis and structural characterization that, in each case, revealed a computationally predicted low-energy crystal structure for each composition.

Based on divalent metal nodes and imidazolate organic linkers, whose binding geometries mimic those found in zeolites and silicates, ZIFs are one of the most widely studied classes of MOFs.^28^ The most common metal nodes in ZIF design are zinc,^29^ cobalt,^30^ and cadmium,^31^ with a surprising absence of other potentially tetrahedral elements such as copper. So far, the only structurally characterized copper(II) systems appear to be based on the unsubstituted imidazole (H**Im**) as the linker.^31,32^ Our specific interest in ZIFs lies in the observation that using linkers substituted with unsaturated vinyl or acetylene moieties yield materials exhibiting hypergolic behavior, i.e., spontaneous and rapid ignition in contact with an oxidizer. Moreover, hypergolicity—a necessary property for developing new satellite and spacecraft propulsion systems—appears to be facilitated by the use of redox-active metal ions as ZIF nodes^5^ and in ionic liquid compositions.^33,34^

The outstanding rarity of copper(II)-based materials among ZIFs, combined with the ability of such systems to exhibit hypergolic behavior of potential value in the design of hypergolic propellants,^35−37^ inspired us to focus on copper(II)-based ZIFs as suitably novel and challenging targets for this first proof-of-principle application of CSP for MOF discovery.

## Results and Discussion

We first targeted the prediction of the structural landscape for a ZIF composed of Cu^2+^ nodes and linkers generated from an acetylene-substituted imidazole (H**AIm**). As our previous work^5^ has shown that ZIFs containing **AIm**^–^ linkers display rapid ignition and intense combustion on contact with an oxidizer, the target Cu(**AIm**)_2_ was of particular interest in terms of developing new hypergolic materials. Trial Cu(**AIm**)_2_ crystal structures were generated by randomly placing Cu atoms and isolated **AIm** linkers in a 1:2 stoichiometric ratio within a unit cell of arbitrary dimensions using the AIRSS and WAM algorithms. Thousands of such structures were generated, each containing one, two, three, or four ZIF formula units per primitive unit cell (see the SI for details). These input structures were then energy-minimized using the PBE functional^38^ combined with the Grimme D2^39^ dispersion correction in the plane-wave DFT code CASTEP19.^40^ The optimized structures were ranked in the order of increasing energies, and duplicate structures were merged. Subsequently, an energy window of 100 kJ mol^–1^ was chosen, where each unique low energy structure was re-optimized using a more accurate model based on the PBE functional and many-body dispersion (MBD*) scheme.^41−43^ Finally, all predicted structures were classified in terms of metal coordination geometry through the τ_4_ geometry index,^44^ ranging from perfect square planar geometry (τ_4_ = 0) to tetrahedral geometry (τ_4_ = 1).

**Figure 1:**
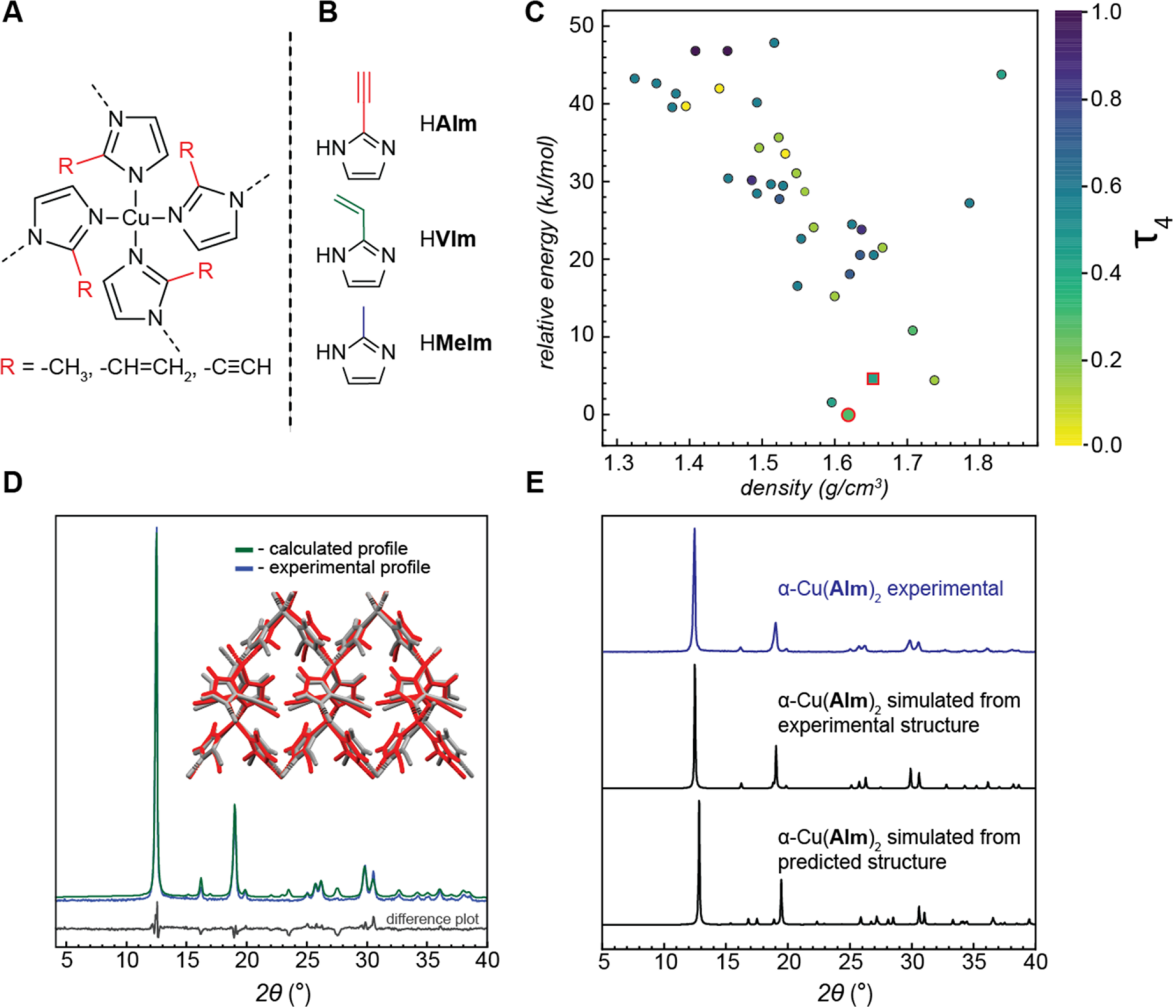
(A) Chemical diagram of copper(II)-based ZIFs. (B) 2-Subsitututed imidazole linkers used in this study. (C) Calculated energy landscape of Cu(**AIm**)_2_. Each dot in the plot represents a unique crystal structure and is colored against its Cu coordination geometry index (τ_4_), with the value of 0 (yellow) being the perfect square planar geometry and purple being the tetrahedral geometry. Structures with two unique copper sites are colored based on the average τ_4_ value. Global energy minimum α-Cu(**AIm**)_2_ is marked by the red-bordered dot, while the β-Cu(**AIm**)_2_ structure generated via perturbation analysis is marked by the red-bordered square. (D) Rietveld refinement of predicted structure for α-Cu(**AIm**)_2_ against experimental powder diffraction data. Inset: predicted (red) crystal structure overlaid against the experimental (gray) structure determined by single-crystal X-ray diffraction. The full match between the structures is evident from the low RMSD value of 0.453 Å. (E) Comparison of experimental powder X-ray diffractogram with diffractograms simulated from the experimental and predicted structures of α-Cu(**AIm**)_2_.

The energy landscape of the final set of unique structures is shown (Figure 1 and Table S2), where the calculated global minimum was found to be a dense structure of *I4*_1_ crystallographic symmetry, adopting a diamondoid (*dia*) topology. It is also noteworthy that the overall crystal energy landscape of Cu(**AIm**)_2_ shows a strong preference toward the formation of high density-structures with no or little (calculated void fraction less than 10%) void space within 20 kJ mol^–1^ (Figure S1 and Table S2). This is in stark contrast to the Zn-based ZIFs, for which our DFT calculations and calorimetric measurements revealed the formation of highly porous polymorphs within 10–20 kJ mol^–1^ of the non-porous most stable polymorphs.^45−47^

An important metric for evaluating the potential performance of a material as a hypergolic fuel is the volumetric energy density (*E*_v_), i.e., ratio of the combustion enthalpy released per unit volume of the material. These quantities were derived from the enthalpies of combustion calculated with periodic DFT (Table 1). The calculated *E*_v_ for *dia*-Cu(**AIm**)_2_ was found to be 33.3 kJ cm^–3^, markedly higher than those of our previously reported hypergolic Zn(**AIm**)_2_, Co(**AIm**)_2_, and Cd(**AIm**)_2_ materials based on a porous sodalite (*SOD*) topology (19.3, 19.7, and 16.3 kJ cm^–3^, respectively) and higher than that of the currently used hydrazine hypergols.^48^ While the high calculated *E*_v_ for the predicted *dia*-Cu(**AIm**)_2_ structure is consistent with the observation of higher values (up to 37 kJ cm^–3^) in other high-density, non-porous ZIFs based on zinc, none of those previously investigated systems exhibited hypergolicity.

**Table 1:** Calculated Combustion Energies for the Predicted Copper MOF Structures

ZIF	Δ*E*_c_ (kJ mol^–1^)	*E*_g_ (kJ g^–1^)	*E*_v_ (kJ cm^–3^)
α-Cu(**AIm**)_2_	–5048.6	20.6	33.3
β-Cu(**AIm**)_2_	–5053.3	20.6	34.0
Cu(**VIm**)_2_	–5093.9	20.4	34.1
Cu(**MeIm**)_2_	–4186.4	18.7	28.4

Consequently, the predicted high *E*_v_ in combination with potential for hypergolic behavior makes *dia*-Cu(**AIm**)_2_ a particularly attractive synthetic target.

To experimentally explore the phase landscape of the Cu(**AIm**)_2_ system and validate the accuracy of the CSP results, we performed a set of solution and mechanochemical experiments (SI and Figures S15, S18, and S21) using the H**AIm** ligand and diverse sources of copper(II). The produced solids were analyzed primarily via powder X-ray diffraction (PXRD). In some cases, a purple microcrystalline powder was obtained, and PXRD analysis indicated a possible structural match with the global minimum Cu(**AIm**)_2_ from CSP. Optimization of the synthetic procedure, based on adding H**AIm** ligand to a solution of copper(II) sulfate in dilute aqueous ammonia, yielded this Cu(**AIm**)_2_ material in a phase-pure form, which was confirmed by a combination of PXRD, thermogravimetric analysis (TGA), and infrared spectroscopy (IR). Further modification of the procedure allowed for the formation of deep-purple single crystals alongside a yet unidentified dark-brown amorphous impurity. Analysis of the crystals by single-crystal X-ray diffraction (SCXRD) revealed a structure that fully matched the global minimum Cu(**AIm**)_2_ (Table S9) phase predicted by our CSP methodology, validating our approach for the prediction of MOF structures. This predicted and experimentally obtained *dia*-Cu(**AIm**)_2_ structure was designated as the α-phase.

During the synthesis and handling of the α-form of Cu(**AIm**)_2_, it became evident that mechanical stress, such as scraping with a spatula or packing into a PXRD sample holder, induced a color change in the material from purple to dark green. Consistent with these observations and the hypothesis that grinding or crushing induces a phase change in the α-phase, impact stability testing of α-Cu(**AIm**)_2_ with a total energy of 50 J (see the SI) produced a dark green material whose PXRD pattern exhibited new Bragg reflections, indicating the formation of an additional crystalline phase. Additionally, a dark green material with a PXRD pattern different from that of the α-Cu(**AIm**)_2_ was obtained during mechanochemical screening reactions (SI Table S15). Complete conversion of the α-form to this new β-form, as evidenced by the disappearance of the original and the emergence of new Bragg reflections in the PXRD pattern of the material, could be achieved by ball milling of the pristine α-Cu(**AIm**)_2_ material for 20 min (Figure S13). The poor quality of the PXRD data and lack of single crystals for the new β-phase made its experimental structure determination challenging, motivating us to pursue structure elucidation by computational means.

The ability of α-Cu(**AIm**)_2_ to undergo rapid transformation to β**-**Cu(**AIm**)_2_ upon mechanical impact indicates that there is likely a degree of crystallographic and topological similarity between the two structures, such that the transformation could occur without breaking the covalent bonds and with only moderate distortion of the unit cell and molecular packing. Based on this assumption, we performed a post hoc systematic distortion analysis of the α-Cu(**AIm**)_2_ structure. The original α-Cu(**AIm**)_2_ tetragonal cell, obtained by CSP, was perturbed in 12 symmetry-independent distortion modes, and each perturbed structure was geometry-optimized in CASTEP (see the SI for details).

**Figure 2:**
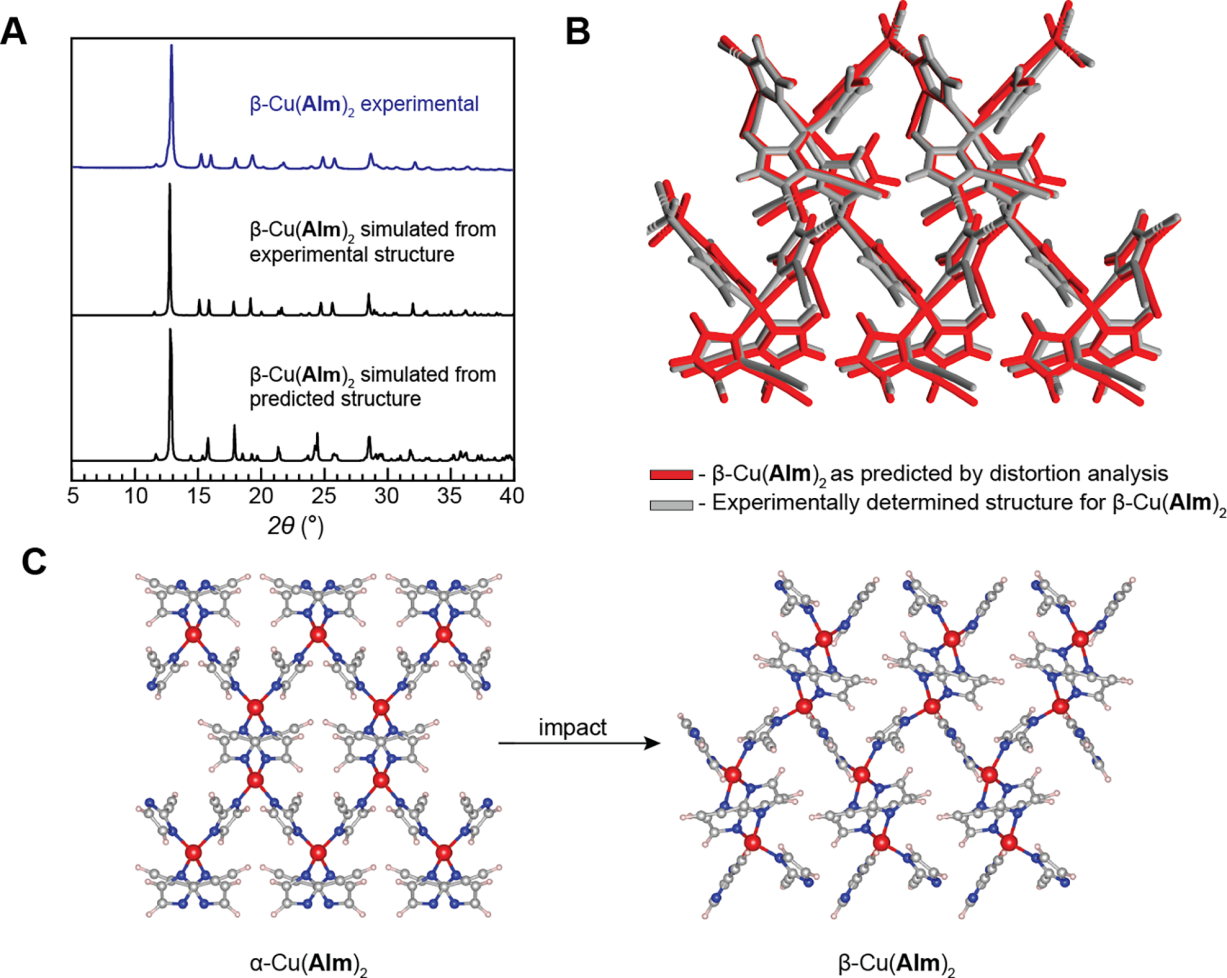
(A) Powder X-ray diffractogram measured for the synthesized β-Cu(**AIm**)_2_ material compared with the diffractograms simulated from the crystal structure refined from powder data and the diffractogram simulated for the structure as generated by unit cell distortion analysis. (B) Theoretical (red) crystal structure overlaid on top of the experimental (gray) structure refined from powder X-ray diffraction data. The full match between the structures is evident from the low RMSD value of 0.501 Å. (C) Schematic of impact-induced conversion of α-Cu(**AIm**)_2_ to β-Cu(**AIm**)_2_.

This perturbation analysis led to a structure with a simulated PXRD pattern closely matching the one experimentally measured for the β-phase (Figure 2), allowing for the Rietveld refinement of the structural model derived from the perturbation analysis. The derived structure was found to be just 4.6 kJ mol^–1^ higher in energy than the original α-Cu(**AIm**)_2_, the small energy difference consistent with the ease of the phase transformation occurring under experimental conditions.

Compared to the α-Cu(**AIm**)_2_, the β-form has a higher density (1.65 g cm^–3^) and is of lower symmetry (space group *P*2_1_). Moreover, the β-form contains four crystallographically unique imidazolate linkers in contrast to the only one in the α**-**phase. There are also two symmetry-independent and geometrically distinct copper(II) nodes, one of which is best described with seesaw geometry (τ_4_ = 0.45), while the other one adopts a nearly square-planar geometry (τ_4_ = 0.2). It is this structural complexity of β-Cu(**AIm**)_2_ that posed a particular challenge for WAM + AIRSS ab initio CSP search normally geared toward highly symmetric MOF structures. The perturbation approach presented herein, capable of producing lower symmetry distortions of the predicted structures, shall become an integral part of our CSP protocol in the future.

**Figure 3:**
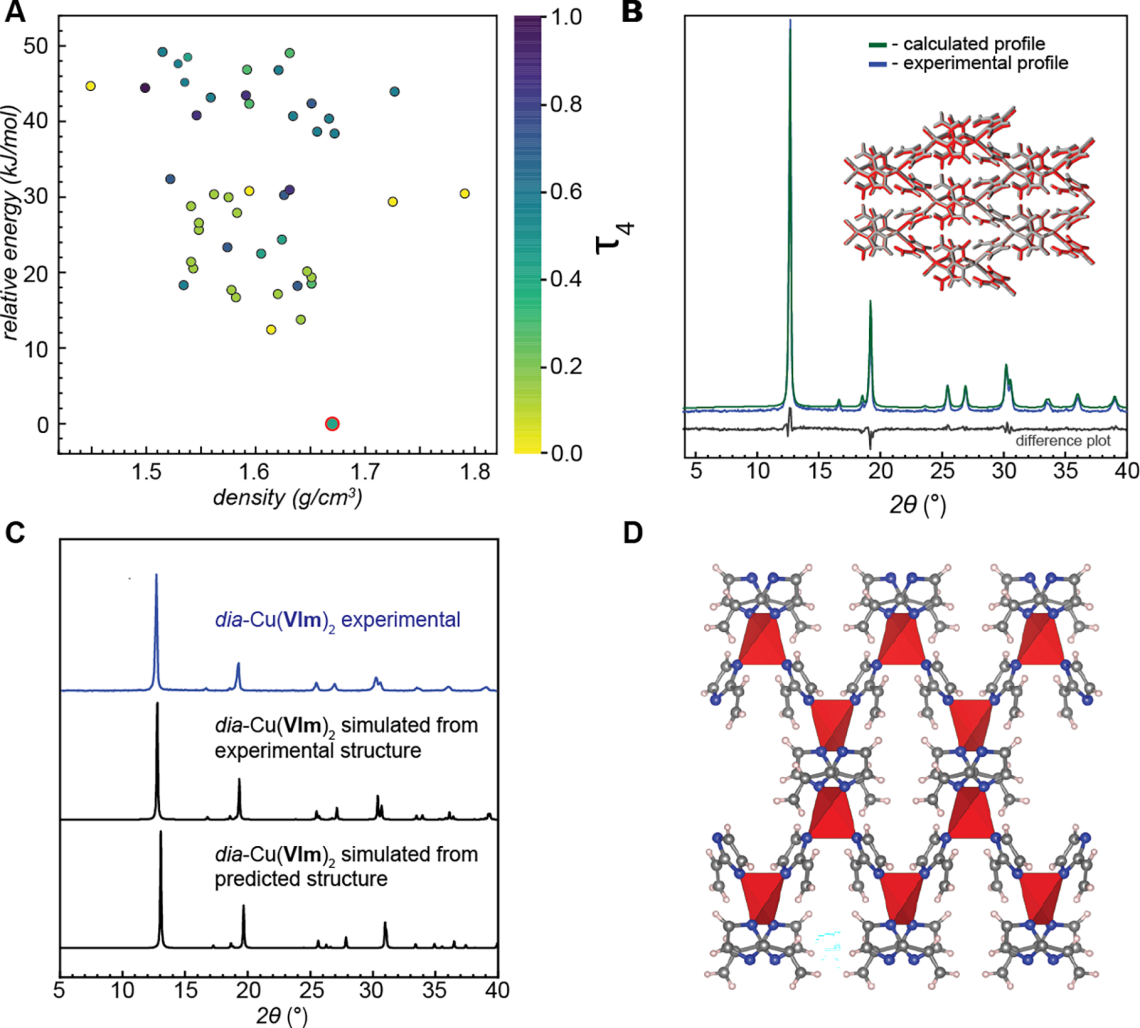
(A) Calculated energy landscape of Cu(**VIm**)_2_. Each dot in the plot represents a unique crystal structure and is colored against its Cu coordination geometry index (τ_4_), with the value of 0 (yellow) being the perfect square planar geometry, and purple being the tetrahedral geometry. Structures with two unique copper sites are colored based on the average τ_4_ value. The lowest energy structure in the plot, matching the experimental structure, is highlighted with a red circle. In addition, two structures generated with the perturbation analysis that could potentially represent the hypothetical β form of Cu(**VIm**)_2_ are shown with red dots. (B) Rietveld refinement of predicted structure for α-Cu(**VIm**)_2_ against experimental powder diffraction data. Inset: predicted (red) crystal structure overlaid on top of the experimental (gray) structure determined by single-crystal X-ray diffraction. The full match between the structures is evident from the low RMSD value of 0.147 Å. (C) Comparison of the experimental and simulated PXRD patterns for the *dia* structure of α-Cu(**VIm**)_2_ (D) Crystal structure diagram of α-Cu(**VIm**)_2_.

Encouraged by the successful prediction of the experimentally observed structure of α-Cu(**AIm**)_2_ and the derivation of β-Cu(**AIm**)_2_ through unit cell distortion analysis, we applied the same CSP methodology to the analogous system generated from the 2-vinylimidazole (H**VIm**) linker (Figure 3). The calculated global energy minimum Cu(**VIm**)_2_ structure was found to be a *dia*-topology framework, isostructural to α**-**Cu(**AIm**)_2_. However, in this case, no other structures were found in the vicinity of the global energy minimum, and the next lowest energy structure was located 12.4 kJ mol^–1^ above it. These observations indicate a strong preference of Cu(**VIm**)_2_ to adopt this particular *dia*-structure. The perturbation analysis of the global minimum predicted structure of Cu(**VIm**)_2_ revealed a hypothetical monoclinic β-phase at 16.8 kJ mol^–1^ above the global minimum (Table S5), far higher in energy than in the case of Cu(**AIm**)_2_. Overall, the ab initio CSP calculations of Cu(**VIm**)_2_ coupled with symmetry perturbation analysis of the global minimum structure suggested the formation of a single polymorph as the most likely outcome of experimental synthesis. Calculation of the enthalpy of combustion for Cu(**VIm**)_2_ revealed an *E*_v_ of 34.1 kJ mol^–1^, which is slightly higher than for the isostructural α**-**Cu(**AIm**)_2_, again presenting the potential to obtain a material that can combine a high *E*_v_ with hypergolicity (Table 1). Also, consistent with the CSP results for Cu(**AIm**)_2,_ the energy landscape of Cu(**VIm**)_2_ contains only dense non-porous structures within 20 kJ mol^–1^ above the global energy minimum.

Armed with the highly promising results from the theoretical structure and energy density predictions, we turned toward the synthesis of Cu(**VIm**)_2_. Like for Cu(**AIm**)_2_, we performed a set of mechanochemical and solution-based synthetic screening experiments (SI and Figures S16, S19, and S22), which in some cases produced microcrystalline powders with PXRD patterns matching the one calculated for the CSP global energy minimum Cu(**VIm**)_2_ structure. Optimization of the synthesis resulted in a procedure based on stirring tetraaminecopper(II) sulfate monohydrate and H**VIm** in a small amount of water, which produced this Cu(**VIm**)_2_ material in phase-pure form as confirmed by PXRD, TGA, and IR spectroscopy. We were also able to isolate Cu(**VIm**)_2_ in the form of diffraction-quality dark-blue single crystals. Crystal structure analysis by X-ray crystallography revealed a structure that fully matched the global energy minimum Cu(**VIm**)_2_ structure generated by our CSP methodology, further validating our approach. During synthetic screenings, two other crystalline phases were observed but were ruled out as Cu(**VIm**)_2_ polymorphs on the basis of either their copper content, as determined by TGA, or their solubility in organic solvent. No other crystalline materials were observed during our experimental screening, including trials to induce polymorph transformations via mechanical impact, the latter being consistent with the results of unit cell distortion analysis.

**Figure 4:**
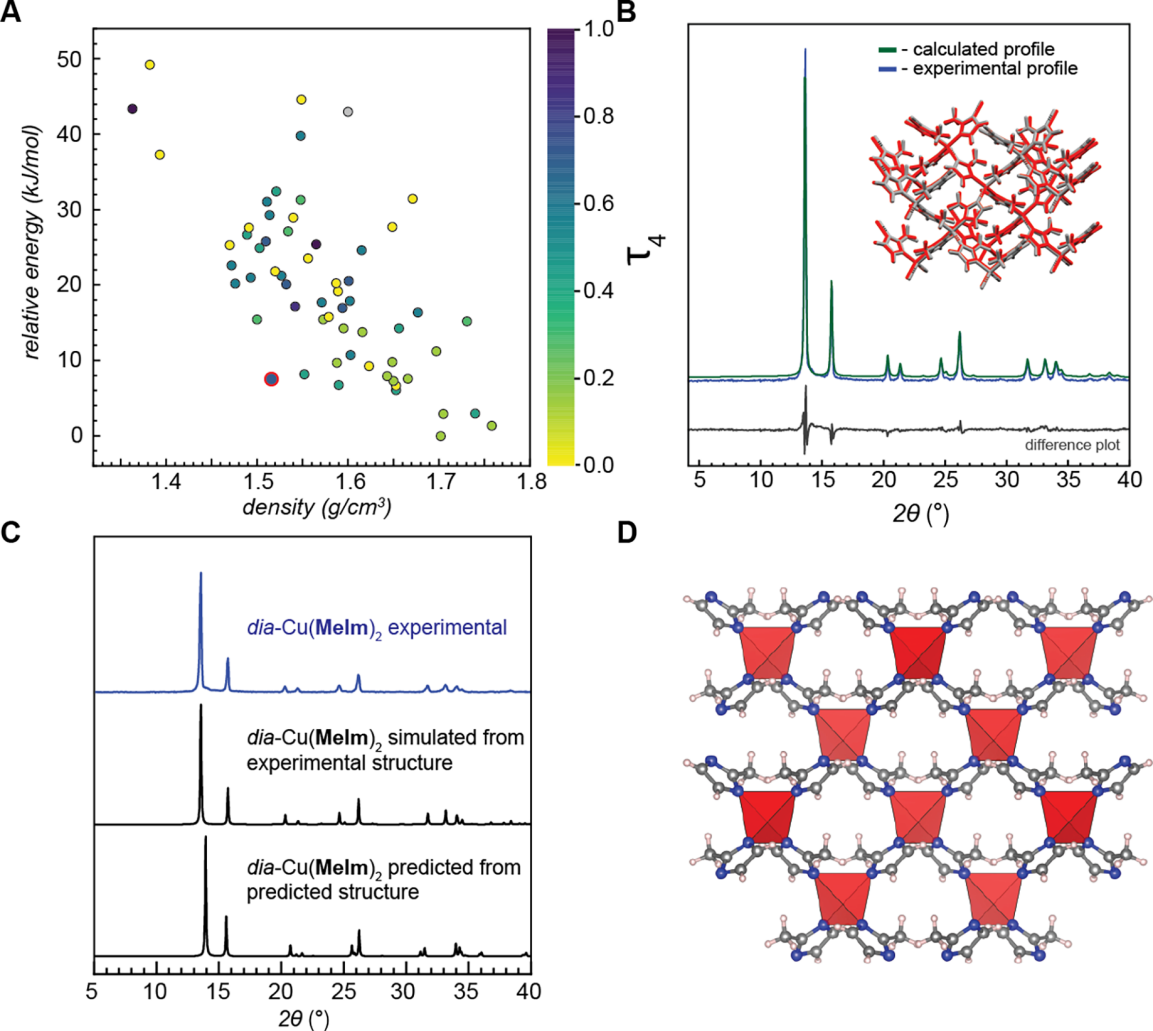
(A) Calculated energy landscape of Cu(**MeIm**)_2_. Each dot in the plot represents a unique crystal structure and is colored against its Cu coordination geometry index (τ_4_), with the value of 0 (yellow) being the perfect square planar geometry and purple being the tetrahedral geometry. Structures with two unique copper sites are colored based on the average τ_4_ value. The experimentally matching structure is highlighted with a red circle. (B) Rietveld refinement of the predicted structure for *dia*-Cu(**MeIm**)_2_ against experimental powder diffraction data. Inset: predicted (red) crystal structure overlaid on top of the experimental (gray) structure determined by single-crystal X-ray diffraction. The full match between the structures is evident from the low RMSD value of 0.211 Å. (C) Comparison of the experimental and simulated PXRD patterns for the *dia* structure of Cu(**MeIm**)_2_. (D) Crystal structure diagram of *dia*-Cu(**MeIm**)_2_.

Finally, we performed CSP for the framework based on the methyl-substituted imidazole ligand, Cu(**MeIm**)_2_ (Figure 4). Unlike the previous two systems, where the global energy minimum was found to be a 3D *dia*-topology framework; in this case, the putative structural landscape revealed a two-dimensional (2D) structure of square lattice (*sql*) topology as the global energy minimum, and the two subsequent lowest energy structures with energies of +1.4 and 2.9 kJ mol^–1^ were above the global minimum. Starting from the fourth lowest energy structure (relative energy, +3.0 kJ mol^–1^), 3D frameworks with *dia*-topology appear. Overall, porosity trends among the 3D-predicted structures of Cu(**MeIm**)_2_ were similar to those of Cu(**AIm**)_2_ and Cu(**VIm**)_2_, with only non-porous structures found in the lowest 20 kJ mol^–1^ energy window. The small voids (less than 10% of unit cell volume) were only found in the predicted 2D structures of Cu(**MeIm**)_2_.

Synthetic screening by solution-based and mechanochemical methodologies (SI and Figures S17, S20, and S23) revealed two phases that showed TGA mass losses matching the formula Cu(**MeIm**)_2_ (Figure S8D). However, one of these phases has, so far, only been synthesized as an orange-colored powder of poor crystallinity, yielding PXRD diffractograms with an insufficient number of reflections for either matching against predicted structures or for direct structure determination from PXRD data (see the SI). The other phase, a green microcrystalline powder, exhibited a PXRD diffractogram, which suggested a possible match to the ninth lowest predicted structure, a putative *dia*-Cu(**MeIm**)_2_ structure with the relative lattice energy of +7.6 kJ mol^–1^ above the global energy minimum. Fine-tuning of the synthetic procedure enabled the isolation of this Cu(**MeIm**)_2_ material in phase-pure form, as confirmed by PXRD, TGA, and IR spectroscopy. Importantly, one of the modifications of the synthetic procedure also led to diffraction-quality green single crystals of Cu(**MeIm**)_2_ that were picked from a mixture with the not yet identified orange phase. Single crystal X-ray structure analysis of Cu(**MeIm**)_2_ revealed a *dia*-topology structure that was an excellent match with the ninth lowest energy structure in the CSP energy landscape. During synthetic screening, some additional crystalline phases were encountered, most of which could be ruled out as Cu(**MeIm**)_2_ MOFs either by their TGA-determined copper content or by their high solubility in organic solvent.

The presence of multiple structures below the experiment-matching one in the CSP landscape of Cu(**MeIm**)_2_ calls for a closer inspection of factors affecting their relative stabilities, notably, the effect of temperature. As the DFT electronic calculations only predict thermodynamic stability at 0 K, we performed phonon calculations for the nine lowest energy crystal structures and determined the corresponding Helmholtz vibrational free energies (SI and Table S8). This correction resulted in a dramatic improvement in the energy ranking: while the global minimum remained the same *sql*-topology 2D framework, the experimentally observed *dia*-framework became the overall second-ranked structure, positioned +3.5 kJ mol^–1^ above the global minimum.

Unlike in the cases of Cu(**VIm**)_2_ and Cu(**AIm**)_2_, where the experimental structures matched with the predicted global minima, the CSP calculations suggest that the only so far observed structure of Cu(**MeIm**)_2_ might be a metastable polymorph. The existence of a 2D structure below the experimentally determined 3D structure in the CSP energy landscape of Cu(**MeIm**)_2_ might be considered unusual as 3D frameworks are often considered more stable than their 2D counterparts. However, even when vibrational entropy contribution is taken into account, the putative 2D framework is found to be more stable than the experimentally observed *dia-*Cu(**MeIm**)_2_. We have recently demonstrated the stabilization of a layered Hg(**Im**)_2_ framework^49^ over its 3D polymorph. In such a scenario, additional experiments may reveal the conditions necessary to synthesize and isolate the global minimum predicted *sql*-Cu(**MeIm**)_2_ structure. This possibility highlights the potential of MOF CSP methods to reveal additional lower-energy structures and suggest directions for future experimental exploration.

The lowest-energy predicted structures of Cu(**AIm**)_2_ and Cu(**VIm**)_2_ are isomorphous and match the respective experimentally observed ones. However, among the predicted structures of Cu(**AIm**)_2_ and Cu(**VIm**)_2_ we also find structures of *Fdd*2 symmetry that are isomorphous with the experimentally observed Cu(**MeIm**)_2_ structure. For Cu(**AIm**)_2_, such a structure is found at +30.2 kJ mol^–1^ with respect to the global minimum, while for Cu(**VIm**)_2_, the corresponding structure is at +23.4 kJ mol^–1^ (Tables S2 and S4). Conversely, the CSP landscape for Cu(**MeIm**)_2_ also contains a structure isomorphous to the one experimentally observed for α-Cu(**AIm**)_2_ and Cu(**VIm**)_2_. That structure lies at +8.2 kJ mol^–1^ with respect to the global minimum (Table S6).

Our focus on Cu(**AIm**)_2_ and Cu(**VIm**)_2_ as targets for our proof-of-principle CSP-guided MOF discovery was based on their potential for hypergolic ignition. This potential was subsequently verified through standard droplet ignition tests in which a 10 μL drop of white fuming nitric acid (WFNA) oxidizer is released from a 5.0 cm height onto 5 mg samples of *dia*-Cu(**VIm**)_2_, α-Cu(**AIm**)_2_, or β-Cu(**AIm**)_2_ placed in a glass vial. Droplet testing was conducted in triplicate for each material, and the process was recorded with a high-speed (1000 frames per second) camera, which enabled the measurement of the ignition delay (ID) or the time between contact with the oxidizer and sample ignition, and a key hypergolicity parameter.

**Figure 5:**
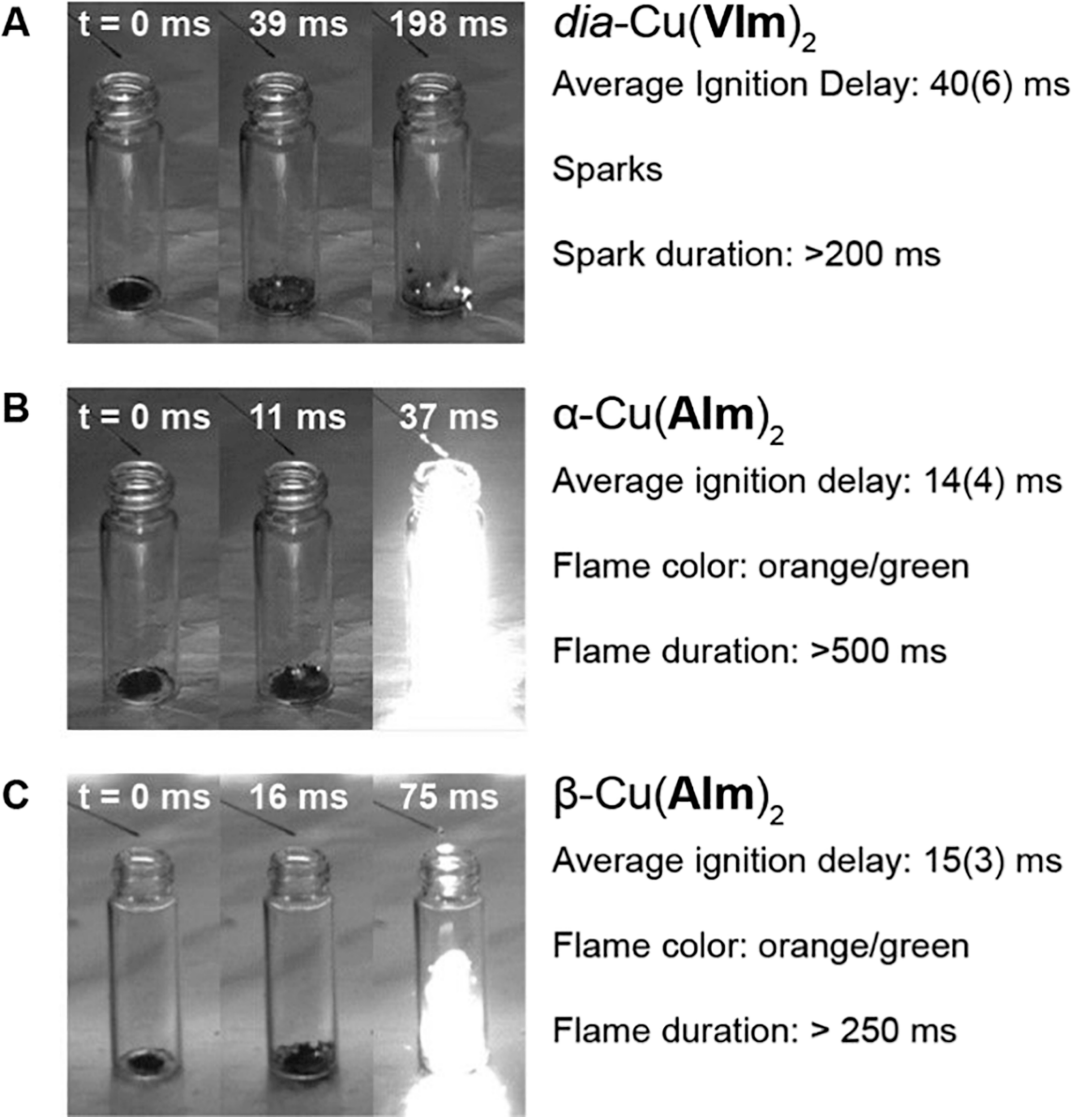
(A) *dia*-Cu(**VIm**)_2_, (B) α-Cu(**AIm**)_2_, (C) β-Cu(**AIm**)_2_. Video snapshots show the moment of WFNA drop release (0 ms), ignition event, and appearance of flame/sparks.

The droplet tests (Figure 5) on Cu(**VIm**)_2_, α-Cu(**AIm**)_2_, and β-Cu(**AIm**)_2_ revealed reliable, high-performance hypergolic behavior for each material. Specifically, α-Cu(**AIm**)_2_ and β-Cu(**AIm**)_2_ demonstrated IDs of 14(4) ms and 15(3) ms, respectively, with a longer ID of 40(6) ms recorded for Cu(**VIm**)_2_. Crucially, all three materials show IDs below 50 ms, which is the upper threshold for aerospace applications. As anticipated, Cu(**MeIm**)_2_ was not hypergolic.

## Conclusions

We have described the first successful use of ab initio CSP for the prediction of structures of MOFs based on previously unreported compositions. The targeted MOF compositions were based on conformationally flexible copper(II) nodes bridged by imidazolate linkers, selected because they provide access to a virtually limitless number of framework topologies, which makes accurate predictions of their extended structures through conventional MOF designs effectively impossible. Importantly, the selection of the metal and linker was also aimed toward the discovery of new materials that could exhibit ignition and combustion properties suitable for the development of new hypergolic fuels for space propulsion applications.^50^ The CSP methodology was highly successful, providing calculated phase landscapes for which the lowest- or one of the lowest-energy structures for each system was subsequently observed through experimental screening. The experimentally observed materials exhibited excellent hypergolic ignition properties suitable for fuel development, and importantly, their high combustion energies were derived immediately from predicted structures, demonstrating the use of CSP for simultaneous prediction of structures and functional properties of novel phases.

While the CSP derivation was not possible for a mechanically induced polymorph of one of the materials compositions, due to low symmetry and structural complexity, this structure was computationally generated through the application of the unit cell distortion analysis, providing not only a means to expand the scope of CSP for MOFs but potentially also to provide an ab initio approach to mechanochemically induced polymorphs in other types of systems. We believe that the herein described distortion analysis might become a part of a general protocol for analysis of structures generated by the WAM + AIRSS methodology, permitting systematic targeting of lower-symmetry structures.

Overall, this report outlines a new way to design MOFs, through the power and broad scope of ab initio CSP calculations, which have already benefited pharmaceutical research, battery materials, and porous molecular solids but have been lacking in the area of coordination frameworks. Given the many advanced approaches that tie expected MOF properties to their structure, the ability to predict MOF structures from first principles creates an opportunity to simultaneously predict MOF structures and screen their properties before engaging in experimental work. Such ab initio MOF design, herein demonstrated through the calculation of combustion energies for the predicted structures, provides the opportunity to reduce the time and effort dedicated to the synthesis of trial MOF candidates for a given application while providing the largest possible space for materials and structure discovery. We are confident that this will lead to a major advance in MOF design, coupled with improved understanding of structure–property relationships.

## Methods

### Ab Initio Crystal Structure Prediction of the Copper(II)-Based ZIFs

The CSP calculations are based on our previously developed method combining the ab initio random structure search (AIRSS)^51^ algorithm with the Wyckoff alignment of molecules (WAM).^26^ For each of the Cu(**AIm**)_2_, Cu(**VIm**)_2_, and Cu(**MeIm**)_2_ ZIFs, 1000, 2000, 3000, and 4000 structures were generated containing 1, 2, 3, and 4 ZIF formula units per crystallographic primitive cell, respectively. All geometry optimizations were carried out using plane-wave periodic density functional theory (DFT) calculations in the code CASTEP^40^ using a PBE functional^38^ with Grimme D2 dispersion correction.^39^ The plane-wave cutoff was initially set to 400 eV, and the Brillouin zone was sampled with a 2π × 0.07 Å^–1^ k-point grid spacing. The ultrasoft pseudopotentials were used from the internal QC5 library of CASTEP. The geometry convergence criteria were set as follows: maximum energy change 2 × 10^–5^ eV atom^–1^; maximum atomic force 0.05 eV Å^–1^; maximum atom displacement 10^–3^ Å; maximum residual stress 0.1 GPa. The predicted structures were energy-ranked, and duplicates were removed using the COMPACK^52^ clustering algorithm accessed via CSD Python API.^12^

The clustered structures within an energy window of 100 kJ mol^–1^ from the global energy minimum were then reoptimized with a method shown to be more accurate in our previous benchmarks of periodic DFT calculations against experimental calorimetric measurements.^46^ In this energy model, the PBE functional was combined with the many-body dispersion (MBD*) correction scheme.^41−43^ The plane-wave cutoff was raised to 700 eV. The reoptimized structures were energy-ranked and clustered once again. The final set of structures was analyzed by PLATON^53^ to determine void volumes, packing coefficients, and metal coordination geometries, while network topologies were analyzed using the program ToposPro.^54^ More details of the CSP procedure can be found in the SI.

### Symmetry Distortion Analysis

Symmetry perturbation analysis was used to derive the crystal structure of β-Cu(**AIm**)_2_ from the predicted structure of α-Cu(**AIm**)_2_. The predicted structure was subjected to perturbation analysis using the generate_strain.py script available within the CASTEP distribution. This script produced 12 symmetry-independent distortions of the original unit cell. The perturbed structures were then geometry-optimized in CASTEP using the PBE + MBD* energy model, same as the one used in the final energy ranking of the predicted structures. Among these perturbed structures, three were found to match the experimental PXRD pattern of β-Cu(**AIm**)_2_. Similar distortion analyses were then performed for the predicted structures of *dia*-Cu(**VIm**)2 and *dia*-Cu(**MeIm**)2. More details of the distortion analysis methodology, together with the energies of the perturbed structures is given in the SI and in Tables S3, S5, and S7.

### Experimental Methods

All experimentally determined crystal structures for the copper(II)-based ZIFs are provided in the SI in CIF format. They have also been deposited in the Cambridge Crystallographic Data Centre (CCDC codes 2176636–2176639).

Details for all experimental procedures can be found in the SI. Synthetic screening of the copper(II)-based ZIF systems were undertaken by both mechanochemical and solution-based (aqueous and solvothermal) methods.

In a typical mechanochemistry experiment, a copper(II) source and an imidazole (2 equivalents) were loaded in a 15 mL stainless steel vessel containing stainless steel milling balls and milled using a Form-Tech Scientific FTS-1000 shaker mill for 30 min at 30 Hz. In some cases, small amounts of liquid and salt additives were added to mimic previously reported syntheses of ZIFs by ball-milling.^55^ Milling products were analyzed as is without further purification (SI).

A typical solvothermal synthesis was conducted by dissolving a copper(II) salt and an imidazole in either dimethylformamide (DMF) or *N*-methyl-2-pyrollidone (NMP), sealing the solution in a glass pressure vessel, and heating the vessel to 100 °C or 140 °C for 24 h. Any precipitate formed was collected by filtration and rinsed with water and then acetone before analysis (SI).In a typical aqueous synthesis, solid imidazole was added rapidly to a stirred aqueous solution of either copper(II) sulfate pentahydrate and aqueous ammonia or Cu(NH_3_)_4_SO_4_·H_2_O. Any precipitate was collected by centrifugation and washed with water and then acetone before analysis (SI).

The syntheses of phase-pure microcrystalline powders and single crystals for each copper(II)-based ZIF were adapted from the aqueous screening syntheses, and the parameters for these reactions are provided in the SI.

All microcrystalline powders were analyzed by powder X-ray diffraction (SI). Select samples were analyzed by Fourier transform attenuated total reflectance spectroscopy (FTIR), coupled thermogravimetric analysis and differential scanning calorimetry (TGA/DSC), scanning electron microscopy (SEM), and nitrogen sorption at 77 K (see the SI).

### Hypergolic Drop-Testing of the Copper(II)-Based ZIFs

Hypergolic tests were performed using a standardized drop-test setup described previously.^56^ A 5 mg portion of a copper(II)-based ZIF placed in a glass vial, and a 10 μL drop of white fuming nitric acid is released from a syringe 5 cm above the sample. A Redlake MotionPro Y4 high-speed camera collecting images at 1000 frames per second is used to determine the time between contact of the white fuming nitric acid with the sample and the first signs of ignition, the ignition delay (ID). This data is collected in triplicate to determine the ID with an associated standard deviation, reported in parentheses next to the mean value.

## Supporting Information Available

Detailed description of the computational methods; Synthetic screening details of Cu(II)-based ZIFs; crystallographic information of Cu(II)-based ZIFs; FTIR-ATR and ^1^H NMR spectra of Cu(II)-based ZIFs; TGA/DSC analysis; N2 gas sorption isotherms of Cu(II)-based ZIFs; PXRD analysis of all Cu(II)-based ZIFs; SEM images of the microcrystalline Cu(II)-based ZIFs; energies of all predicted structures (PDF)
Predicted crystal structures of the Cu(II)-based ZIFs (CIF) (CIF) (CIF)

## Supplementary Material

ja2c12095_si_001.pdf

ja2c12095_si_002.cif

ja2c12095_si_003.cif

ja2c12095_si_004.cif

## Abbreviations

CSP: crystal structure prediction
MOF: metal–organic frameworks
ZIF: zeolitic imidazolate frameworks
MAF: metal azolate framework
AIRSS: ab initio random structure search
DFT: density functional theory
MBD*: many-body dispersion
PXRD: powder X-ray diffraction
CSD: Cambridge Structural Database
