# Supplementary material for: Experimentally Validated Ab Initio Crystal Structure Prediction of Novel Metal–Organic Framework Materials
Source: J Am Chem Soc. 2023 Jan 31;145(6):3515–25. doi: 10.1021/jacs.2c12095 (PMC9936577; doi:10.1021/jacs.2c12095)
Supplement: Supplementary file 1 — ja2c12095_si_001.pdf [file ja2c12095_si_001.pdf]

Supplementary Information

Experimentally-validated *ab initio* crystal structure prediction of novel metal-organic framework materials

Yizhi Xu[1†], Joseph M. Marrett[2†], Hatem M. Titi[2], James P. Darby[3], Andrew J. Morris[4]*, Tomislav Friščić[2]*, Mihails Arhangelskis[1]*

[1]Faculty of Chemistry, University of Warsaw; 1 Pasteura Street, Warsaw 02-093, Poland.

[2]Department of Chemistry, McGill University; 801 Sherbrooke Street West, Montréal, Québec H3A 0B8, Canada.

[3]Department of Engineering, University of Cambridge; Trumpington Street, Cambridge CB2 1PZ, UK.

[4]School of Metallurgy and Materials, University of Birmingham; Edgbaston, Birmingham B15 2TT, UK.

[†]These authors contributed equally to this work

***Corresponding authors:** a.j.morris.1@bham.ac.uk (AJM), tomislav.friscic@mcgill.ca (TF), m.arhangelskis@uw.edu.pl (MA).

Table of contents

S.1. Computational Methods 2

S.2. Materials 23

S.3. Experimental methods 23

S.4. Synthetic screening of copper-(II) based ZIF systems 25

S.5. Crystallographic information 28

S.6. FTIR-ATR spectra of copper(II)-based ZIFs 30

S.7. ^{1}H NMR spectra of acid-digested $Cu(AIm)_2$ ZIFs 31

S.8. TGA/DSC analysis of copper(II)-based ZIFs 32

S.10. N_2 gas sorption isotherms for copper(II)-based ZIFs 34

S.11. PXRD analysis of copper(II)-based ZIFs 35

S.12. SEM images of microcrystalline copper(II)-based ZIF powders 48

S.13. References 50

S.1. Computational Methods

S.1.1. *Ab initio* Crystal Structure Prediction (CSP) of copper(II)-based ZIFs

The CSP calculations are based on our previously developed method combining *ab initio* random structure search (AIRSS)[1] algorithm with Wyckoff Alignment of Molecules (WAM).[2] AIRSS + WAM, in principle, can generate any phase landscape with only the knowledge of the atomic/molecular structure of metal nodes and linkers of the putative MOFs. Random trial crystal structures were generated using AIRSS + WAM, where the metal nodes and linkers were randomly placed within the unit cell. For each of Cu(**AIm**)$_2$, Cu(**VIm**)$_2$, and Cu(**MeIm**)$_2$ ZIFs, 1000, 2000, 3000 and 4000 structures were generated containing 1, 2, 3 and 4 ZIF formula units per crystallographic primitive cell, respectively. All geometry optimizations were carried out using plane-wave periodic density functional theory (DFT) calculations in the code CASTEP.[3] The initial geometry optimization for these trial structures was performed using PBE functional[4] with Grimme D2 dispersion correction.[5] The plane-wave cutoff was set to 400 eV and the Brilloiun zone was sampled with a 2πx0.07 $Å^{-1}$ k-point grid spacing. The ultrasoft pseudopotentials were used from the internal QC5 library of CASTEP. The geometry convergence criteria were set as follows: maximum energy change $2x10^{-5}$ eV $atom^{-1}$; maximum atomic force 0.05 eV $Å^{-1}$; maximum atom displacement 10^{-3} Å; maximum residual stress 0.1 GPa The initial optimized structures were ranked by overall lattice energy and duplicate structures were removed. Clustering of duplicate structures was performed using COMPACK[6] algorithm accessed via CSD Python API.[7]

Subsequently, an energy window of 100 kJ mol^{-1} per formula unit was chosen, so that the lower in energy structures with respect to the global minimum can be used for further analysis. To obtain more accurate energy ranking, these selected low energy structures were geometry-optimized again using a model previously shown to give more accurate relative energies for MOF polymorphs, as confirmed by experimental calorimetric measurements.[8] In this energy model the PBE functional was combined with the many-body dispersion (MBD*) correction scheme.[9-11] The plane-wave cutoff was raised to 700 eV. Additionally, spin polarization was included to set the initial magnetic configuration of all copper atoms in the unit cell to be +1(up spin). The reoptimized structures were ranked again by ascending energies and duplicated were removed. The optimized structures were subsequently analyzed in PLATON[12] to obtain solvent-accessible volumes and packing coefficients for all the predicted structures, as shown in Figures S1-S3. PLATON was also used to characterize the metal coordination geometry using the geometry index τ_4,[13] as shown in Figures 1, 3 and 4 of the main manuscript. Finally, the topologies of the putative MOF structures were analyzed using the program ToposPro.[14]

S.1.2. Symmetry distortion analysis

Symmetry perturbation analysis was used to explore the crystallographic relationship between α and β polymorphs of Cu(**AIm**)$_2$ and rationalize the formation of the β polymorph under mechanical stress. We began by taking a WAM-generated structure corresponding to α-Cu(**AIm**)$_2$ (lowest energy structure labeled *CuAIm_76_P41_zp0vgTW5* in Table S2), which was found in space group $P4_1$ with four Cu(**AIm**)$_2$ formula units per crystallographic unit cell. The structure was then subjected to perturbation analysis using the *generate_strain.py* script available within the CASTEP distribution. With the aid of this script, we obtained 12 unit cell perturbations of the original structure, where the

magnitude of the perturbation vector was set to be 3 Å. The resulting perturbed structures were subjected to CASTEP geometry optimization with PBE+MBD* method, using the following steps:

1) Optimizations of only atom coordinates, while keeping the unit cell dimensions fixed at the values generated by the perturbation script.
2) Simultaneous relaxation of unit cell parameters and atom coordinates for structures obtained in step 1)
3) Structures from step 2) were further optimized with variable unit cell, using a smaller residual stress value of 0.01 GPa (compared to default 0.1 GPa).

The 12 optimized structures, obtained this way, were energy-ranked and analyzed for the presence of duplicates. It was found that most perturbed structures, upon optimization, reverted to the original α form, yet three perturbed structures formed a distinct cluster with higher density, and energy of +4.6 kJ mol^{-1} relative to the α form. These structures were found to match the experimentally recorded PXRD pattern of β-Cu(**AIm**)$_2$, enabling experimental determination of the crystal structure of this material. The small energy difference between the α and β polymorphs of Cu(**AIm**)$_2$ explains the relative ease of polymorph transition occurring under mechanical stress. Energies and lattice parameters of all the perturbed structures of Cu(**AIm**)$_2$ are given in Table S3.

We subsequently performed similar analyses for the predicted structures of Cu(**VIm**)$_2$ and Cu(**MeIm**)$_2$. In the case of Cu(**VIm**)$_2$, the predicted global minimum structure (labeled *CuVIm_80_I41_di6Zq4zm* in Table S4), was converted to space group $P4_1$, in order to exactly follow the protocol used in the case of Cu(**AIm**)$_2$. Perturbation analysis revealed structures, which could be described as a hypothetical β form of Cu(**VIm**)$_2$. Yet, unlike the case of Cu(**AIm**)$_2$, the lattice energy of these hypothetical structures was found to be much higher, at +13.4 and +15.7 kJ mol^{-1} relative to the experimentally observed Cu(**Vim**)$_2$ structure. This higher lattice energy of these hypothetical structures explains the apparent stability of Cu(**VIm**)$_2$ under mechanical stress. Summary of the perturbation analysis of Cu(**VIm**)$_2$ is given in Table S5.

Finally, the predicted structure for Cu(**MeIm**)$_2$, matching with experiment (labeled *CuMeIm_43_Fdd2_mgWt1ZMG* in Table S6) was subjected to perturbation analysis. With the structure having *Fdd2* symmetry, the perturbation script produced 18 distinct lattices, of which 17 reverted back to the original structure upon geometry optimization. The last structure, representing perturbation C_{36} is crystallographically distinct from the original polymorph. Yet, unlike in the case of Cu(**AIm**)$_2$, this perturbation is not associated with a major change in metal coordination geometry. Based on this analysis, we do not expect Cu(**MeIm**)$_2$ to undergo polymorph transformation under mechanochemical impact. The results of perturbation analysis for Cu(**MeIm**)$_2$ are summarized in Table S7.

S.1.3. Phonon calculations

Phonon calculations were performed for the 9 predicted lowest energy structures of Cu(**MeIm**)$_2$, in order to determine vibrational entropies and Helmholtz Free energies. The calculations were performed with PBE functional and MBD*

dispersion correction, plane wave cutoff was set at 700 eV. The standard and fine FFT grid were set to 2 and 3, respectively.

Prior to phonon calculations, the atomic coordinates were re-optimized with tighter force tolerance of 0.01 eV Å^{-1}, while keeping the unit cell parameters fixed at the values from the initial optimization (section S.1.1.). Phonon calculations were performed on the optimized structures using the finite displacement method, and phonon dispersion was extrapolated using a supercell method. The resulting normal mode frequencies were used to obtain the thermodynamic functions, and vibrational Helmholtz free energies at 298 K were used to assess the thermodynamic stability of the predicted structures of Cu(**MeIm**)$_2$ at room temperature. The results of these calculations are summarized in Table S8.

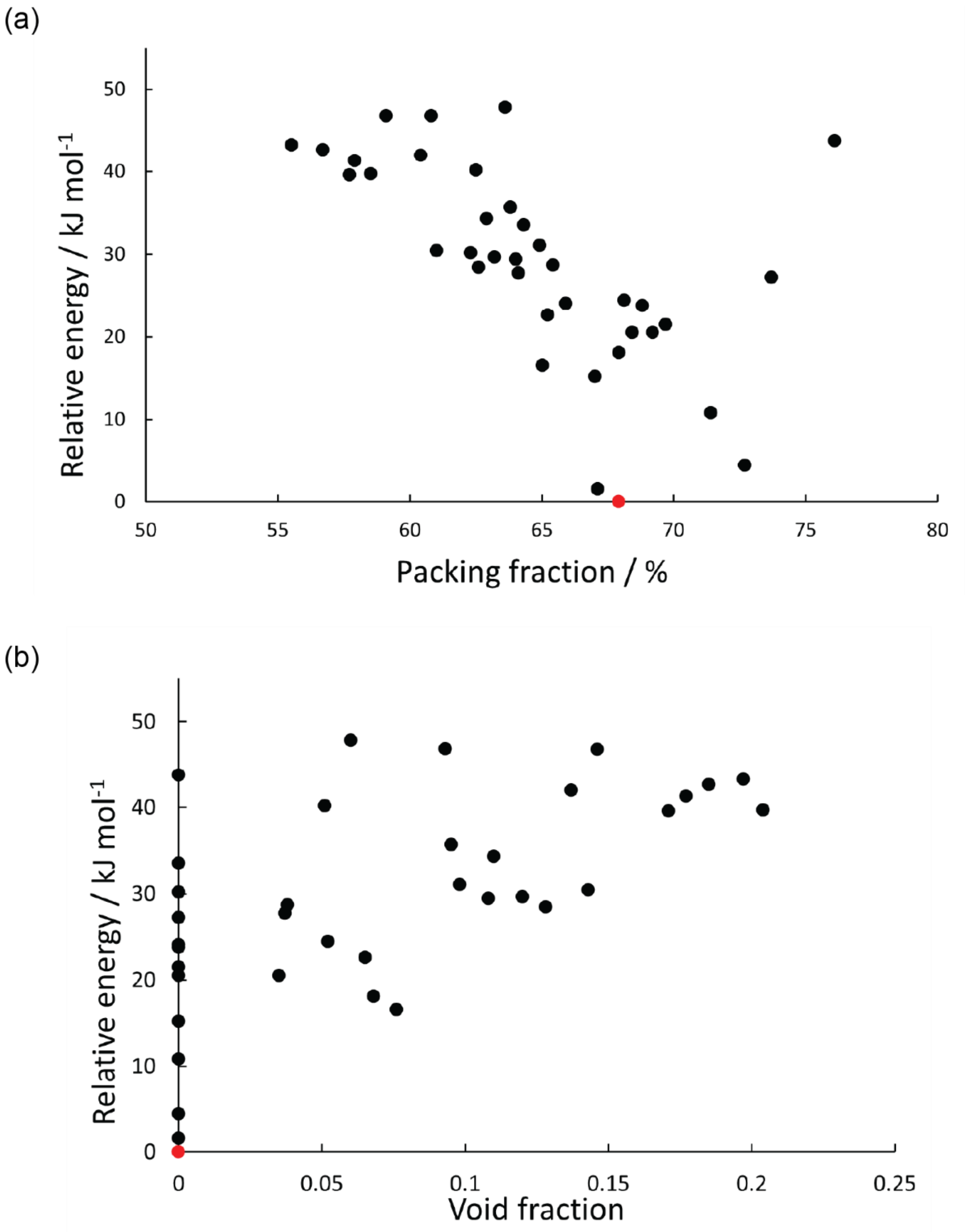

Figure S1. (a) Relative energy versus packing fraction of Cu(**AIm**)$_2$ for all the lower energy predicted structures after the second geometry optimization, with an energy window of 50 kJ mol^{-1} . The global minimum marked in red, is matching with the experimental structure. (b) Plot of the relative energy against void fraction. The calculation predicts the low energy polymorphs to be non-porous dense frameworks, consistent with the experimental crystal structures.

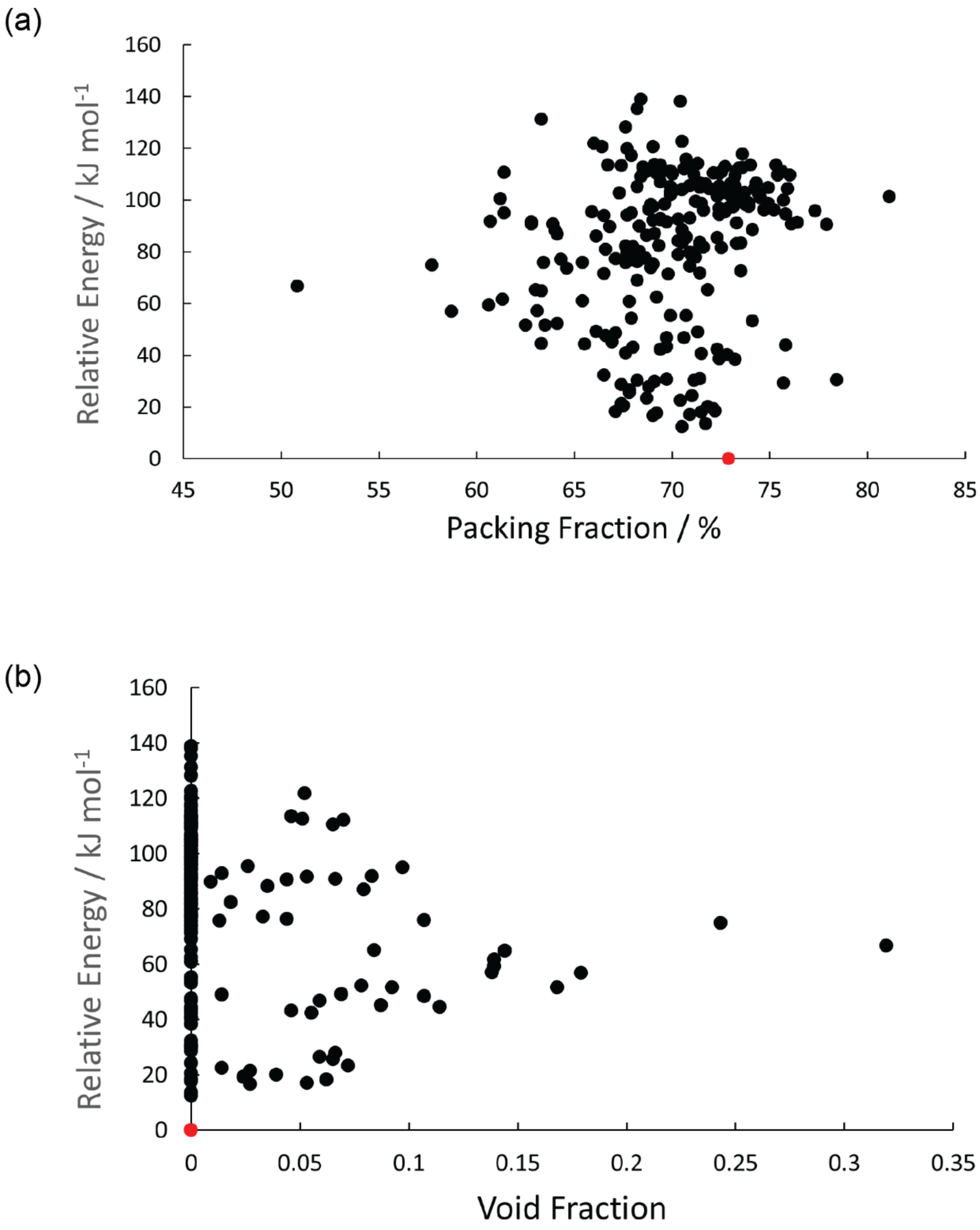

Figure S2. (a) Relative energy versus packing fraction of Cu(**VIm**)$_2$ for all the lower energy predicted structures after the second geometry optimization, with the energy window of 50 kJ mol^{-1} . The global minimum marked in red, is matching with the experimental structure. (b) Plot of the relative energy against void fraction. The calculation predicts the low energy polymorphs to be non-porous dense frameworks, consistent with the experimental crystal structures.

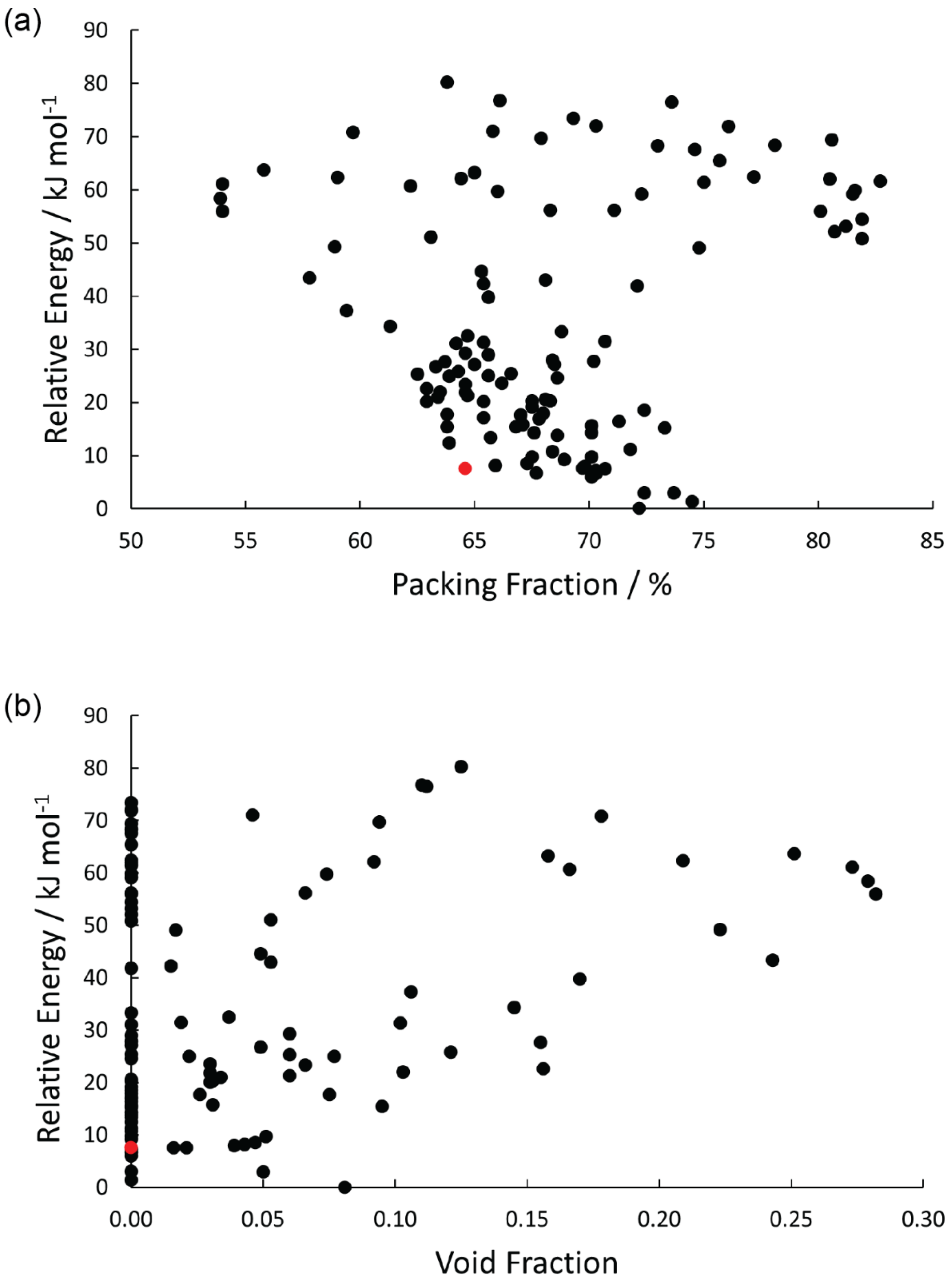

Figure S3. (a) Relative energy versus packing fraction of Cu(**MeIm**)$_2$ for all the lower energy predicted structures after the second geometry optimization, with the energy window of 50 kJ mol^{-1} . The global minimum marked in red, is matching with the experimental structure. (b) Plot of the relative energy against void fraction. The calculation predicts the low energy polymorphs to be non-porous dense frameworks, consistent with the experimental crystal structures.

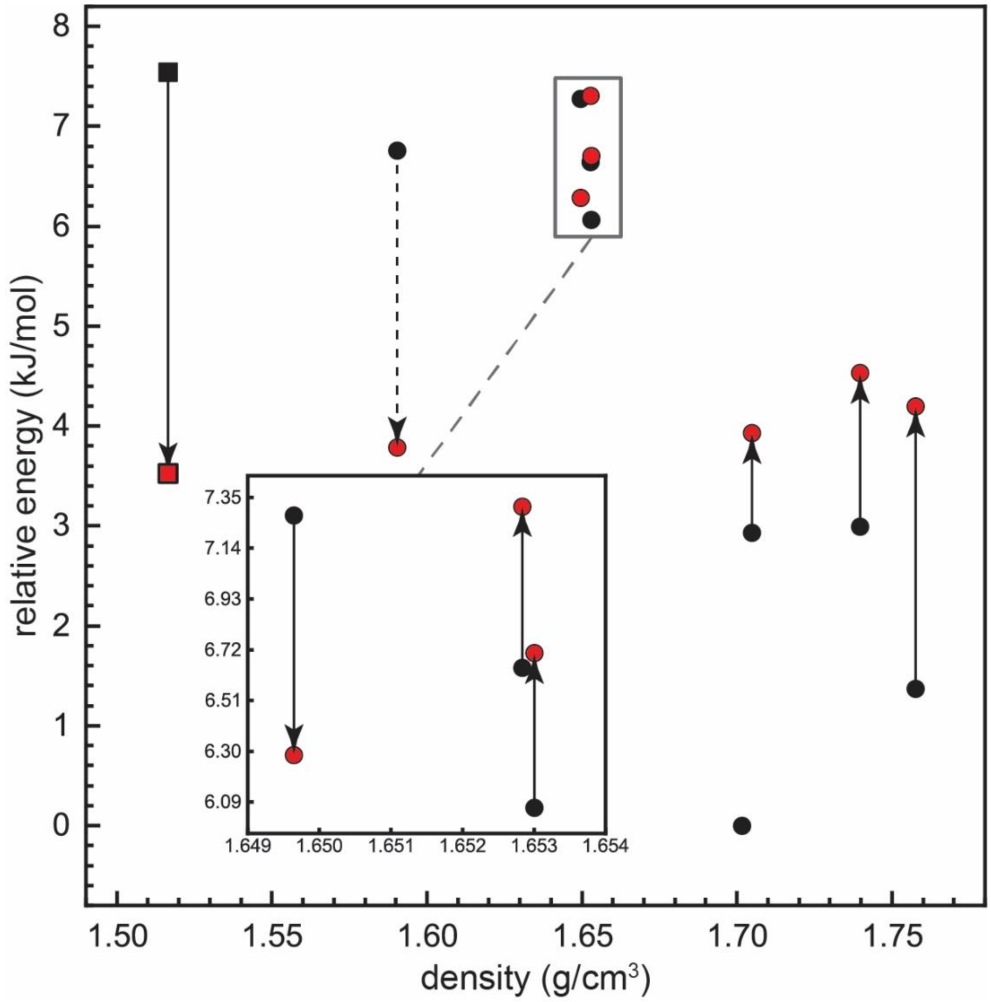

Figure S4. Relative energies of the nine lowest energy predicted structures of Cu(**MeIm**)$_2$ after correcting for vibrational entropy contribution. The direction of the arrow signifies the change in relative energy from purely electronic calculation to the calculation including Helmholtz vibrational free energy. The structure matching with experiment is designated with square markers, while all other structures are designated with circles. The one structure for which the energy change is marked with a dashed arrow, contained one imaginary frequency, the corresponding normal mode was excluded from the free energy calculation. All the other structures contained only real vibrational frequencies. All the numerical data for this plot can be found in Table S8.

S.1.4. Calculation of combustion energies and energy densities

The energies of the elements and compounds (CuO, O_2, CO_2, N_2 and H_2O) which are involved in the combustion reactions for Cu(**VIm**)$_2$, Cu(**AIm**)$_2$ and Cu(**MeIm**)$_2$ are calculated. For the gas phase components, a cubic box with 20 Å dimension has been created containing 1 molecule. The large cubic box size is aimed to minimize the interactions between the periodic images of the molecules in the neighboring boxes. During the calculations, box dimension is kept fixed. The Brillouin zone is sampled with a single Γ k-point. The combustion energies for each ZIF material are calculated based on the reaction equations in Table S1.

Table S1. Combustion reaction equations for Cu(**Vim**)$_2$, Cu(**AIm**)$_2$ and Cu(**MeIm**)$_2$.

Fuel Material	Reaction equation
Cu(**AIm**)$_2$	$Cu(C_5H_3N_2)_2$ (s) + 12 O_2 (g) → CuO (s) + 10 CO_2 (g) + 2N_2 (g) + 3H_2O (g)
Cu(**VIm**)$_2$	$Cu(C_5H_5N_2)_2$ (s) + 13 O_2 (g) → CuO (s) + 10 CO_2 (g) + 2N_2 (g) + 5H_2O (g)
Cu(**MeIm**)$_2$	$Cu(C_4H_5N_2)_2$ (g) + 11 O_2 (g) → CuO (s) + 8 CO_2 (g) + 2N_2 (g) + 5 H_2O (g)

S.1.6. CSP calculated parameters for predicted copper(II)-based ZIFs

Table S2. Calculated structure parameters, relative lattice energies, combustion energies, and topologies for the predicted Cu(**AIm**)$_2$ structures within 50 kJ mol^{-1} energy window after geometry optimization with PBE+MBD* method. In the cases, where τ_4 has two values, they represent 2 symmetry independent Cu atoms in the asymmetric unit.

Structure name	Density / g cm^{-3}	Energy per f. u. / eV	Relative energy / kJ mol^{-1}	Packing fraction	Void fraction	τ_4	ΔEc / kJ mol^{-1}	Eg / kJ g^{-1}	Ev/ kJ cm^{-3}	Topology
**CuAIm_76_P41_zp0vgTW5 Matching experimental α-Cu(AIm)$_2$**	**1.619**	**-4456.226**	**0.000**	**67.90%**	**0.00%**	**0.3**	**-5048.643**	**20.565**	**33.288**	**dia; 4/6/c1; sqc6**
CuAIm_76_P41_uHoYeZiD	1.596	-4456.209	1.578	67.10%	0.00%	0.31	-5050.220	20.571	32.841	dia; 4/6/c1; sqc6
CuAIm_14_P21_c_qIJDF6US	1.738	-4456.180	4.455	72.70%	0.00%	0.11	-5053.098	20.583	35.768	sql
CuAIm_76_P41_zp0vgTW5_cij__2__4	1.653	-4456.178	4.612	69.30%	0.00%	0.45; 0.2	-5053.255	20.584	34.024	dia; 4/6/c1; sqc6
CuAIm_144_P31_dZhj1lDZ	1.708	-4456.113	10.838	71.40%	0.00%	0.21	-5059.481	20.609	35.195	qtz; 4/6/h1
CuAIm_7_Pc_EUPJ27Tc	1.600	-4456.068	15.238	67.00%	0.00%	0.11	-5063.881	20.627	32.997	sql
CuAIm_43_Fdd2_YLwy43U2	1.549	-4456.054	16.586	65.00%	7.60%	0.42	-5065.229	20.632	31.951	sql
CuAIm_76_P41_4FSNibK3	1.621	-4456.038	18.110	67.90%	6.80%	0.57	-5066.753	20.639	33.446	dia; 4/6/c1; sqc6
CuAIm_9_Cc_Ky2YQ1db	1.654	-4456.013	20.557	69.20%	3.50%	0.46	-5069.200	20.648	34.146	sql
CuAIm_19_P212121_gOJjeJNd	1.635	-4456.013	20.566	68.40%	0.00%	0.52	-5069.208	20.649	33.767	dia; 4/6/c1; sqc6
CuAIm_41_Aba2_lWxqBGRw	1.666	-4456.003	21.521	69.70%	0.00%	0.13	-5070.164	20.652	34.407	sql
CuAIm_144_P31_nOM2L3JM	1.554	-4455.991	22.661	65.20%	6.50%	0.49	-5071.304	20.657	32.094	qtz; 4/6/h1
CuAIm_19_P212121_g0vOAUui	1.637	-4455.979	23.817	68.80%	0.00%	0.67	-5072.460	20.662	33.833	dia; 4/6/c1; sqc6
CuAIm_29_Pca21_WFF889Sc	1.571	-4455.976	24.112	65.90%	0.00%	0.11	-5072.754	20.663	32.456	sql
CuAIm_29_Pca21_uQ53uOkW	1.624	-4455.972	24.481	68.10%	5.20%	0.47	-5073.123	20.664	33.555	sql
CuAIm_19_P212121_DmSI01Ef	1.786	-4455.943	27.271	73.70%	0.00%	0.44	-5075.914	20.676	36.927	sql

CuAIm_33_Pna21_nZxCYu4w	1.524	-4455.938	27.773	64.10%	3.70%	0.55	-5076.416	20.678	31.507	dia; 4/6/c1; sqc6
CuAIm_13_P2_c_FjprQgem	1.493	-4455.931	28.474	62.60%	12.80%	0.45	-5077.116	20.681	30.885	sql
CuAIm_7_Pc_SfzvGOdH	1.559	-4455.928	28.719	65.40%	3.80%	0.16	-5077.361	20.682	32.247	sql
CuAIm_82_I-4_pAmrXduD	1.529	-4455.921	29.455	64.00%	10.80%	0.42; 0.52	-5078.098	20.685	31.63	sql
CuAIm_5_C2_C2jzLum9	1.512	-4455.918	29.689	63.20%	12.00%	0.47	-5078.332	20.686	31.274	sql
**CuAIm_43_Fdd2_iam9j16B Isomorphous with experimental Cu(MeIm)$_2$**	**1.486**	**-4455.913**	**30.206**	**62.30%**	**0.00%**	**0.63**	**-5078.848**	**20.688**	**30.741**	**dia; 4/6/c1; sqc6**
CuAIm_5_C2_M4kyTiNG	1.453	-4455.910	30.441	61.00%	14.30%	0.46	-5079.084	20.689	30.064	sql
CuAIm_75_P4_PdcBNwPY	1.547	-4455.903	31.105	64.90%	9.80%	0.17	-5079.748	20.691	32.012	sql
CuAIm_14_P21_c_Up8WCGm0	1.532	-4455.878	33.583	64.30%	0.00%	0	-5082.225	20.702	31.715	sql
CuAIm_45_Iba2_hvWzZ0Yo	1.496	-4455.870	34.338	62.90%	11.00%	0.16	-5082.980	20.705	30.982	sql
CuAIm_79_I4_bBlIgrTS	1.523	-4455.856	35.687	63.80%	9.50%	0.21; 0.11	-5084.330	20.71	31.541	sql
CuAIm_28_Pma2_wgJepkIh	1.376	-4455.816	39.584	57.70%	17.10%	0.44	-5088.227	20.726	28.521	sql
CuAIm_39_Abm2_0xn5y784	1.395	-4455.814	39.719	58.50%	20.40%	0	-5088.362	20.727	28.907	sql
CuAIm_5_C2_A7a4KdTF	1.493	-4455.809	40.205	62.50%	5.10%	0.49	-5088.847	20.729	30.949	dia; 4/6/c1; sqc6
CuAIm_77_P42_HQ8wULIE	1.381	-4455.797	41.335	57.90%	17.70%	0.5	-5089.978	20.733	28.626	sql
CuAIm_29_Pca21_0Izra9lx	1.441	-4455.790	42.007	60.40%	13.70%	0.02	-5090.650	20.736	29.872	sql
CuAIm_81_P-4_Imll7sob	1.354	-4455.783	42.681	56.70%	18.50%	0.47	-5091.324	20.739	28.088	sql
CuAIm_21_C222_1SAF10m5	1.324	-4455.777	43.288	55.50%	19.70%	0.41	-5091.931	20.741	27.47	sql
CuAIm_76_P41_RIWaRaTG	1.830	-4455.772	43.807	76.10%	0.00%	0.39	-5092.449	20.743	37.96	unc; 4/6/t1
CuAIm_144_P31_Byswu5Pi	1.452	-4455.740	46.827	60.80%	14.60%	0.81	-5095.470	20.755	30.134	qtz; 4/6/h1
CuAIm_24_I212121_OMn5Cr5E	1.408	-4455.740	46.848	59.10%	9.30%	0.72	-5095.491	20.756	29.227	dia; 4/6/c1; sqc6
CuAIm_29_Pca21_GFHNldxB	1.517	-4455.730	47.857	63.60%	6.00%	0.41	-5096.499	20.760	31.502	sql

Table S3. Lattice distortion analysis of α-Cu(**AIm**)$_2$. Structures found identical based on packing similarity analysis are highlighted with the same color. The cluster of structures colored in green is matching the original α-Cu(**AIm**)$_2$ structure, whereas structures matching β-Cu(**AIm**)$_2$ are highlighted in blue (perturbations C_{24}, C_{26} and C_{25}). Finally, the highest energy perturbation C_{16} led to the breakage of Cu-N covalent bonds, and collapse of the ZIF structure.

Perturbation index, C_{ij}	Unit cell parameters							Density / g cm^{-3}	Space group	Energy per f. u. / eV	Relative energy / kJ mol^{-1}
	a / Å	b / Å	c / Å	α / °	β / °	γ / °	V / Å^3				
**Original structure**	**7.454**	**7.454**	**18.148**	**90**	**90**	**90**	**1008.314**	**1.619**	**$P4_1$**	**-4456.226**	**0.000**
C_{22}	7.516	7.575	18.116	90	90	89.349	1031.479	1.582	$P112_1$	-4456.222	0.398
C_{11}	7.489	7.539	18.081	89.981	89.566	89.297	1020.694	1.599	P1	-4456.221	0.444
C_{23}	7.462	7.526	18.108	90	90	88.713	1016.710	1.605	$P112_1$	-4456.220	0.533
C_{21}	7.493	7.556	18.130	90	90	89.238	1026.409	1.590	$P112_1$	-4456.219	0.644
C_{12}	7.507	7.574	18.138	88.553	89.095	88.972	1030.647	1.584	P1	-4456.209	1.609
C_{13}	7.529	7.552	18.107	90.486	90.570	88.973	1029.258	1.586	P1	-4456.208	1.722
C_{24}	7.751	7.814	16.809	90	90	104.074	987.417	1.653	$P112_1$	-4456.178	4.631
C_{26}	7.781	7.807	16.745	90	90	104.106	986.522	1.654	$P112_1$	-4456.178	4.680
C_{25}	7.777	7.750	16.864	90	90	75.805	985.342	1.656	$P112_1$	-4456.176	4.793
C_{15}	7.682	7.696	17.778	90.638	88.686	83.172	1043.194	1.565	P1	-4456.150	7.318
C_{14}	7.418	7.489	18.053	92.130	91.142	88.179	1001.547	1.630	P1	-4456.018	20.095
C_{16}	4.403	9.366	21.288	103.686	86.368	87.212	849.334	1.922	P1	-4455.778	43.261

Table S4. Calculated structure parameters, relative lattice energies, combustion energies, and topologies for the predicted Cu(**VIm**)$_2$ structures within 50 kJ mol^{-1} energy window after geometry optimization with PBE+MBD* method. In the cases, where τ_4 has two values, they represent 2 symmetry independent Cu atoms in the asymmetric unit. The structure matching with experimental Cu(**VIm**)$_2$, as well as the structure isomorphous with experimental Cu(**MeIm**)$_2$ are highlighted in bold.

Structure name	Density / g cm^{-3}	Energy per f. u. / eV	Relative energy / kJ mol^{-1}	Packing fraction	Void fraction	τ_4	ΔEc / kJ mol^{-1}	Eg / kJ g^{-1}	Ev/ kJ cm^{-3}	Topology
**CuVIm_80_I41_di6Zq4zm Matching experimental Cu(VIm)$_2$**	**1.670**	**-4524.182**	**0.000**	**72.90%**	**0.00%**	**0.38**	**-5093.923**	**20.417**	**34.1**	**dia; 4/6/c1; sqc6**
CuVIm_30_Pnc2_er1XPOl2	1.614	-4524.053	12.443	70.50%	0.00%	0.08	-5106.365	20.466	33.035	sql
CuVIm_41_Aba2_vBgGYFDW	1.641	-4524.040	13.756	71.70%	0.00%	0.13	-5107.678	20.472	33.584	sql
CuVIm_32_Pba2_ExA6y4mB	1.582	-4524.009	16.739	69%	2.70%	0.14	-5110.661	20.484	32.402	sql
CuVIm_75_P4_MiTI4Bpb	1.620	-4524.004	17.151	70.90%	5.30%	0.08; 0.18	-5111.073	20.485	33.183	sql
CuVIm_41_Aba2_9zLczb4n	1.578	-4523.999	17.685	69.20%	0.00%	0.14	-5111.608	20.487	32.326	sql
CuVIm_144_P31_zkF28KNL	1.638	-4523.993	18.236	71.50%	0.00%	0.56	-5112.159	20.49	33.568	qtz; 4/6/h1
CuVIm_80_I41_DpqbdDVE	1.534	-4523.992	18.358	67.10%	6.20%	0.45	-5112.280	20.49	31.434	dia; 4/6/c1; sqc6
CuVIm_18_P21212_FnN5D1Qj	1.651	-4523.990	18.558	72.20%	0.00%	0.41; 0.14	-5112.481	20.491	33.827	sql
CuVIm_75_P4_f0Rd48ti	1.651	-4523.982	19.354	72.10%	2.40%	0.19; 0.08	-5113.277	20.494	33.843	sql
CuVIm_75_P4_UX8CvTMi	1.647	-4523.973	20.172	71.80%	3.90%	0.22; 0.07	-5114.095	20.497	33.761	sql
CuVIm_45_Iba2_LGcvZBfF	1.543	-4523.969	20.551	67.50%	0.00%	0.14	-5114.474	20.499	31.625	sql
CuVIm_45_Iba2_uyO9RYBq	1.541	-4523.960	21.456	67.40%	2.70%	0.14	-5115.379	20.503	31.598	sql
CuVIm_81_P-4_Som4YWqZ	1.605	-4523.949	22.527	70.40%	1.40%	0.39	-5116.449	20.507	32.915	sql
**CuVIm_43_Fdd2_kLxtMBTy Isomorphous with experimental Cu(MeIm)$_2$**	**1.574**	**-4523.940**	**23.361**	**68.70%**	**7.20%**	**0.55**	**-5117.284**	**20.51**	**32.291**	**dia; 4/6/c1; sqc6**
CuVIm_29_Pca21_n3Qwkn5T	1.624	-4523.929	24.393	71.00%	0.00%	0.32	-5118.315	20.514	33.309	sql

CuVIm_79_I4_ZZ1Gqkj7	1.548	-4523.916	25.680	67.80%	6.50%	0.13; 0.14	-5119.603	20.519	31.762	sql
CuVIm_79_I4_tRCwWJg4	1.548	-4523.906	26.613	67.80%	5.90%	0.12; 0.15	-5120.536	20.523	31.772	sql
CuVIm_41_Aba2_g5gY7l9h	1.583	-4523.893	27.910	68.80%	6.60%	0.15	-5121.833	20.528	32.498	sql
CuVIm_30_Pnc2_xNXogKlK	1.541	-4523.884	28.808	67.40%	0.00%	0.14	-5122.731	20.532	31.63	sql
CuVIm_14_P21_c_9kB7dEvm	1.725	-4523.878	29.386	75.70%	0.00%	0	-5123.309	20.534	35.429	sql
CuVIm_30_Pnc2_UAN961md	1.575	-4523.871	30.006	69.10%	0.00%	0.16	-5123.929	20.537	32.345	sql
CuVIm_144_P31_rsynL5sx	1.626	-4523.868	30.265	71.10%	0.00%	0.6	-5124.187	20.538	33.385	qtz; 4/6/h1
CuVIm_41_Aba2_a6Pdz99p	1.562	-4523.867	30.368	68.20%	0.00%	0.17	-5124.290	20.538	32.09	sql
CuVIm_14_P21_c_zqNaNitT	1.791	-4523.866	30.480	78.40%	0.00%	0	-5124.402	20.539	36.776	sql
CuVIm_14_P21_c_pkaCbdyJ	1.594	-4523.863	30.812	69.70%	0.00%	0	-5124.735	20.54	32.739	sql
CuVIm_144_P31_i3hufi9j	1.631	-4523.861	30.969	71.40%	0.00%	0.66	-5124.892	20.541	33.51	qtz; 4/6/h1
CuVIm_144_P31_7GS7PKq4	1.522	-4523.846	32.387	66.50%	0.00%	0.55	-5126.310	20.546	31.271	qtz; 4/6/h1
CuVIm_27_Pcc2_ASUS41pH	1.672	-4523.784	38.439	73.20%	0.00%	0.41	-5132.361	20.571	34.39	sql
CuVIm_43_Fdd2_cFg2amJx	1.656	-4523.781	38.668	72.40%	0.00%	0.42	-5132.590	20.572	34.066	dia; 4/6/c1; sqc6
CuVIm_43_Fdd2_cV7gL8a9	1.667	-4523.764	40.383	72.80%	0.00%	0.43	-5134.305	20.578	34.305	dia; 4/6/c1; sqc6
CuVIm_144_P31_sJjq4Lh9	1.634	-4523.760	40.753	71.50%	0.00%	0.48	-5134.675	20.58	33.626	qtz; 4/6/h1
CuVIm_43_Fdd2_YZJZXBs2	1.546	-4523.759	40.856	67.60%	0.00%	0.647	-5134.779	20.58	31.821	dia; 4/6/c1; sqc6
CuVIm_19_P212121_5GO2pbjY	1.594	-4523.743	42.370	69.40%	5.50%	0.24	-5136.293	20.586	32.805	sql
CuVIm_43_Fdd2_AcWFExUZ	1.651	-4523.743	42.407	72.30%	0.00%	0.53	-5136.330	20.586	33.994	dia; 4/6/c1; sqc6
CuVIm_43_Fdd2_pETIakVh	1.559	-4523.735	43.187	68.00%	4.60%	0.47	-5137.109	20.59	32.099	sql
CuVIm_76_P41_PfXPSJxR	1.591	-4523.732	43.470	69.70%	0.00%	0.69	-5137.393	20.591	32.756	dia; 4/6/c1; sqc6
CuVIm_82_I-4_3aapfp4F	1.727	-4523.726	43.980	75.80%	0.00%	0.75; 0.17	-5137.903	20.593	35.565	dia; 4/6/c1; sqc6

CuVIm_76_P41_E6ZoKZHC	1.499	-4523.721	44.469	65.50%	0.00%	0.71	-5138.392	20.595	30.87	dia; 4/6/c1; sqc6
CuVIm_14_P21_c_f2jDmqyf	1.449	-4523.719	44.694	63.30%	11.40%	0	-5138.616	20.596	29.845	sql
CuVIm_20_C2221_oulPpEWl	1.535	-4523.714	45.189	66.90%	8.70%	0.43	-5139.111	20.598	31.619	sql
CuVIm_20_C2221_7QK1uDQ6	1.621	-4523.697	46.836	70.60%	0.00%	0.48	-5140.758	20.604	33.399	sql
CuVIm_19_P212121_WC2HkzHh	1.592	-4523.696	46.859	69.70%	5.90%	0.26	-5140.782	20.604	32.793	sql
CuVIm_43_Fdd2_HP7z44yJ	1.529	-4523.688	47.674	66.60%	0.00%	0.49	-5141.597	20.608	31.5	dia; 4/6/c1; sqc6
CuVIm_29_Pca21_nqJfnl7g	1.538	-4523.679	48.572	67.10%	10.70%	0.37	-5142.495	20.611	31.704	sql
CuVIm_82_I-4_MVQQ4FbV	1.631	-4523.674	49.074	71.30%	1.40%	0.26; 0.34	-5142.997	20.613	33.613	sql
CuVIm_5_C2_v5RqP7Zh	1.515	-4523.672	49.228	66.10%	6.90%	0.43	-5143.150	20.614	31.221	sql

Table S5. Lattice distortion analysis of Cu(**VIm**)$_2$. Structures found identical based on packing similarity analysis are highlighted with the same color. The cluster of structures colored in green is matching the original Cu(V**Im**)$_2$ structure, whereas structures that could potentially be regarded as other polymorphs of Cu(**VIm**)$_2$ are highlighted in blue and grey (perturbations C_{25}, C_{26} and C_{24}). Finally, the highest energy perturbation C_{16} led to the breakage of Cu-N covalent bonds, and collapse of the ZIF structure.

Perturbation	Unit cell parameters							Density / g cm^{-3}	Space group	Energy per f. u. / eV	Relative energy / kJ mol^{-1}
	a / Å	b / Å	c / Å	α / °	β / °	γ / °	V / Å^3				
**Original structure**	**7.263**	**7.263**	**18.848**	**90**	**90**	**90**	**994.160**	**1.670**	**I4$_1$**	**-4524.182**	**0.000**
C_{23}	7.289	7.202	18.896	90	90	89.149	991.897	1.672	P112$_1$	-4524.193	-1.076
C_{21}	7.231	7.247	18.918	90	90	89.383	991.271	1.674	P112$_1$	-4524.192	-0.994
C_{22}	7.152	7.322	18.906	90	90	90.467	989.966	1.676	P112$_1$	-4524.191	-0.902
C_{12}	7.151	7.340	18.895	89.946	89.941	90.800	991.650	1.673	P1	-4524.186	-0.359
C_{13}	7.282	7.192	18.905	89.960	89.525	89.250	990.041	1.676	P1	-4524.182	0.014
C_{11}	7.303	7.175	18.898	89.902	90.079	88.504	989.781	1.676	P1	-4524.180	0.183
C_{14}	7.170	7.297	18.904	90.028	89.805	90.295	988.997	1.677	P1	-4524.179	0.263
C_{15}	7.246	7.205	18.909	89.998	89.277	89.565	987.037	1.681	P1	-4524.176	0.538
C_{25}	7.731	7.707	17.781	90	90	74.677	1021.815	1.624	P112$_1$	-4524.043	13.390
C_{26}	8.038	7.656	16.622	90	90	110.954	955.145	1.737	P112$_1$	-4524.019	15.698
C_{24}	8.040	7.646	16.508	90	90	110.002	953.593	1.740	P112$_1$	-4524.016	16.065
C_{16}	5.027	9.587	19.558	95.010	102.682	97.489	905.209	1.833	P1	-4522.734	139.751

Table S6. Calculated structure parameters, relative lattice energies, combustion energies and topologies for the predicted Cu(**MeIm**)$_2$ within 50 kJ mol^{-1} energy window after geometry optimization with PBE+MBD* method. In the cases, where τ_4 has two values, they represent 2 symmetry independent Cu atoms in the asymmetric unit. The structure matching with experimental Cu(**MeIm**)$_2$, as well as the structure isomorphous with α-Cu(**AIm**)$_2$ and Cu(**VIm**)$_2$ are highlighted in bold.

Structure name	Density / g cm^{-3}	Energy per f. u. / eV	Relative energy / kJ mol^{-1}	Packing fraction	Void Fraction	τ_4	ΔEc / kJ mol^{-1}	Eg / kJ g^{-1}	Ev/ kJ cm^{-3}	Topology
CuMeIm_32_Pba2_MNmaGIyi	1.702	-4210.354	0.000	72.20%	8.10%	0.13	-4178.877	18.697	31.818	sql
CuMeIm_29_Pca21_M8uKqnUb	1.758	-4210.340	1.372	74.50%	0.00%	0.14	-4180.249	18.704	32.874	sql
CuMeIm_85_P4_n_GJItVWpl	1.705	-4210.324	2.935	72.40%	5.00%	0.12	-4181.812	18.711	31.9	sql
CuMeIm_92_P41212_TGAEXgKm	1.740	-4210.323	2.993	73.70%	0.00%	0.4	-4181.870	18.711	32.551	dia; 4/6/c1; sqc6
CuMeIm_92_P41212_Xvcse2hO	1.653	-4210.291	6.066	70.10%	0.00%	0.31	-4184.943	18.725	30.952	dia; 4/6/c1; sqc6
CuMeIm_7_Pc_xBFCKyW5	1.653	-4210.285	6.645	70.30%	0.00%	0.1	-4185.522	18.727	30.953	sql
CuMeIm_92_P41212_aXfR9xrL	1.590	-4210.284	6.761	67.70%	0.00%	0.39	-4185.638	18.728	29.785	dia; 4/6/c1; sqc6
CuMeIm_7_Pc_BsS1f4UY	1.650	-4210.279	7.276	70.30%	0.00%	0.13	-4186.153	18.73	30.898	sql
**CuMeIm_43_Fdd2_mgWt1ZMG Matching experimental Cu(MeIm)$_2$**	**1.516**	**-4210.276**	**7.547**	**64.60%**	**0.00%**	**0.51**	**-4186.424**	**18.731**	**28.404**	**dia; 4/6/c1; sqc6**
CuMeIm_30_Pnc2_JB5MI41c	1.666	-4210.276	7.571	70.70%	1.60%	0.12	-4186.449	18.731	31.202	sql
CuMeIm_90_P4212_R3q15HPx	1.642	-4210.275	7.598	69.70%	2.10%	0.1; 0.45	-4186.475	18.731	30.754	sql
CuMeIm_110_I41cd_etmTmbvC	1.643	-4210.272	7.931	69.80%	3.90%	0.14	-4186.808	18.733	30.787	sql
**CuMeIm_80_I41_FbJs8Pgm Isomorphous with experimental α-Cu(AIm)$_2$ and Cu(VIm)$_2$**	**1.552**	**-4210.269**	**8.179**	**65.90%**	**4.30%**	**0.35**	**-4187.056**	**18.734**	**29.068**	**dia; 4/6/c1; sqc6**
CuMeIm_22_F222_0RN0wSOf	1.585	-4210.265	8.538	67.30%	4.70%	0.44; 0.29	-4187.415	18.736	29.704	dia; 4/6/c1; sqc6
CuMeIm_30_Pnc2_o5RmUw9z	1.623	-4210.258	9.242	68.90%	0.00%	0.09	-4188.119	18.739	30.408	sql
CuMeIm_106_P42bc_LolJebwQ	1.588	-4210.253	9.708	67.50%	5.10%	0.13	-4188.586	18.741	29.752	sql
CuMeIm_54_Pcca_bTvUoyD2	1.649	-4210.253	9.781	70.10%	0.00%	0.13	-4188.658	18.741	30.898	sql

CuMeIm_43_Fdd2_UIXhjxRR	1.603	-4210.243	10.725	68.40%	0.00%	0.43	-4189.603	18.745	30.056	dia; 4/6/c1; sqc6
CuMeIm_41_Aba2_BTnO5ZdJ	1.697	-4210.238	11.205	71.80%	0.00%	0.11	-4190.082	18.748	31.818	sql
CuMeIm_22_F222_UMQvTIje	1.501	-4210.225	12.434	63.90%	0.00%	0.61; 0.19	-4191.311	18.753	28.145	dia; 4/6/c1; sqc6
CuMeIm_23_I222_5sWunN0d	1.549	-4210.215	13.409	65.70%	0.00%	0.44; 0.41	-4192.287	18.757	29.048	sql
CuMeIm_52_Pnna_QpFttB4K	1.616	-4210.211	13.778	68.60%	0.00%	0.12	-4192.655	18.759	30.308	sql
CuMeIm_37_Ccc2_XbzpJ1l0	1.595	-4210.206	14.260	67.60%	0.00%	0.12	-4193.137	18.761	29.925	sql
CuMeIm_120_I-4c2_VqIMdT1G	1.656	-4210.206	14.284	70.10%	0.00%	0.32	-4193.161	18.761	31.073	sql
CuMeIm_152_P3121_PI0Cvmvd	1.731	-4210.196	15.210	73.30%	0.00%	0.26	-4194.087	18.765	32.475	qtz; 4/6/h1
CuMeIm_90_P4212_xC95SPMX	1.573	-4210.194	15.466	66.80%	9.50%	0.15	-4194.343	18.767	29.518	sql
CuMeIm_171_P62_Eqz9a9Tj	1.500	-4210.190	15.467	63.80%	0.00%	0.21	-4194.698	18.768	28.152	qtz; 4/6/h1
CuMeIm_48_Pnnn_dm0R7XPM	1.650	-4210.192	15.659	70.10%	0.00%	0.37; 0.4	-4194.536	18.768	30.962	sql
CuMeIm_60_Pbcn_EDyEjih5	1.579	-4210.190	15.799	67.10%	3.10%	0.07	-4194.676	18.768	29.627	sql
CuMeIm_19_P212121_zFQkPVAD	1.677	-4210.184	16.397	71.30%	0.00%	0.45	-4195.275	18.771	31.482	dia; 4/6/c1; sqc6
CuMeIm_144_P31_3LMmF15B	1.594	-4210.178	16.975	67.80%	0.00%	0.54	-4195.853	18.773	29.93	qtz; 4/6/h1
CuMeIm_171_P62_dEyIpRm2	1.542	-4210.176	17.175	65.40%	0.00%	0.62	-4196.052	18.774	28.945	qtz; 4/6/h1
CuMeIm_91_P4122_34Iq84ad	1.571	-4210.171	17.685	67.00%	7.50%	0.48	-4196.563	18.777	29.504	sql
CuMeIm_21_C222_NHvmfzeo	1.498	-4210.170	17.711	63.80%	2.60%	0.46; 0.44	-4196.589	18.777	28.128	sql
CuMeIm_9_Cc_rHpTauWm	1.602	-4210.168	17.923	68.00%	0.00%	0.46	-4196.800	18.778	30.08	dia; 4/6/c1; sqc6
CuMeIm_85_P4_n_quZ4ihaf	1.714	-4210.162	18.513	72.40%	0.00%	0.01; 0.8	-4197.390	18.78	32.193	sql
CuMeIm_2_P-1_VRciQSb4	1.589	-4210.155	19.156	67.50%	0.00%	0	-4198.033	18.783	29.85	sql
CuMeIm_60_Pbcn_ovFYUti5	1.532	-4210.145	20.124	65.40%	3.00%	0.52	-4199.001	18.787	28.782	sql
CuMeIm_20_C2221_qn8DyNq5	1.476	-4210.144	20.217	62.90%	0.00%	0.45	-4199.095	18.788	27.722	sql

CuMeIm_21_C222_EKAfqkWL	1.606	-4210.144	20.263	68.30%	0.00%	0.31; 0.41	-4199.141	18.788	30.181	sql
CuMeIm_4_P21_kdWXlVsh	1.587	-4210.144	20.271	67.50%	3.10%	0	-4199.149	18.788	29.81	sql
CuMeIm_33_Pna21_TydTWl1K	1.601	-4210.141	20.556	68.10%	0.00%	0.55	-4199.433	18.789	30.082	dia; 4/6/c1; sqc6
CuMeIm_144_P31_oHZe9x6n	1.493	-4210.137	20.976	63.40%	3.40%	0.49	-4199.854	18.791	28.048	qtz; 4/6/h1
CuMeIm_92_P41212_hRLm2c22	1.527	-4210.134	21.255	64.70%	6.00%	0.5	-4200.133	18.793	28.688	dia; 4/6/c1; sqc6
CuMeIm_61_Pbca_QVwRt6ro	1.520	-4210.130	21.856	64.60%	3.00%	0	-4200.487	18.794	28.567	sql
CuMeIm_90_P4212_qWBjYNaS	1.489	-4210.126	21.979	63.50%	10.30%	0.71; 0	-4200.856	18.796	27.996	sql
CuMeIm_114_P-421c_YHWf7YNE	1.472	-4210.120	22.623	62.90%	15.60%	0.47	-4201.500	18.799	27.676	sql
CuMeIm_93_P4222_OQ9RTPta	1.521	-4210.112	23.363	64.60%	6.60%	0.54; 0.38	-4202.241	18.802	28.593	pts; sqc183
CuMeIm_60_Pbcn_XCRnesCS	1.556	-4210.110	23.561	66.20%	3.00%	0.01	-4202.439	18.803	29.262	sql
CuMeIm_33_Pna21_NER8bow9	1.615	-4210.099	24.635	68.60%	0.00%	0.43	-4203.512	18.808	30.368	dia; 4/6/c1; sqc6
CuMeIm_9_Cc_ri3As4Lp	1.503	-4210.095	24.948	63.90%	7.70%	0.31	-4203.825	18.809	28.277	sql
CuMeIm_93_P4222_BuDQwaAA	1.543	-4210.095	25.033	65.60%	2.20%	0.32; 0.46	-4203.910	18.809	29.032	pts; sqc183
CuMeIm_56_Pccn_53w3f4RO	1.470	-4210.090	25.298	62.50%	6.00%	0	-4204.347	18.811	27.653	sql
CuMeIm_43_Fdd2_onpZTj9N	1.565	-4210.090	25.424	66.60%	0.00%	0.75	-4204.301	18.811	29.433	dia; 4/6/c1; sqc6
CuMeIm_114_P-421c_6muodIfQ	1.510	-4210.090	25.819	64.30%	12.10%	0.51	-4204.347	18.811	28.405	sql
CuMeIm_116_P-4c2_OZE5fpfc	1.489	-4210.077	26.709	63.30%	4.90%	0.39	-4205.586	18.817	28.011	sql
CuMeIm_152_P3121_vwvToCPE	1.534	-4210.073	27.129	65.00%	0.00%	0.27	-4206.006	18.819	28.862	qtz; 4/6/h1
CuMeIm_88_I41_a_YptWdKvz	1.612	-4210.072	27.175	68.50%	0.00%	0.51; 0.53	-4206.052	18.819	30.345	sql
CuMeIm_90_P4212_FLSSpxys	1.491	-4210.068	27.616	63.70%	15.50%	0	-4206.493	18.821	28.068	sql
CuMeIm_4_P21_c727xgSz	1.649	-4210.067	27.729	70.20%	0.00%	0	-4206.606	18.822	31.035	sql
CuMeIm_34_Pnn2_vt7HWjkU	1.614	-4210.064	27.992	68.40%	0.00%	0.05; 0.93	-4206.870	18.823	30.382	dia; 4/6/c1; sqc6

CuMeIm_56_Pccn_tS8OBdpt	1.540	-4210.054	28.974	65.60%	0.00%	0	-4207.851	18.827	29.002	sql
CuMeIm_86_P42_n_MelkwI2E	1.514	-4210.050	29.309	64.60%	6.00%	0.43	-4208.187	18.829	28.509	sql
CuMeIm_92_P41212_TUmYWC8H	1.511	-4210.032	31.082	64.20%	0.00%	0.47	-4209.960	18.837	28.457	dia; 4/6/c1; sqc6
CuMeIm_33_Pna21_qgqFOcRZ	1.548	-4210.029	31.326	65.40%	10.20%	0.26	-4210.203	18.838	29.161	sql
CuMeIm_88_I41_a_sQd5YxwO	1.671	-4210.028	31.471	70.70%	1.90%	0	-4210.348	18.838	31.47	lvt; 4/4/t1; sqc176
CuMeIm_29_Pca21_h5XZ2ATF	1.521	-4210.018	32.462	64.70%	3.70%	0.4	-4211.339	18.843	28.663	sql
CuMeIm_5_C2_Q9EHDVo7	1.616	-4210.009	33.310	68.80%	0.00%	0.8; 0.12	-4212.187	18.846	30.463	dia; 4/6/c1; sqc6
CuMeIm_67_Cmma_h4wNrgCZ	1.439	-4209.999	34.290	61.30%	14.50%	0.6; 0.14	-4213.168	18.851	27.119	sql
CuMeIm_62_Pnma_ob3QZA34	1.393	-4209.967	37.293	59.40%	10.60%	0	-4216.171	18.864	26.276	sql
CuMeIm_152_P3121_o5LgK8Vm	1.548	-4209.942	39.797	65.60%	17.00%	0.49	-4218.675	18.876	29.227	qtz; 4/6/h1
CuMeIm_85_P4_n_CnlvPrlc	1.702	-4209.920	41.872	72.10%	0.00%	0.08; 0.68	-4220.749	18.885	32.137	sql
CuMeIm_112_P-42c_z68NDSbO	1.538	-4209.916	42.279	65.40%	1.50%	0.41; 0.89	-4221.157	18.887	29.053	pts; sqc183
CuMeIm_82_I-4_XtnPP0k8	1.600	-4209.910	42.982	68.10%	5.30%	0.92	-4221.714	18.889	30.223	dia; 4/6/c1; sqc6
CuMeIm_171_P62_jTw5Bcvk	1.363	-4209.904	43.393	57.80%	24.30%	0.73	-4222.270	18.892	25.756	qtz; 4/6/h1
CuMeIm_88_I41_a_ppUWToJj	1.549	-4209.892	44.612	65.30%	4.90%	0	-4223.489	18.897	29.263	lvt; 4/4/t1; sqc176
CuMeIm_85_P4_n_s17uPizd	1.790	-4209.846	49.055	74.80%	1.70%	0.24; 0.44	-4227.932	18.917	33.858	sql
CuMeIm_147_P-3_QfZOZxBG	1.382	-4209.844	49.253	58.90%	22.30%	0	-4228.130	18.918	26.149	kgm

Table S7. Lattice distortion analysis of Cu(**MeIm**)$_2$. Structures found identical to the original Fdd2 structure, are highlighted in green (all perturbations except for C_{36}). The perturbation C_{36} should be regarded as a distinct crystal structure since it does not perfectly overlay with the original structure. Unlike in the case of perturbations of Cu(**VIm**)$_2$ and Cu(**AIm**)$_2$, here we do not see significant changes in metal coordination geometry.

Perturbation	Unit cell parameters							Density / g cm^{-3}	Space group	Energy per f. u. / eV	Relative energy / kJ mol^{-1}
	a / Å	b / Å	c / Å	α / °	β / °	γ / °	V / Å^3				
**Original structure**	**15.754**	**7.657**	**16.394**	**90**	**90**	**90**	**1997.535**	**1.516**	**Fdd2**	**-4210.276**	**0.000**
C_{25}	15.151	7.786	16.950	90	90.07735	90	1999.520	1.500	F1d1	-4210.299	-2.202
C_{12}	15.177	7.800	16.895	90.86979	90	90	1999.937	1.499	Fd11	-4210.299	-2.199
C_{21}	15.162	7.812	16.891	90	89.86417	90	2000.630	1.499	F1d1	-4210.298	-2.093
C_{35}	15.174	7.783	16.925	90	90	89.88575	1998.714	1.500	F112	-4210.298	-2.078
C_{14}	15.143	7.783	16.945	90.59825	90	90	1996.867	1.502	Fd11	-4210.297	-2.073
C_{11}	15.162	7.802	16.907	90.78123	90	90	1999.959	1.499	Fd11	-4210.297	-2.060
C_{34}	15.211	7.770	16.903	90	90	90.11611	1997.809	1.501	F112	-4210.297	-1.997
C_{13}	15.202	7.817	16.846	90.84783	90	90	2001.593	1.498	Fd11	-4210.296	-1.957
C_{15}	15.210	7.804	16.838	90.98239	90	90	1998.438	1.501	Fd11	-4210.296	-1.906
C_{22}	15.223	7.753	16.895	90	90.08433	90	1994.069	1.504	F1d1	-4210.296	-1.891
C_{16}	15.152	7.778	16.939	91.00781	90	90	1995.911	1.502	Fd11	-4210.296	-1.881
C_{23}	15.014	7.838	17.002	90	89.99928	90	2000.749	1.499	F1d1	-4210.295	-1.820
C_{31}	15.179	7.798	16.881	90	90	90.10292	1998.116	1.501	F112	-4210.295	-1.795
C_{33}	15.169	7.779	16.923	90	90	89.87627	1997.046	1.502	F112	-4210.293	-1.592
C_{24}	15.255	7.804	16.779	90	89.9961	90	1997.473	1.501	F1d1	-4210.291	-1.494
C_{26}	15.253	7.794	16.787	90	89.88041	90	1995.597	1.503	F1d1	-4210.290	-1.329
C_{32}	15.593	7.723	16.560	90	90	89.92413	1994.201	1.504	F112	-4210.287	-1.057
C_{36}	14.302	7.895	17.500	90	90	92.52704	1974.083	1.519	F112	-4210.239	3.613

Table S8. Vibrational Helmholtz free energy calculations for the 9 lowest energy predicted structures of Cu(**MeIm**)$_2$ result in a re-ranking with respect to purely electronic energies shown in Table S6.

Structure name	Density / g cm^{-3}	Energy (Electronic + Vibrational) per f. u. / eV	Relative energy / kJ mol^{-1}	Relative energy change with respect to Electronic-only energy ranking (Table S6) / kJ mol^{-1}
CuMeIm_32_Pba2_MNmaGIyi	1.702	-4205.947	0.000	No change (remains a global minimum)
**CuMeIm_43_Fdd2_mgWt1ZMG (experimental matching structure)**	**1.516**	**-4205.911**	**3.525**	**-4.022**
CuMeIm_92_P41212_aXfR9xrL*	1.590	-4205.908	3.787	-2.974
CuMeIm_85_P4_n_GJItVWpl	1.705	-4205.906	3.936	+1.001
CuMeIm_29_Pca21_M8uKqnUb	1.758	-4205.904	4.199	+2.827
CuMeIm_92_P41212_TGAEXgKm	1.740	-4205.900	4.533	+1.540
CuMeIm_7_Pc_BsS1f4UY	1.650	-4205.882	6.284	-0.992
CuMeIm_92_P41212_Xvcse2hO	1.653	-4205.878	6.707	+0.661
CuMeIm_7_Pc_xBFCKyW5	1.653	-4205.871	7.312	+0.667

*Phonon calculation for this structure revealed one imaginary frequency (magnitude of 15.36 cm^{-1}), which will introduce an error in the derived value for the vibrational Helmholtz free energy. All other structures contained no imaginary frequencies.

S.2. Materials

2-methylimidazole (H**MeIm**) (99%) and was obtained from Sigma Aldrich. Copper(II) sulfate heptahydrate (≥99%) was obtained from American Chemicals Ltd. Aqueous ammonia (28%), methanol (99.8%), and sodium hydroxide were obtained from Fischer Scientific. Acetone (≥99.5%) was obtained from Caledon Chemicals. Sodium hydroxide (≥97%) was obtained from ACP Chemicals. All chemicals listed above were used as supplied without further purification. 2-vinylimidazole (H**VIm**)[15], 2-ethynylimidazole (H**AIm**)[16], and $Cu(NH_3)_4(H_2O)(SO_4)$[17] were prepared according to previous reports.

S.3. Experimental methods

S.3.1. Synthesis of microcrystalline powder of *α*-Cu(AIm)$_2$

124.8 mg of copper (II) sulfate pentahydrate (0.5 mmol) was dissolved in 10 mL of water and 1 mL of aqueous ammonia (28%) was added to form a dark blue solution. Next, 92 mg of H**AIm** (1 mmol) was added as a solid with stirring, immediately forming a burgundy precipitate. This mixture was allowed to stir for 5 minutes, then was left undisturbed for 30 minutes before the powder was isolated by filtration and rinsed with 20 mL of water on the fitted glass filter. The powder was suspended and stirred briefly in 20 mL of acetone, collected again by filtration, and dried overnight at 80 °C under vacuum.

S.3.2. Synthesis of microcrystalline powder of *β*-Cu(Aim)$_2$

60 mg of α-Cu(**AIm**)$_2$ and 30 uL of methanol were milled for 20 minutes in a 15 mL volume zirconia milling jar, using a single 1.3 g weight zirconia milling ball. Ball milling was performed at a frequency of 25 Hz. The resulting powder was collected, stirred briefly in methanol, filtered, and dried overnight at 80 °C under vacuum.

S.3.3. Synthesis of microcrystalline powder of *dia*-Cu(VIm)$_2$

245 mg of $Cu(NH_3)_4(H_2O)SO_4$ (1 mmol) and 2 mmol 197.4 mg of H**VIm** (2 mmol) were added to 1 mL of water and the resulting slurry was stirred rapidly for 30 minutes. 10 mL of water was added, and the powder was immediately collected by filtration then rinsed with 20 mL of water on the fritted glass filter. The powder was suspended and stirred briefly in 20 mL of acetone, collected again by filtration, and dried overnight at 80 °C under vacuum.

S.3.4. Synthesis of microcrystalline powder of *dia*-Cu(MeIm)$_2$

124.8 mg of copper(II) sulfate pentahydrate (0.5 mmol) was dissolved in 10 ml of water, then 3 mL of aqueous ammonia (28%) was added to form a dark blue solution. Next, 328.4 mg of 2-methylimidazole (4 mmol) was added as a solid, and dissolved with stirring. After 30 minutes of sitting, 200 µL of 25% by weight aqueous NaOH (2.5g of NaOH dissolved in 7.5 mL water) were added dropwise over 1 minute, with rapid stirring, to produce a green precipitate which was collected by filtration and rinsed with 20 mL of water over a fritted glass filter. The powder was suspended and stirred briefly in 20 mL of acetone, collected again by filtration, and dried overnight at 80 °C under vacuum.

S.3.5. Synthesis of single crystals of *dia* - $Cu(AIm)_2$ (α form)

25 mg of copper (II) sulfate pentahydrate (0.1 mmol) was dissolved in 10 mL of water, and 1 mL of aqueous ammonia (28%) was added to form a dark blue solution. A solution of 10 mg of H**AIm** (0.11 mmol) dissolved in a mixture of 5 mL of water and 1 mL aqueous ammonia (28%) was added dropwise to the copper solution over the course of 5 minutes, with gentle stirring. The mixture was left undisturbed without stirring for another 2 hours. Examination of the resulting precipitate under an optical microscope revealed the presence of a small amount of deep-purple single crystals within a large amount of amorphous brown powder, which comprised most of the precipitate (Figure S14). These crystals were separated by hand under an optical microscope for SCXRD analysis.

S.3.6. Synthesis of single crystals of *dia* - $Cu(VIm)_2$

100 mg of copper (II) sulfate pentahydrate (0.4 mmol) was dissolved in 10 mL of water, and 1 mL of aqueous ammonia (28%) was added to form a dark blue solution. To this was added 70 mg of H**VIm** (0.74 mol), which was dissolved by sonication to yield a clear solution. After letting this mixture sit, capped, for 15 minutes, small dark blue crystals appeared along with a large amount of amorphous brown powder (Figure S14). The blue crystals were separated by hand under an optical microscope for single crystal X-ray diffraction (SCXRD) analysis.

S.3.7. Synthesis of single crystals of *dia* - $Cu(MeIm)_2$

200 mg of copper(II) sulfate pentahydrate (0.8 mmol) was dissolved in 2.5 mL of water, and 5 mL of aqueous ammonia (28 %) was added to form a dark blue solution. 300 mg of H**MeIm** (3.65 mmol) was added as a solid and was dissolved by brief sonication. This mixture was placed in an oven at 85 °C, uncapped, for 45 minutes. The resulting solid was collected from the vial by filtration and examined using an optical microscope, which revealed green single crystals along with an orange/brown impurity. Crystals were separated by hand under an optical microscope for SCXRD analysis. PXRD analysis of this mixture showed that this orange impurity was the poorly crystalline phase found during synthetic screening (Figure S14) which has not yet been identified.

S.3.8 Impact testing of copper(II)-based ZIFs

The impact sensitivities of Cu(II)-based ZIFs were tested using a standardized impact test. Approximately 25 mg of microcrystalline sample was placed between two hardened steel disks, and a 2.7 kg steel weight was dropped directly onto the top plate from a height of 2.1 m for a total impact of 50 J. The powder was immediately collected for PXRD analysis, which can be found in Section S.10.2.

S.4. Synthetic screening of copper-(II) based ZIF systems

Synthetic screening was performed to explore the phase landscape of Cu(II)-based ZIFs. Solvothermal, mechanochemical, and aqueous screening conditions and results are outlined below.

S.4.1. Screening from aqueous solutions at room temperature

Reactions were performed by dissolving a source of Cu(II), notably $CuSO_4 \cdot 5H_2O$, in water, adding aqueous ammonia (28%, ~15 mmol/mL), then adding the solid ligand (H**VIm**, H**AIm**, or H**MeIm**) with brief stirring. After 30 minutes, precipitate, if formed, was collected by centrifugation, washed with water then acetone, dried in air, and analyzed by PXRD. Reactions using the ligands H**VIm** or H**AIm** were conducted using 10 mL of water as a solvent. Reactions using H**AIm** were conducted at a ¼ scale, using 2.5 mL of water as a solvent. PXRD diffractograms for all screening reactions can be found in Section S.10.

Table S9. Reaction parameters and observed phases for the aqueous synthetic screening of Cu(**AIm**)$_2$

reaction	Cu(II) source (mmol)	H**AIm** (mmol)	$NH_{3(aq)}$ (mL)	product
1	0.25, sulfate	0.5	none	α–Cu(**AIm**)$_2$
2	0.25, sulfate	0.5	0.25	α–Cu(**AIm**)$_2$
3	0.125, sulfate	0.25	0.25	α–Cu(**AIm**)$_2$
4	0.125, sulfate	0.25	0.75	α/β-Cu(**AIm**)$_2$
5	0.125, sulfate	1	0.25	α-Cu(**AIm**)$_2$
6	0.125, sulfate	1	0.75	α/β–Cu(**AIm**)$_2$
7	0.25, tetraammine sulfate	0.5	none	α–Cu(**AIm**)$_2$
8[a]	0.25, tetraammine sulfate	0.5	none	α–Cu(**AIm**)$_2$

[a] Slurry experiment: 0.25 mL H_2O used instead of 2.5 mL.

Table S10. Reaction parameters and observed phases for the aqueous synthetic screening of Cu(**VIm**)$_2$

reaction	Cu(II) source (mmol)	H**VIm** (mmol)	$NH_{3(aq)}$ (mL)	product
9	1, sulfate	2	none	unknown 1[b]
10	1, sulfate	2	1	*dia* – Cu(**VIm**)$_2$
11	0.5, sulfate	1	1	amorphous
12	0.5, sulfate	1	3	no precipitate
13	0.5, sulfate	4	1	amorphous
14	0.5, sulfate	4	3	amorphous
15	1, tetraammine sulfate	2	none	*dia* – Cu(**VIm**)$_2$
16[a]	1, tetraammine sulfate	2	none	*dia* – Cu(**VIm**)$_2$

[a] Slurry experiment: 1 mL H_2O used instead of 10 mL. [b] Thermogravimetric analysis for copper content of this material does not match the formula Cu(**MeIm**)$_2$ (Figure S9).

Table S11. Reaction parameters and observed phases for the aqueous synthetic screening of Cu(**MeIm**)$_2$

reaction	Cu(II) source (mmol)	H**MeIm** (mmol)	$NH_{3(aq)}$ (mL)	product
17	1, sulfate	2	none	unknown 2[c]
18	1, sulfate	2	1	unknown 3[d]
19	0.5, sulfate	1	1	*dia* – Cu(**MeIm**)$_2$ + unk. 3[d]
20	0.5, sulfate	1	3	no precipitate
21	0.5, sulfate	4	1	unknown 3[d]
22[a]	0.5, sulfate	4	3	*dia* – Cu(**MeIm**)$_2$
23	1, tetraamine sulfate	2	none	unknown 3[d]
24[b]	1, tetraamine sulfate	2	none	unknown 3[d]

[a] No precipitate formed after 1 hour, so 200 μL of aqueous NaOH (25%) was added with stirring to produce the precipitate of *dia*-Cu(**MeIm**)$_2$. [b] Slurry experiment; 1 mL H_2O used instead of 10 mL. [c] Thermogravimetric analysis for copper content of this material does not match the formula Cu(**MeIm**)$_2$ (Figure S9). [d] The quality of the PXRD diffractograms of this phase is insufficient for structural characterization.

S.4.2. Solvothermal screening

The quantities and concentrations of reactants for solvothermal syntheses were chosen to match those reported in the original reports of ZIF-8[18] and ZIF-90[19] synthesis. In all cases, the metal source, notably $CuSO_4·5H_2O$ or $Cu(NO_3)_2·3H_2O$, and solid ligand (H**VIm**, H**AIm**, or H**MeIm**) were dissolved in 5 mL of solvent (DMF or NMP), sealed in a glass pressure tube, and heated for 24 hours. Precipitates, if formed, were washed with water, then acetone, and subsequently dried in air prior to analysis by PXRD. The PXRD diffractograms for all screening reactions are given in Section S.10.

Table S12. Reaction parameters and observed phases for the solvothermal screening of Cu(**AIm**)$_2$

reaction	Cu(II) source (mmol)[a]	H**AIm** (mmol)	solvent	temperature (°C)	product
25	0.223, nitrate	0.203	DMF	RT to 140 °C	amorphous
26	0.223, sulfate	0.203	DMF	RT to 140 °C	amorphous
27	0.350, nitrate	0.5	DMF	100 °C	amorphous
28	0.223, nitrate	0.203	NMP	140 °C	amorphous
29	0.350, nitrate	0.5	DMF	140 °C	amorphous
30	0.350, nitrate	0.5	NMP	140 °C	amorphous
31	0.223, nitrate	0.203	NMP	RT to 140 °C	amorphous
32	0.350, nitrate	0.5	DMF	RT to 140 °C	amorphous
33	0.350, nitrate	0.5	NMP	RT to 140 °C	amorphous

Table S13. Reaction parameters and observed phases for the solvothermal screening of Cu(**VIm**)$_2$

reaction	Cu(II) source (mmol)	H**VIm** (mmol)	solvent	temperature (°C)	product
34	0.223, nitrate	0.203	DMF	RT to 140 °C	amorphous
35	0.223, sulfate	0.203	DMF	RT to 140 °C	amorphous
36	0.350, nitrate	0.5	DMF	100 °C	amorphous
37	0.223, nitrate	0.203	NMP	140 °C	amorphous + CuO
38	0.350, nitrate	0.5	DMF	140 °C	amorphous
39	0.350, nitrate	0.5	NMP	140 °C	amorphous
40	0.223, nitrate	0.203	NMP	RT to 140 °C	amorphous
41	0.350, nitrate	0.5	DMF	RT to 140 °C	amorphous
42	0.350, nitrate	0.5	NMP	RT to 140 °C	amorphous

Table S14. Reaction parameters and observed phases for the solvothermal screening of Cu(**MeIm**)$_2$

reaction	Cu(II) source (mmol)	H**MeIm** (mmol)	solvent	temperature	product
43	0.223, nitrate	0.203	DMF	RT to 140 °C	unk. 4[a] + unk. 5[b]
44	0.223, sulfate	0.203	DMF	RT to 140 °C	unknown 5[b]
45	0.350, nitrate	0.5	DMF	100 °C	no precipitate
46	0.223, nitrate	0.203	NMP	140 °C	amorphous
47	0.350, nitrate	0.5	DMF	140 °C	amorphous
48	0.350, nitrate	0.5	NMP	140 °C	amorphous
49	0.223, nitrate	0.203	NMP	RT to 140 °C	amorphous
50	0.350, nitrate	0.5	DMF	RT to 140 °C	unknown 5[b]
51	0.350. nitrate	0.5	NMP	RT to 140 °C	amorphous

[a] This phase could not be fully characterized due to presence of an impurity of unknown 5. [b] Thermogravimetric analysis for copper content of this material does not match the formula Cu(**MeIm**)$_2$ (Figure S9).

S.4.3. Mechanochemical screening

Mechanochemical reactions were performed by ball-milling of reactants for 30 minutes at a frequency of 30 Hz, with reaction mixtures placed in 15 mL volume stainless steel jars, using a Form-Tech Scientific FTS-1000 shaker mill. For the experiments using H**MeIm** and H**VIm**, reactions were carried out at a scale of 1 mmol based on the Cu(II) source, with two stainless steel milling balls of 1.32 g weight (7 mm diameter). Reactions with H**AIm** were performed at a 0.5 mmol scale with respect to Cu(II), using a single stainless steel milling ball of 1.32 g weight (7 mm diameter). In some cases, liquid and salt additives were used (see Tables S14-16). Reaction mixtures were analyzed immediately after milling by PXRD. The PXRD diffractograms for all screening reactions are provided in Section S.10.

Table S15. Reaction parameters and observed phases for the mechanochemical screening of Cu(**AIm**)$_2$

reaction	Cu(II) source (mmol)[a]	H**AIm** (mmol)	liquid additive	ionic additive	product
52	0.5, hydroxide	1	-	-	*starting materials*
53	0.5, hydroxide	1	100 μL H_2O	-	β – Cu(**AIm**)$_2$

Table S16. Reaction parameters and observed phases for the mechanochemical screening of Cu(**VIm**)$_2$

reaction	Cu(II) source (mmol)	H**VIm** (mmol)	liquid additive	ionic additive	product
54	1, sulfate	2	-	-	unknown 6[a]
55	1, hydroxide	2	-	-	*dia* – Cu(**VIm**)$_2$
56	1, tetraammine	2	-	-	starting material
57	1, hydroxide	2	100 μL H_2O	-	*dia* – Cu(**VIm**)$_2$
58	1, tetraammine	2	100 μL H_2O	-	starting material
59	1, oxide	2	-	-	starting material
60	1, oxide	2	100 μL MeOH	-	starting material
61	1, oxide	2	100 μL DMF	-	starting material
62	1, oxide	2	100 μL MeOH	10 mg NH_4NO_3	starting material
63	1, oxide	2	100 μL DMF	10 mg NH_4NO_3	starting material

[a] This material is soluble in DMSO, which does not match the expected properties for a MOF-type material

Table S17. Reaction parameters and observed phases for the mechanochemical screening of Cu(**MeIm**)$_2$

reaction	Cu(II) source (mmol)	H**MeIm** (mmol)	liquid additive	ionic additive	product
64	1, sulfate	2	-	-	unknown 7[a]
65	1, hydroxide	2	-	-	unknown 3[b]
66	1, tetraammine	2	-	-	starting material
67	1, hydroxide	2	100 μL H_2O	-	unknown 3[b]
68	1, tetraammine	2	100 μL H_2O	-	starting material
69	1, oxide	2	-	-	unknown 3[b]
70	1, oxide	2	100 μL MeOH	-	starting material
71	1, oxide	2	100 μL DMF	-	starting material
72	1, oxide	2	100 μL MeOH	10 mg NH_4NO_3	starting material
73	1, oxide	2	100 μL DMF	10 mg NH_4NO_3	starting material

[a] This material is soluble in DMSO, which does not match the expected properties for a MOF-type material [b] The X-ray powder diffractograms for this phase were of insufficient quality for structural characterization.

S.5. Crystallographic information

S.5.1. Crystal structure determination from single crystal X-ray diffraction data

Single crystal X-ray diffraction (SCXRD) experiments were performed using a Bruker D8 Venture diffractometer equipped with a Photon 200 area detector and an *IμS* microfocus X-ray source (Bruker AXS, Cu*K*α source). Data collection was performed at room temperature (298 K) for the Cu(**MeIm**)$_2$ and α-Cu(**AIm**)$_2$ materials, and reduced temperature (150 K) for Cu(**VIm**)$_2$. Crystals were coated with paratone oil before mounting on the diffractometer. Structure solution was carried out using the SHELXTL[20] package. The parameters were refined for all data by full-matrix-least-squares refinement of F^2 using SHELXL[21] All non-hydrogen atoms were refined with anisotropic thermal parameters, and the coordinates of all hydrogen atoms were constrained to ride on their carrier atoms. The crystallographic parameters of the determined structures can be found in Table S17. The crystallographic data in CIF format for all herein determined crystal structures can be accessed using the joint CCDC/FIZ Karlsruhe online deposition service (www.ccdc.cam.ac.uk/structures/), under the deposition numbers 2176636-2176639.

S.5.2. Crystal structure determination from powder X-ray diffraction data

The crystal structure of β-Cu(**AIm**)$_2$ was determined from PXRD data, with the aid of the computational model derived through symmetry perturbation analysis (see section S.1.2). Since the perturbed and geometry-optimized structure was found in a non-standard $P112_1$ space group with the monoclinic angle γ, it had tobrought to the standard $P2_1$ crystallographic setting via PLATON ADDSYM command. The resulting structure was then subjected to Rietveld refinement[22] in TOPAS Academic V6.[23]

The first step of the refinement procedure included the unit cell parameters, pseudo-Voigt peak shape function, 6th degree Chebyshev background polynomial and instrumental zero point. Subsequently structural degrees of freedom for two Cu atoms (each with three translational degrees of freedom) and four symmetry-independent **AIm** fragments (each with three rotational and three translational degrees of freedom) were introduced. In this refinement step, distance and angle restraints were applied to the Cu-N coordination bonds and N-Cu-N coordination angles, with the idealized values taken from the DFT-optimized structure geometry. Atomic motion was refined with a single isotropic Debye-Waller factor. Details of the refinement parameters are given in Table S18, while the comparison of the calculated and experimental PXRD profile following Rietveld refinement is shown in Figure S5. Crystallographic parameters of β-Cu(**AIm**)$_2$ can be found in Table S17.

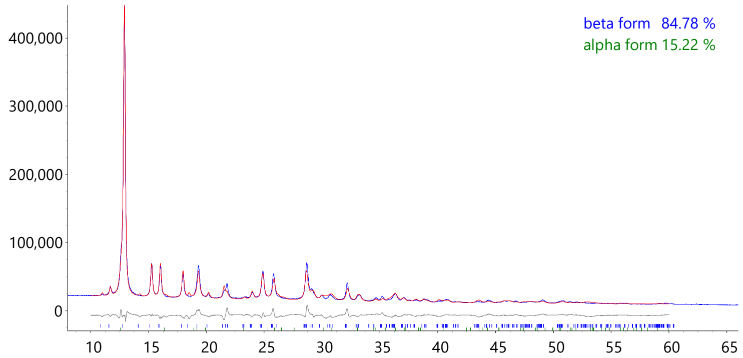

Figure S5. Rietveld refinement plot of β-Cu(**AIm**)$_2$. The experimental profile is shown in blue, calculated profile in red, and the difference curve in grey. This analysis revealed the presence of a small amount (~15 weight %) of residual α-Cu(**AIm**)$_2$ alongside the β-Cu(**AIm**)$_2$.

Table S18. Crystallographic and general parameters for the herein determined ZIF structures.

Material	Cu(**MeIm**)$_2$	Cu(**VIm**)$_2$	α-Cu(**AIm**)$_2$	β-Cu(**AIm**)$_2$
CCDC Number	2176637	2176638	2176636	2176639
Data collection type	Single crystal			Powder
Empirical formula	$C_8H_{10}CuN_4$	$C_{10}H_{10}CuN_4$	$C_{10}H_6CuN_4$	$C_{10}H_6CuN_4$
Formula weight	225.74	249.76	245.73	245.73
Temperature (K)	298(2)	150(2)	298(2)	298(2)
Crystal system	orthorhombic	tetragonal	tetragonal	Monoclinic
Space group	Fdd2	$I4_1$	$I4_1$	$P2_1$
a (Å)	8.0097(5)	7.4615(10)	7.7149(4)	7.9267(16)
b (Å)	15.3152(9)	7.4615(10)	7.7149(4)	16.288(2)
c (Å)	16.6204(9)	18.614(3)	17.8648(12)	7.9217(13)
α (°)	90	90	90	90
β (°)	90	90	90	104.875(8)
γ (°)	90	90	90	90
Volume (Å^3)	2038.8(2)	1036.3(3)	1063.31(13)	988.5(3)
Z	8	4	4	4
ρ_{calc} (g/cm^3)	1.471	1.601	1.535	1.651
μ (mm^{-1})	2.703	2.728	2.658	-
F(000)	920.0	508.0	492.0	492.0
2Θ range for data collection (°)	13.566 to 144.628	16.804 to 144.888	12.498 to 144.598	4 to 68.5
X-ray radiation type and wavelength / Å	Cu$K\alpha$, 1.5418 Å	Cu$K\alpha$, 1.5418 Å	Cu$K\alpha$, 1.5418 Å	Cu$K\alpha$, 1.5418 Å
Index ranges	$-9 \le h \le 9$, $-18 \le k \le 18$, $-20 \le l \le 20$	$-8 \le h \le 9$, $-9 \le k \le 9$, $-22 \le l \le 22$	$-9 \le h \le 8$, $-9 \le k \le 5$, $-17 \le l \le 21$	-
Reflections collected	5894	7731	4371	-
Independent reflections	1000 [R_{int} = 0.0556, R_{sigma} = 0.0376]	1023 [R_{int} = 0.0388, R_{sigma} = 0.0241]	969 [R_{int} = 0.1533, R_{sigma} = 0.1042]	-
Data/restraints/constraints/parameters	1000/1/-/62	1023/1/-/69	969/55/-/70	-/20/96/55
Goodness-of-fit on F^2	0.936	1.009	0.956	10.624
Final R indexes [I>=2σ (I)]	R_1 = 0.0335, wR$_2$ = 0.1060	R_1 = 0.0208, wR_2 = 0.0574	R_1 = 0.0539, wR_2 = 0.1129	-
Final R indexes [all data]	R_1 = 0.0348, wR$_2$ = 0.1076	R_1 = 0.0214, wR_2 = 0.0582	R_1 = 0.1186, wR_2 = 0.1544	R_p = 0.052, Rwp = 0.072
Largest diff. peak/hole (e Å^{-3})	0.31/-0.35	0.19/-0.22	0.32/-0.36	-

S.6. FTIR-ATR spectra of copper(II)-based ZIFs

FTIR-ATR spectra were measured using a Bruker Vertex 70 FTIR spectrometer equipped with the Bruker Platinum ATR accessory.

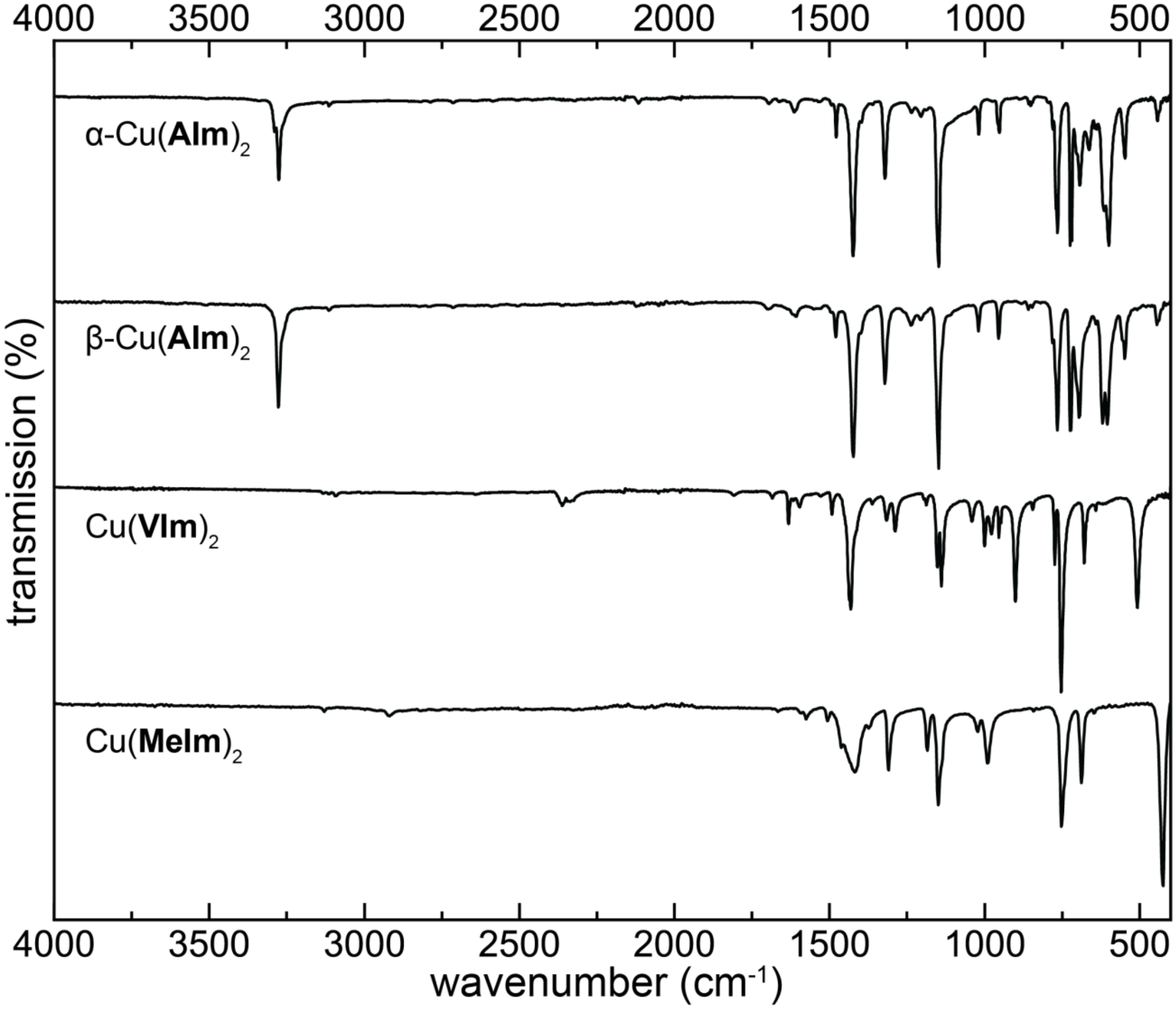

Figure S6. Comparison of infrared spectra for microcrystalline Cu(II)-based ZIFs.

S.7. ^{1}H NMR spectra of acid-digested $Cu(AIm)_2$ ZIFs

^{1}H NMR Spectra were collected on a Varian Inova 500 MHz spectrometer. The ZIF samples were dissolved in concentrated DCl (37 % in D_2O, Sigma Aldrich) and then diluted further with D_2O (Sigma Aldrich) before measurement.

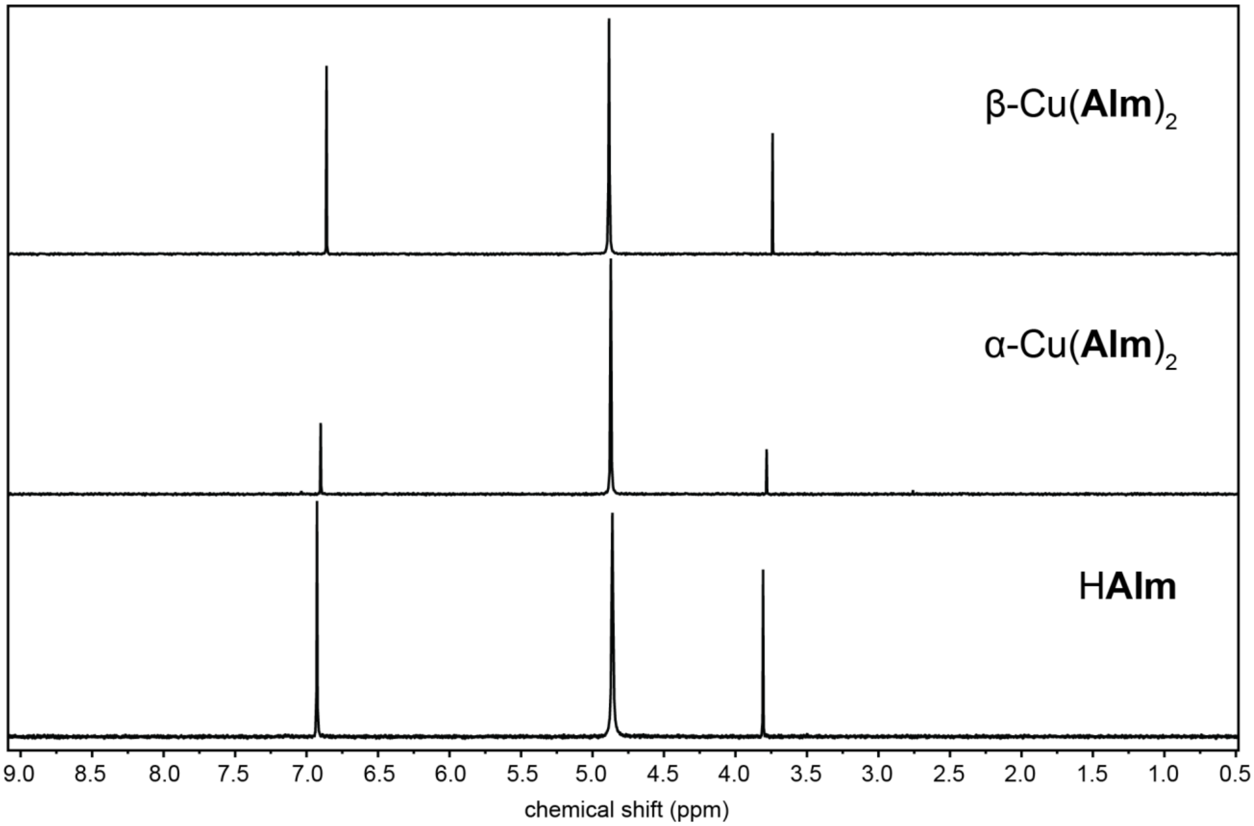

Figure S7. ^{1}H NMR spectra of Cu(**AIm**)$_2$ ZIFs digested in dilute DCl in D_2O. Identical ^{1}H NMR spectra for α-Cu(**AIm**)$_2$, β-Cu(**AIm**)$_2$, and H**AIm** show that the **AIm** linker is chemically unchanged after milling α-Cu(**AIm**)$_2$ to produce β-Cu(**AIm**)$_2$.

S.8. TGA/DSC analysis of copper(II)-based ZIFs

Thermogravimetric analysis (TGA) and differential scanning calorimetry (DSC) were performed simulatenously using a Mettler-Toledo TGA/DSC 1 instrument. The sample (3 – 10 mg) was loaded into a 70 µL alumina pan and heated from 30 °C to 800 °C under a stream of air (65 mL min^{-1}).

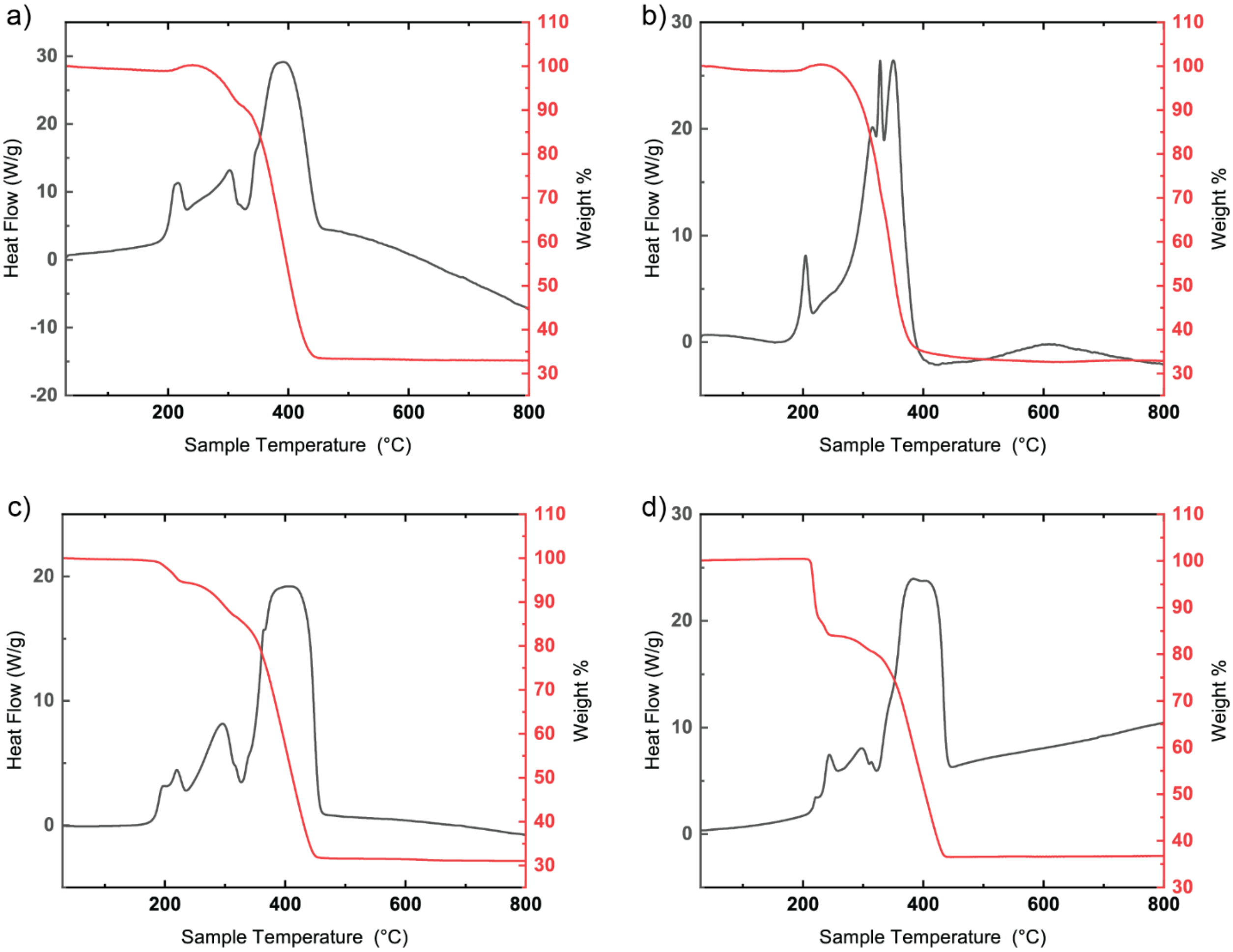

Figure S8. Thermogravimetric and differential scanning calorimetry analysis of Cu(**VIm**)$_2$ (theoretical residue: **31.8%** experimental: **31.3%**), α-Cu(**AIm**)$_2$ (theoretical residue: **32.4%** experimental: **33.2%**), β-Cu(**AIm**)$_2$ (theoretical residue: **32.4%** experimental: **32.5%**) and Cu(**MeIm**)$_2$ (theoretical residue: **35.2%** experimental: **36.6%**).

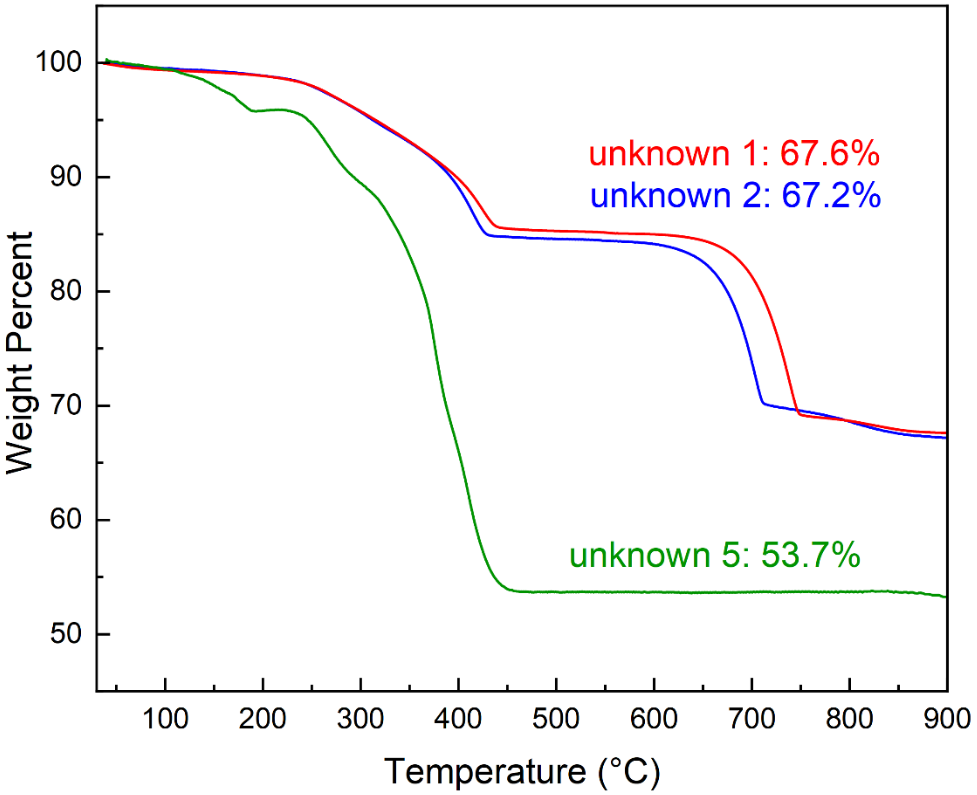

Figure S9. Comparison of TGA thermograms for the unknown phases encountered during synthetic screening for copper(II)-based ZIFs: unknown 1 (red, theoretical residue for Cu(**VIm**)$_2$: **31.8%**, experimental: **67.6%**); unknown 2 (blue, theoretical residue for Cu(**MeIm**)$_2$: **35.2%**, experimental: **67.2%**); and unknown 5 (green, theoretical residue for Cu(**MeIm**)$_2$: **35.2%**, experimental: **53.7%**).

S.10. N_2 gas sorption isotherms for copper(II)-based ZIFs

Gas sorption analysis was performed using the Autosorb-IQ gas sorption analyzer from Quantachrome Instruments, at a temperature of 77 K. Before measurement, samples were activated by exposure to 100 °C under high vacuum for 12 hours.

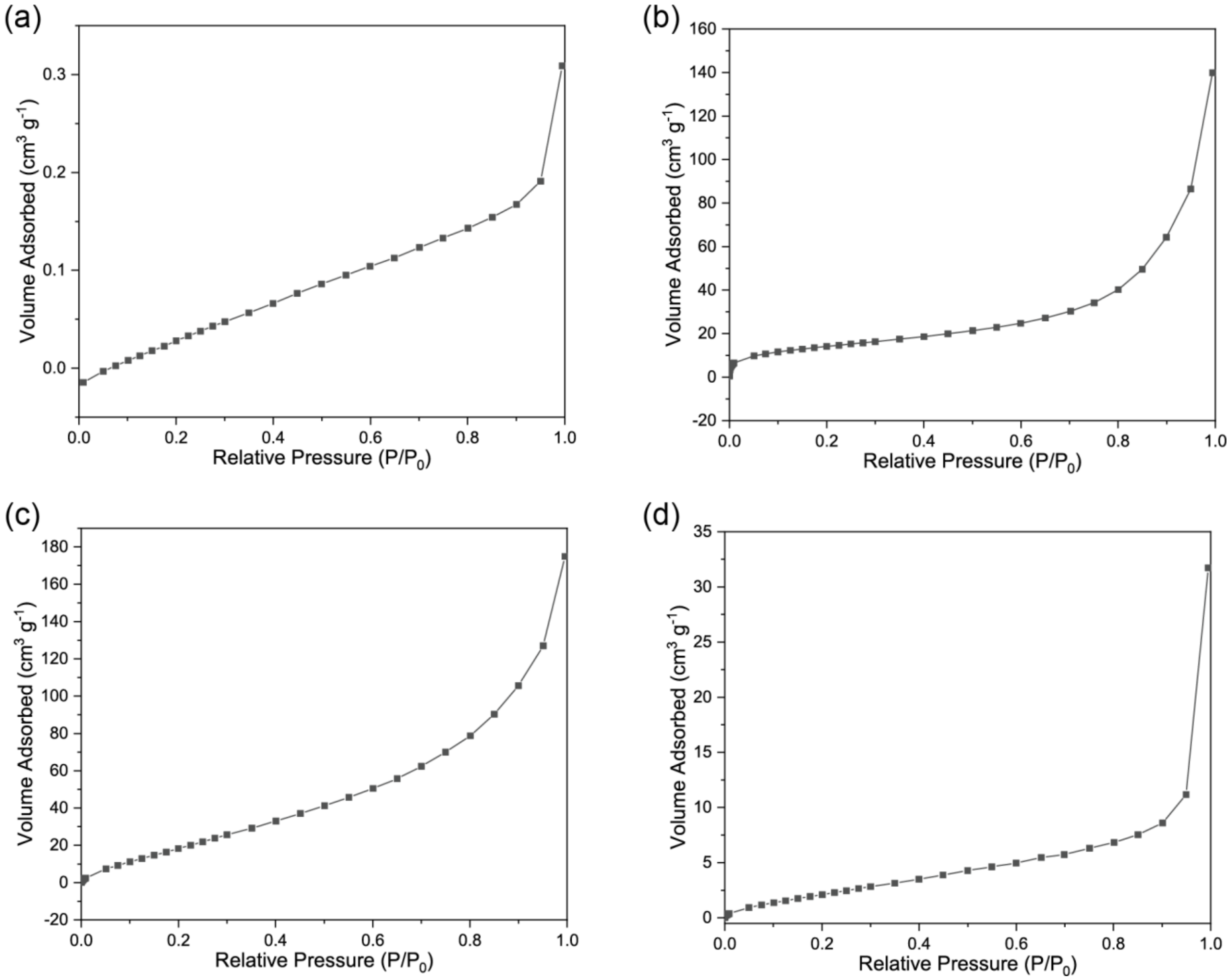

Figure S10. Nitrogen BET sorption isotherms of (a) α-Cu(**AIm**)$_2$, (b) β-Cu(**AIm**)$_2$, (c) Cu(**VIm**)$_2$, and (d) Cu(**MeIm**)$_2$.

Table S19. Multipoint BET Surface Areas of Cu(II)-based ZIF materials

Material	Multipoint BET Surface Area
α-Cu(**AIm**)$_2$	0.4 m^2 g^{-1}
β-Cu(**AIm**)$_2$	52 m^2 g^{-1}
Cu(**VIm**)$_2$	102 m^2 g^{-1}
Cu(**MeIm**)$_2$	11 m^2 g^{-1}

S.11. PXRD analysis of copper(II)-based ZIFs

Powder X-ray diffraction (PXRD) experiments were performed using a Bruker D2 Phaser diffractometer equipped with a nickel-filtered CuK_{α} X-ray radiation source and a Lynxeye 1D detector.

S.11.1. PXRD analysis of microcrystalline copper(II)-based-ZIFs

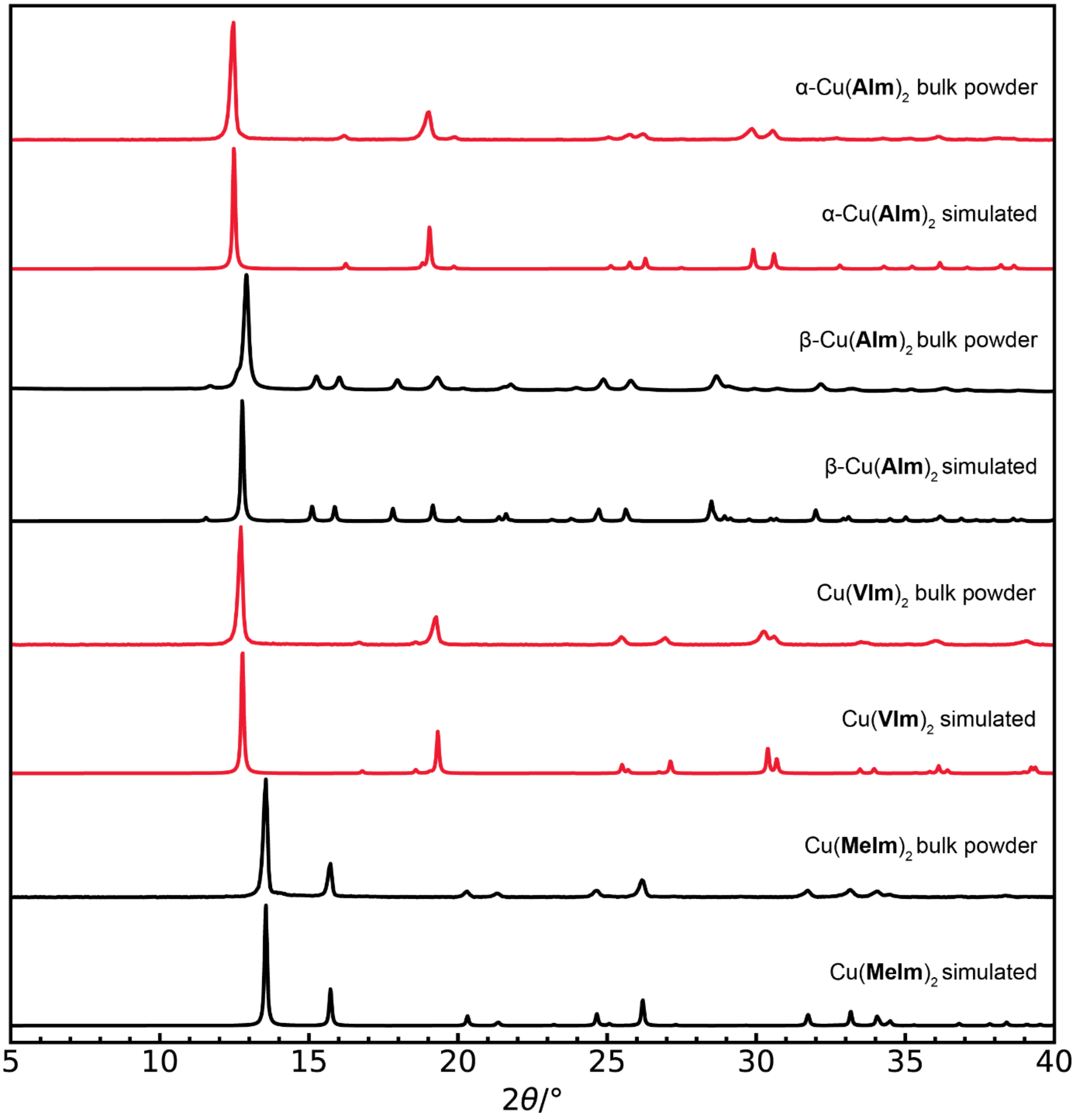

Figure S11. Powder X-ray diffractograms of microcrystalline Cu(II)-based ZIFs compared to simulated powder X-ray diffractograms based on the crystal structures herein determined by single crystal X-ray diffraction.

S.11.2. PXRD analysis of impact tests for copper(II)-based ZIFs

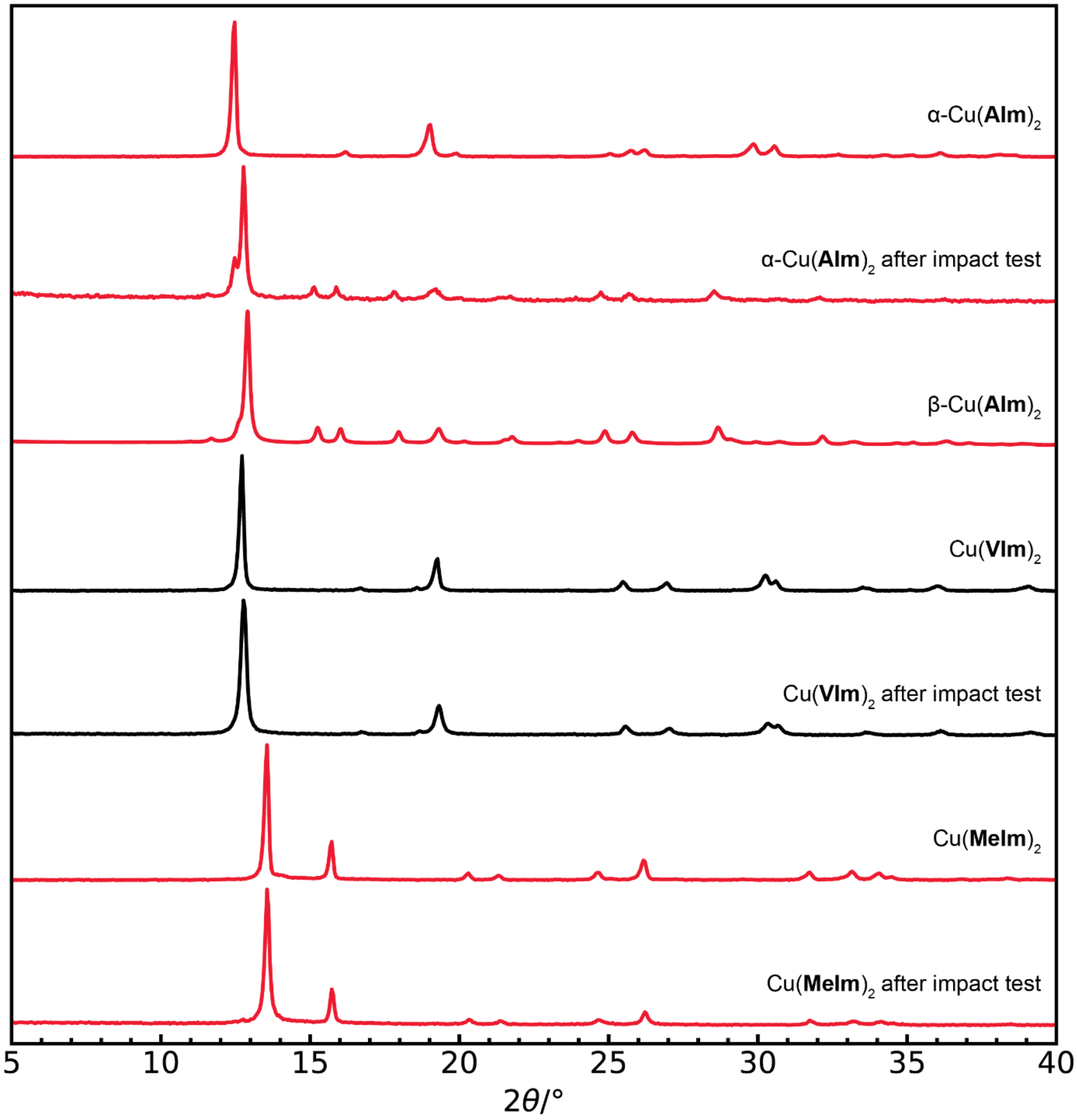

Figure S12. Powder X-ray diffractograms for samples of Cu(II)-based ZIFs after impact testing, based on a hammer drop test in which a 2.7 kg steel weight was dropped from a height of 2.1 m onto ca. 25 mg of sample, representing a 50 J impact.

S.11.3. PXRD analysis of milling stability tests of copper(II)-based? ZIFs

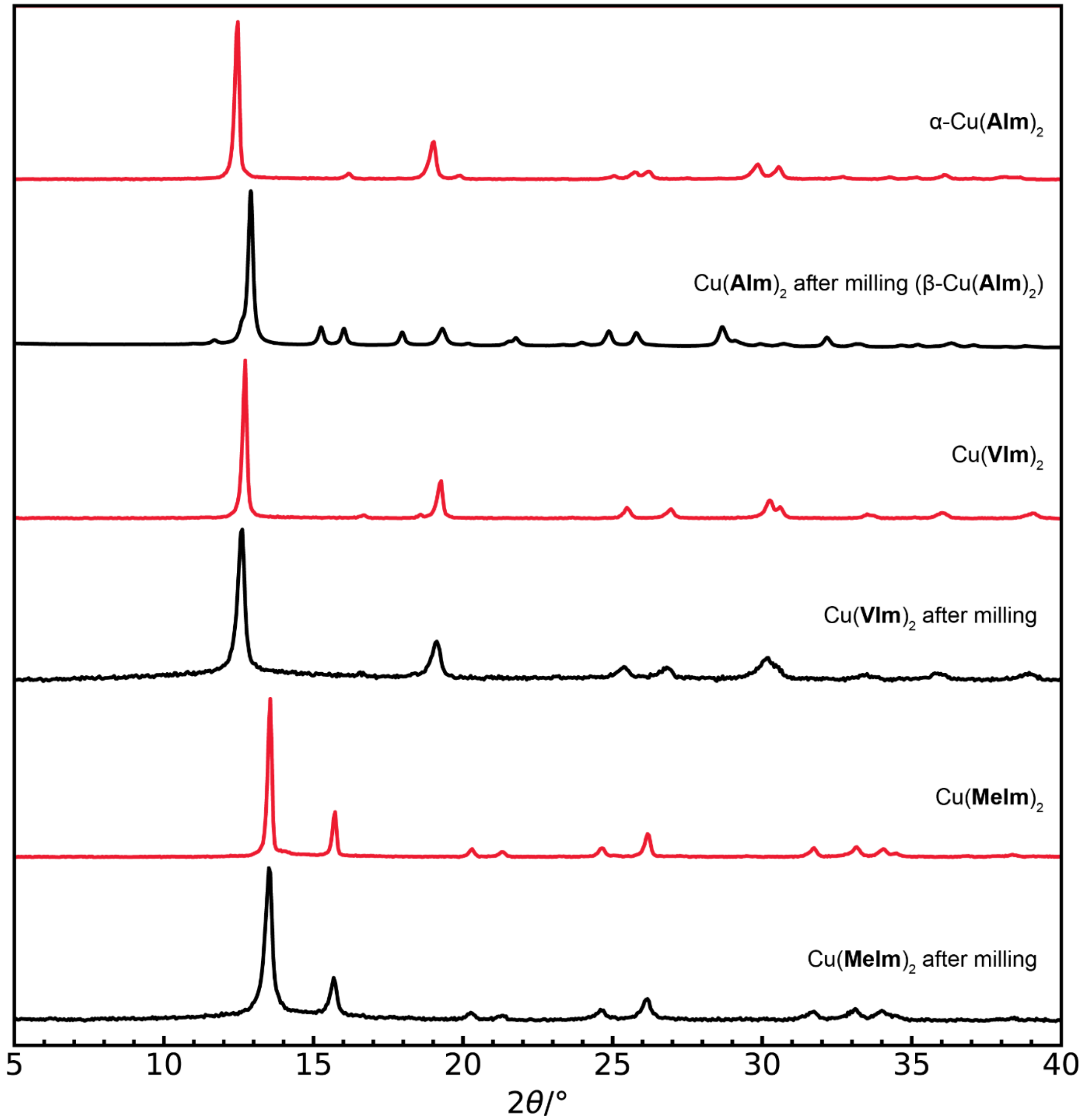

Figure S13. Comparison of powder X-ray diffractograms for samples of herein prepared Cu(II) ZIFs after ball-milling. In all cases, 60 mg of a Cu(II)-based ZIF was milled with 30 µL of methanol over 20 minutes, at a frequency of 25 Hz, using a 15 mL volume zirconia jar equipped with one 3.2 g (diameter = 11 mm) zirconia milling ball. The α-Cu(**AIm**)$_2$ material was found to converts β-Cu(**AIm**)$_2$, while no notable changes were observed for Cu(**VIm**)$_2$ and Cu(**MeIm**)$_2$.

S.11.4. PXRD analysis of product mixtures for attempted single crystal syntheses of copper(II) ZIFs

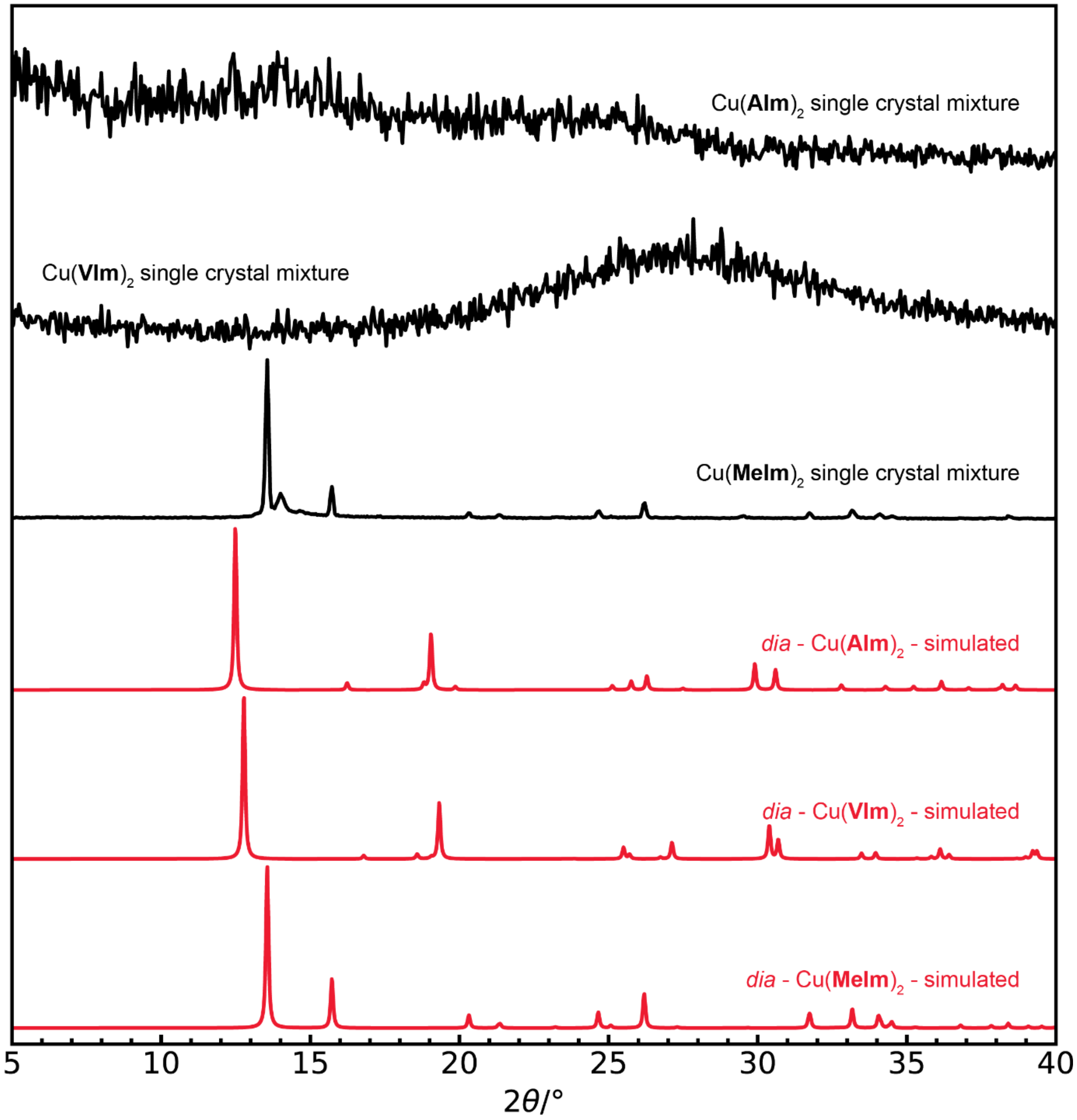

Figure S14. Comparison of powder X-ray diffractograms for product mixtures obtained during the synthesis of single crystals of Cu(II)-based ZIFs. Single crystals for *dia*-Cu(**AIm**)2 and *dia*-Cu(**VIm**)2 occur so infrequently among the bulk amorphous powder that they appear not detected by PXRD. Single crystals of *dia*-Cu(**AIm**)2 are abundant, but appear alongside an orange impurity whose PXRD pattern matches *unknown 3* described during the synthetic screenings.

S.11.5. PXRD analysis for reactions of aqueous screening for the Cu(AIm)2 system

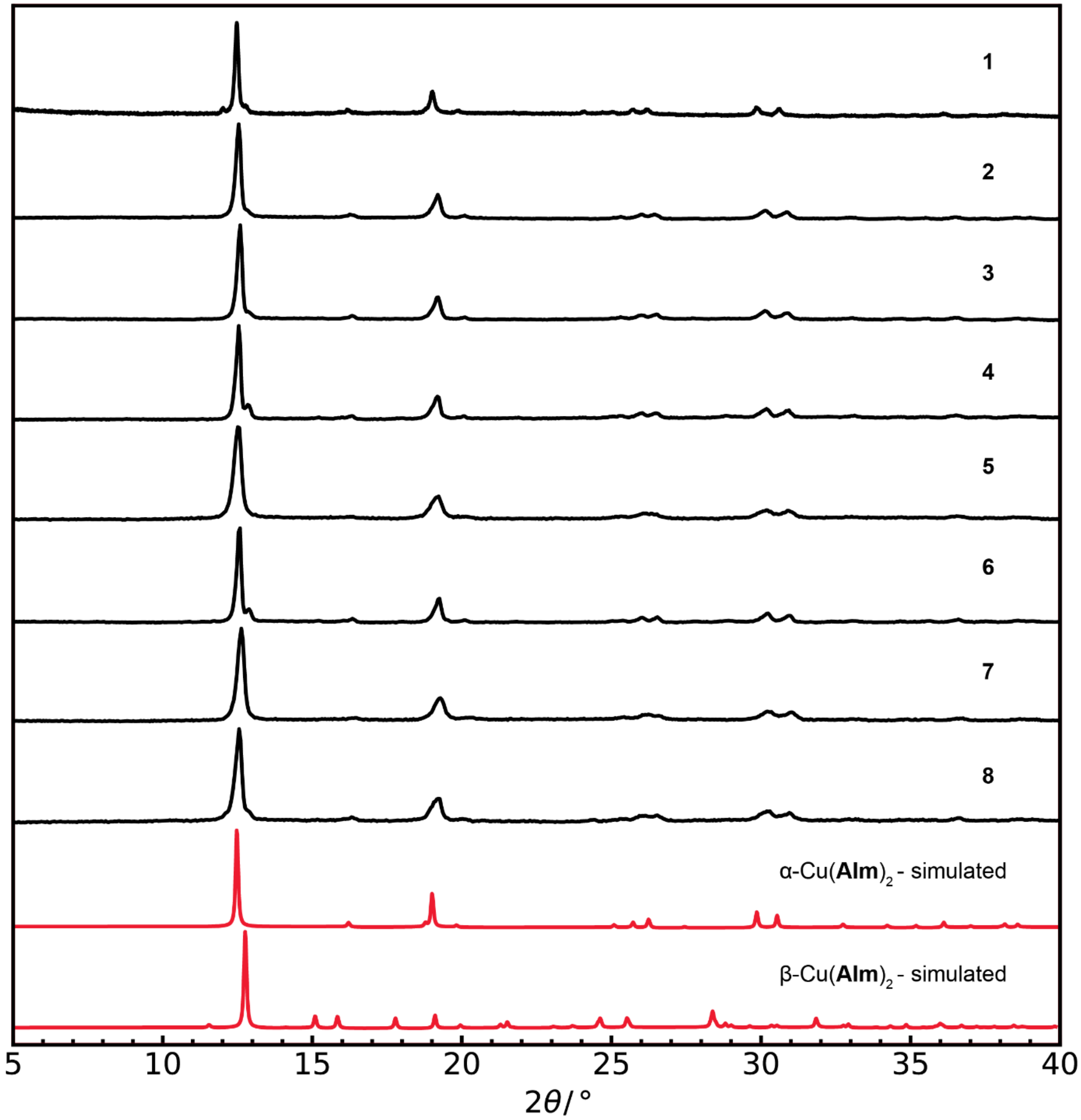

Figure S15. Powder X-ray diffractograms for the synthetic screening of the Cu(**AIm**)$_2$ system in water. Shown are products (black) and simulated *dia*-Cu(**AIm**)$_2$ structures (red). Labels correspond to reactions numbers in the synthetic screening tables (SI section S.4).

S.11.6. PXRD analysis for reactions of aqueous screening for the Cu(VIm)$_2$ system

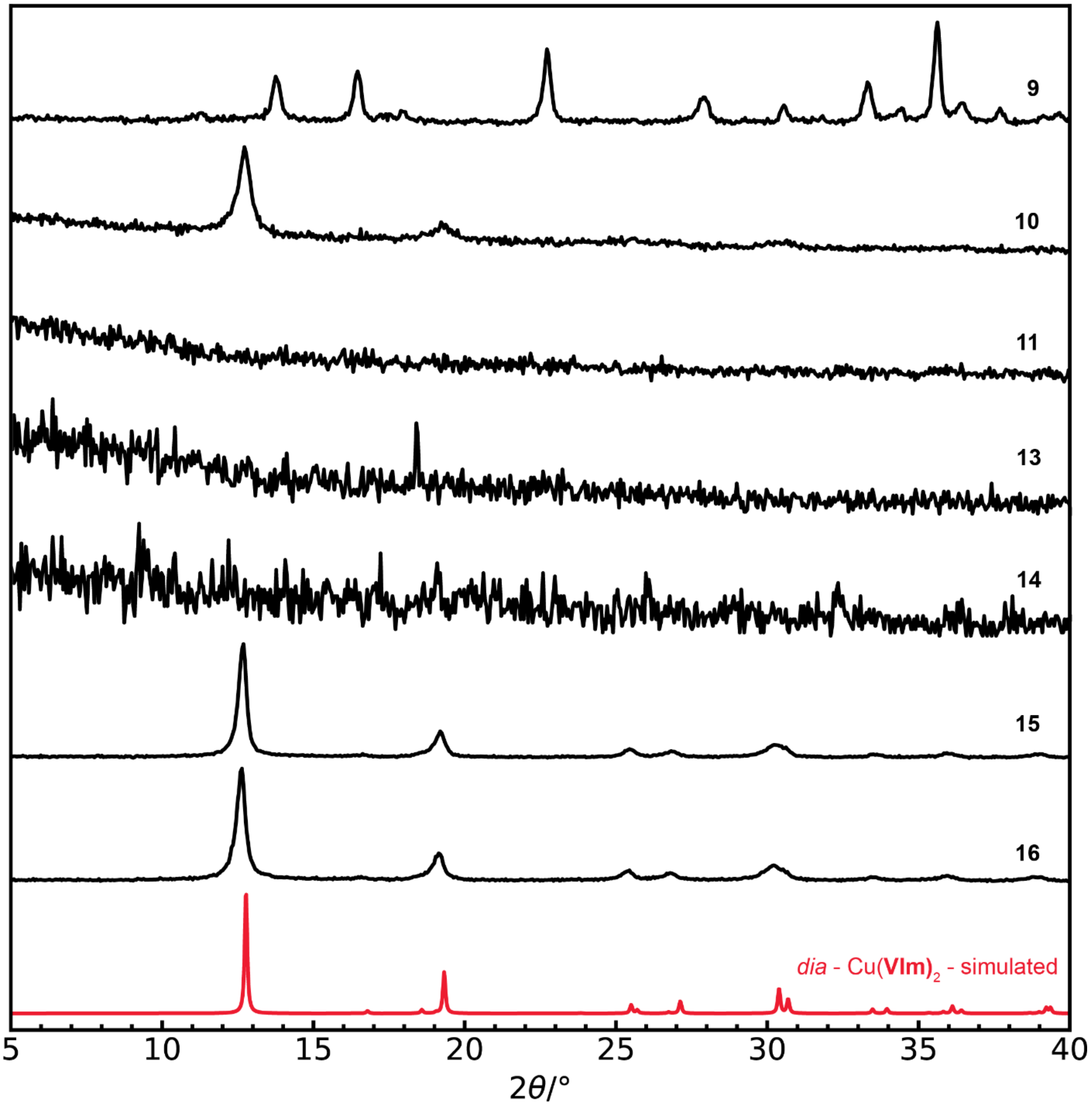

Figure S16. Powder X-ray diffractograms for the synthetic screening of the Cu(**VIm**)$_2$ system in water. Shown are products (black) and simulated *dia*-Cu(**VIm**)$_2$ (red). Labels correspond to reactions numbers in the synthetic screening tables (SI section S.4).

S.11.7. PXRD analysis for the reactions of aqueous screening for the Cu(MeIm)$_2$ system

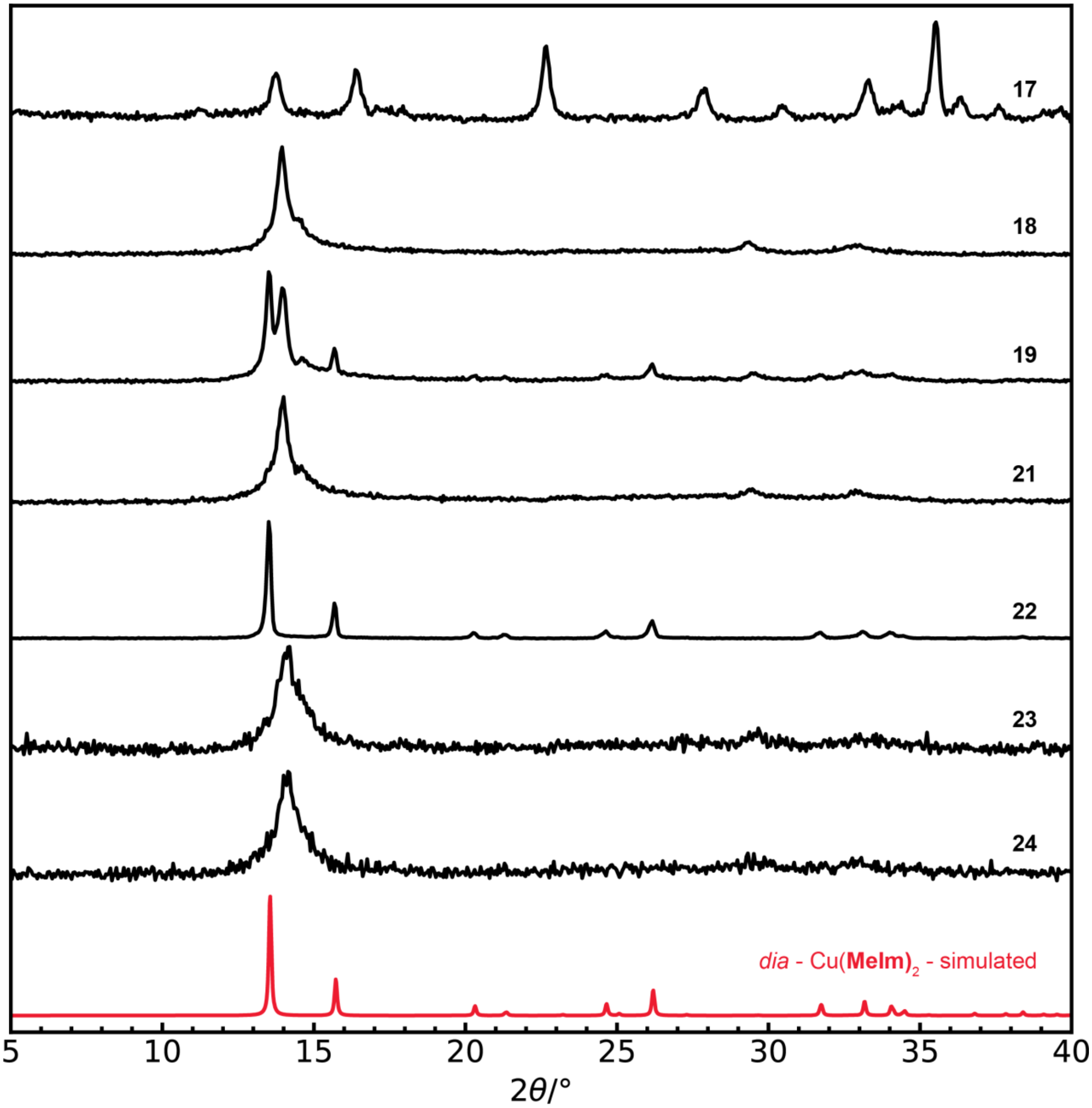

Figure S17. Powder X-ray diffractograms for the synthetic screening of the Cu(**MeIm**)$_2$ system in water. Shown are products (black) and simulated *dia*-Cu(**MeIm**)$_2$ (red). Labels correspond to reactions numbers in the synthetic screening tables (SI section S.4).

S.11.8. PXRD analysis for reactions of solvothermal screening for the Cu(AIm)$_2$ system

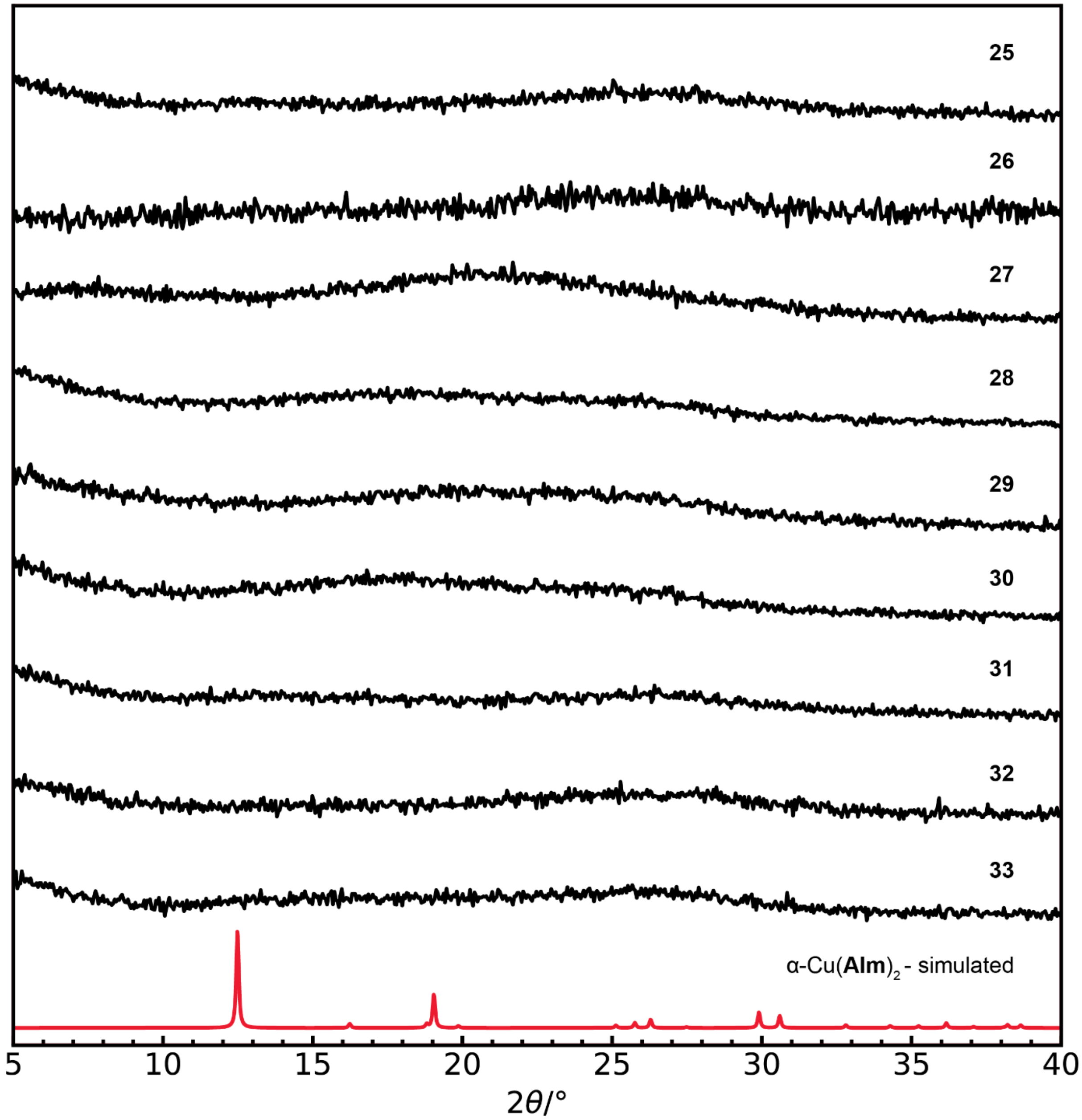

Figure S18. Powder X-ray diffractograms for the solvothermal screening of the Cu(**AIm**)$_2$ system. Shown are products (black) and simulated α-Cu(**AIm**)$_2$ (red). Labels correspond to reactions numbers in the synthetic screening tables (SI section S.4).

S.11.9. PXRD analysis for reactions of solvothermal screening for the $Cu(VIm)_2$ system

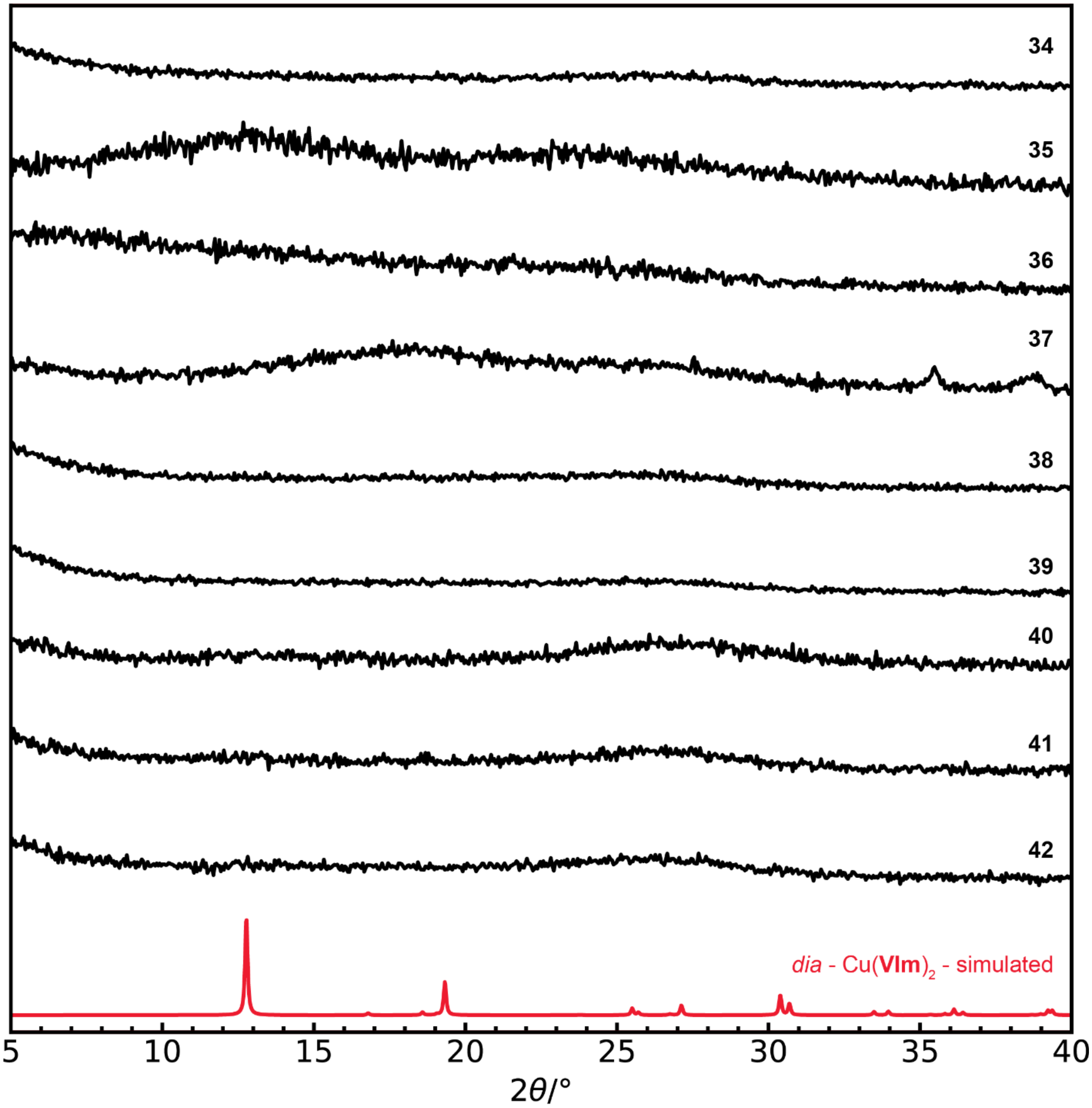

Figure S19. Powder X-ray diffractograms for the solvothermal screening of the Cu(**VIm**)$_2$ system. Shown are products (black) and simulated *dia*-Cu(**VIm**)$_2$ (red). Labels correspond to reactions numbers in the synthetic screening tables (SI section S.4).

S.11.10. PXRD analysis for the reactions of solvothermal screening for the Cu(MeIm)$_2$ system

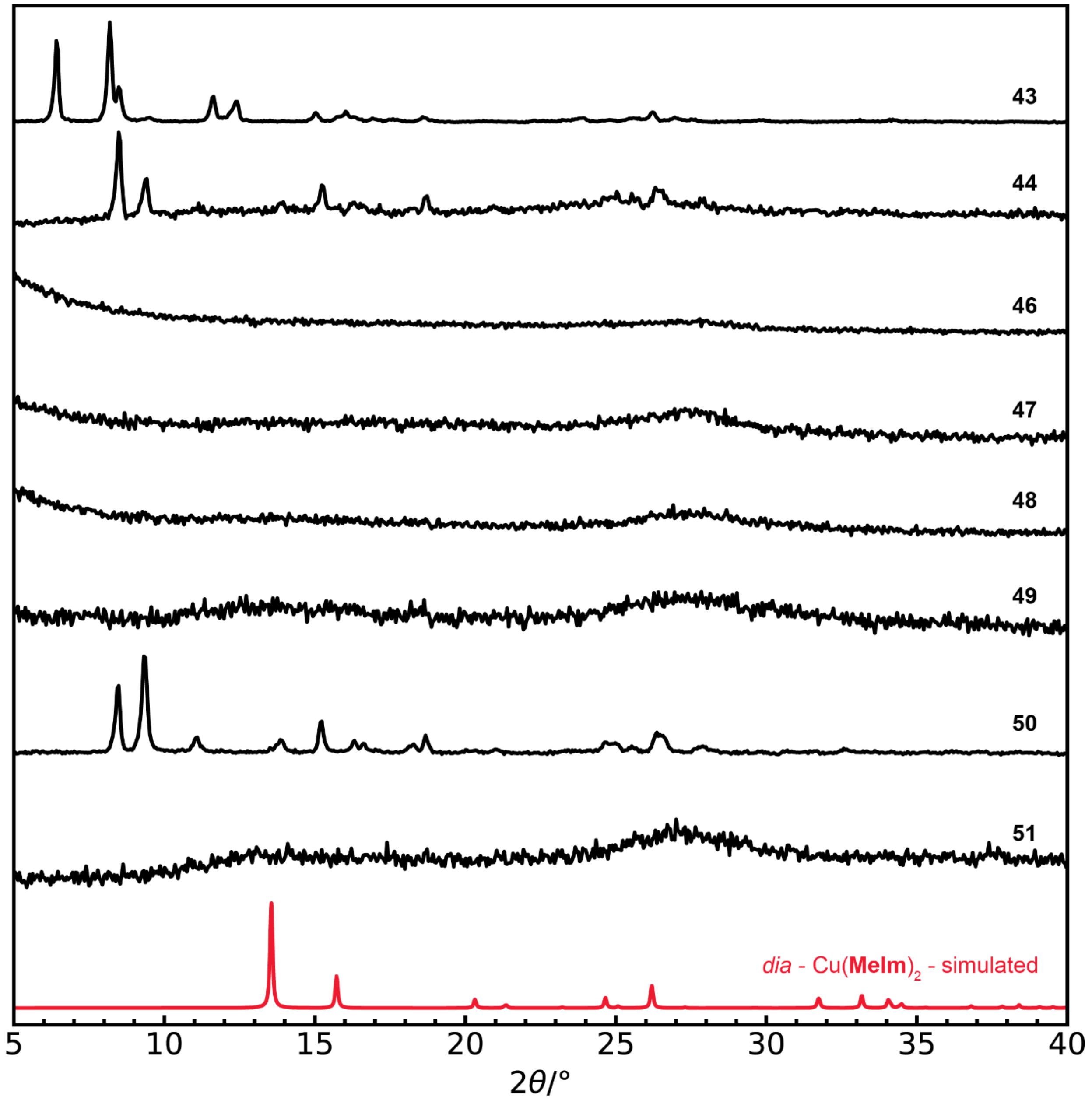

Figure S20. Powder X-ray diffractograms for the solvothermal screening of the Cu(**MeIm**)$_2$ system. Shown are products (black) and simulated *dia*-Cu(**MeIm**)$_2$ (red). Labels correspond to reactions numbers in the synthetic screening tables (SI section S.4).

S.11.11. PXRD analysis for the reactions of mechanochemical screening for the Cu(AIm)$_2$ system

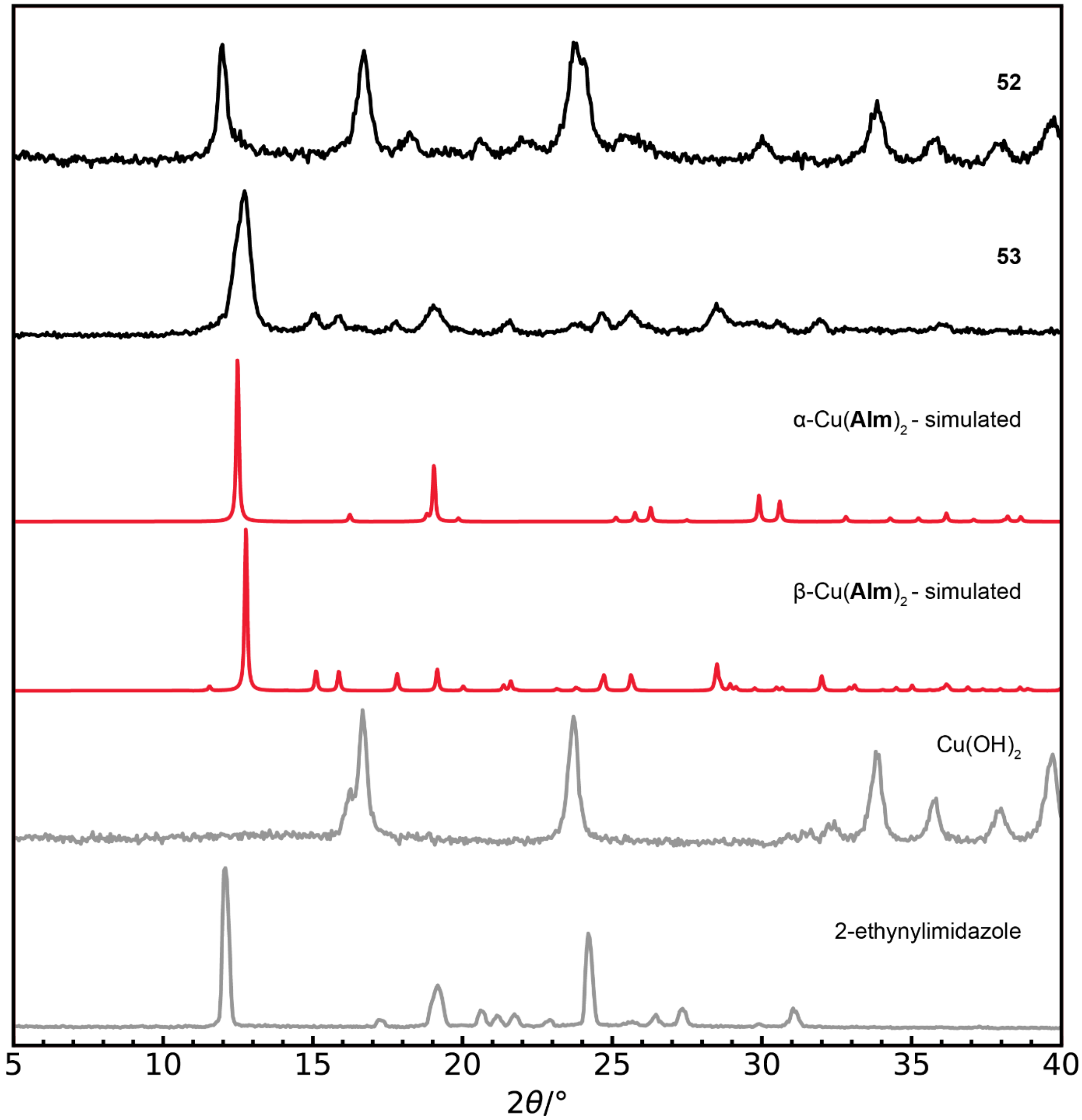

Figure S21. Powder X-ray diffractograms for mechanochemical synthesis screening of the Cu(**AIm**)$_2$ system. Shown are milling products (black), simulated *dia*-Cu(**AIm**)$_2$ structures (red), and starting materials (grey). Labels correspond to reactions numbers in the synthetic screening tables (SI section S.4).

S.11.12. PXRD analysis for the reactions of mechanochemical screening for the Cu(VIm)2 system

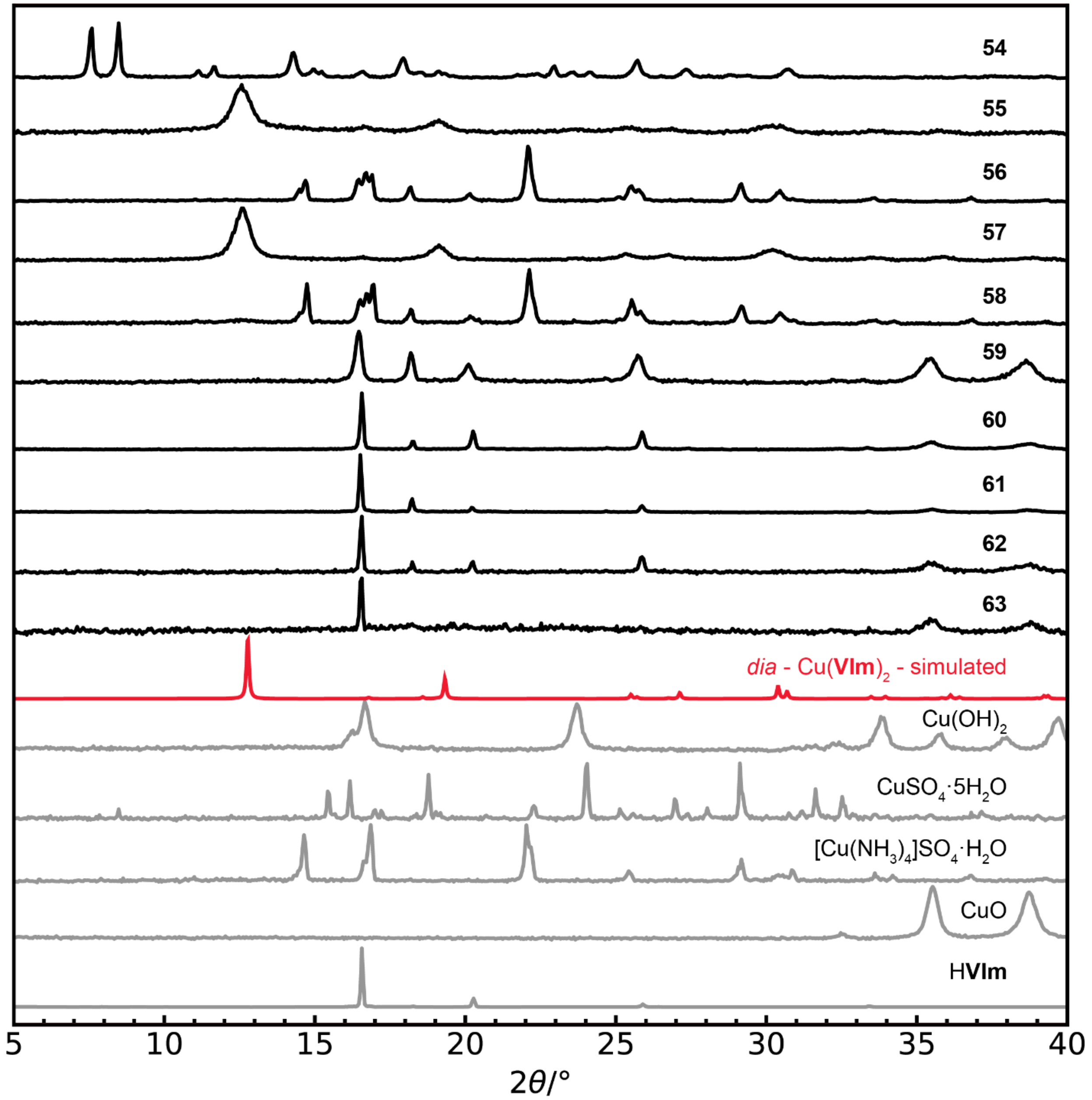

Figure S22. Powder X-ray diffractograms for mechanochemical synthesis screening of the Cu(**VIm**)$_2$ system. Shown are milling products (black), simulated *dia*-Cu(**VIm**)$_2$ (red), and starting materials (grey). Labels correspond to reactions numbers in the synthetic screening tables (SI section S.4).

S.11.13. PXRD analysis for the reactions of mechanochemical screening for the Cu(MeIm)2 system

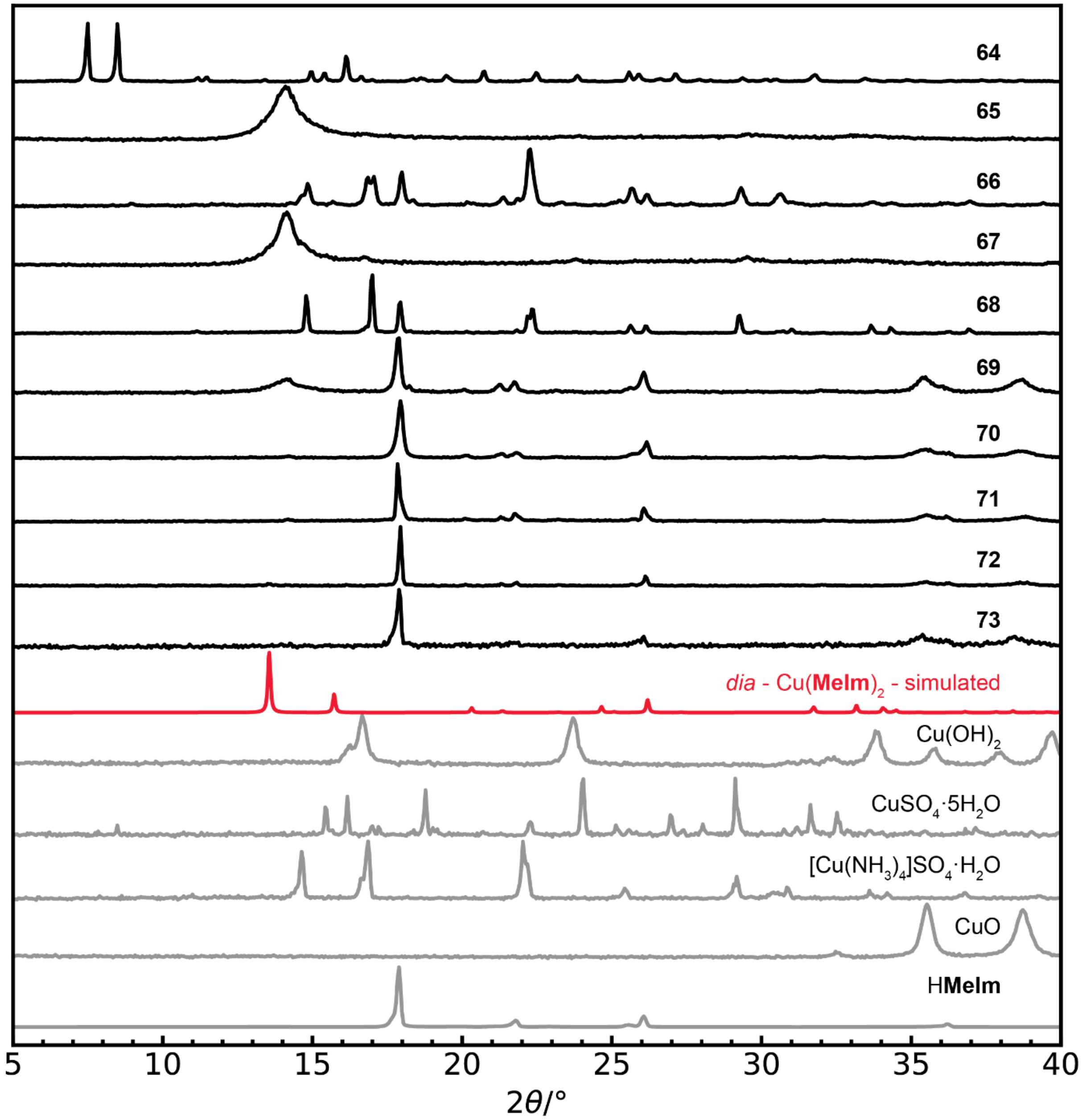

Figure S23. Powder X-ray diffractograms for mechanochemical screening of the Cu(**MeIm**)$_2$ system. Shown are milling products (black), simulated *dia*-Cu(**MeIm**)$_2$ (red), and starting materials (grey). Labels correspond to reactions numbers in the synthetic screening tables (SI section S.4).

S.12. SEM images of microcrystalline copper(II)-based ZIF powders

SEM images were collected using a FEI Quanta 450 environmental Scanning Electron Microscope. Samples were sputter coated with a 4 nm layer of Pt before imaging.

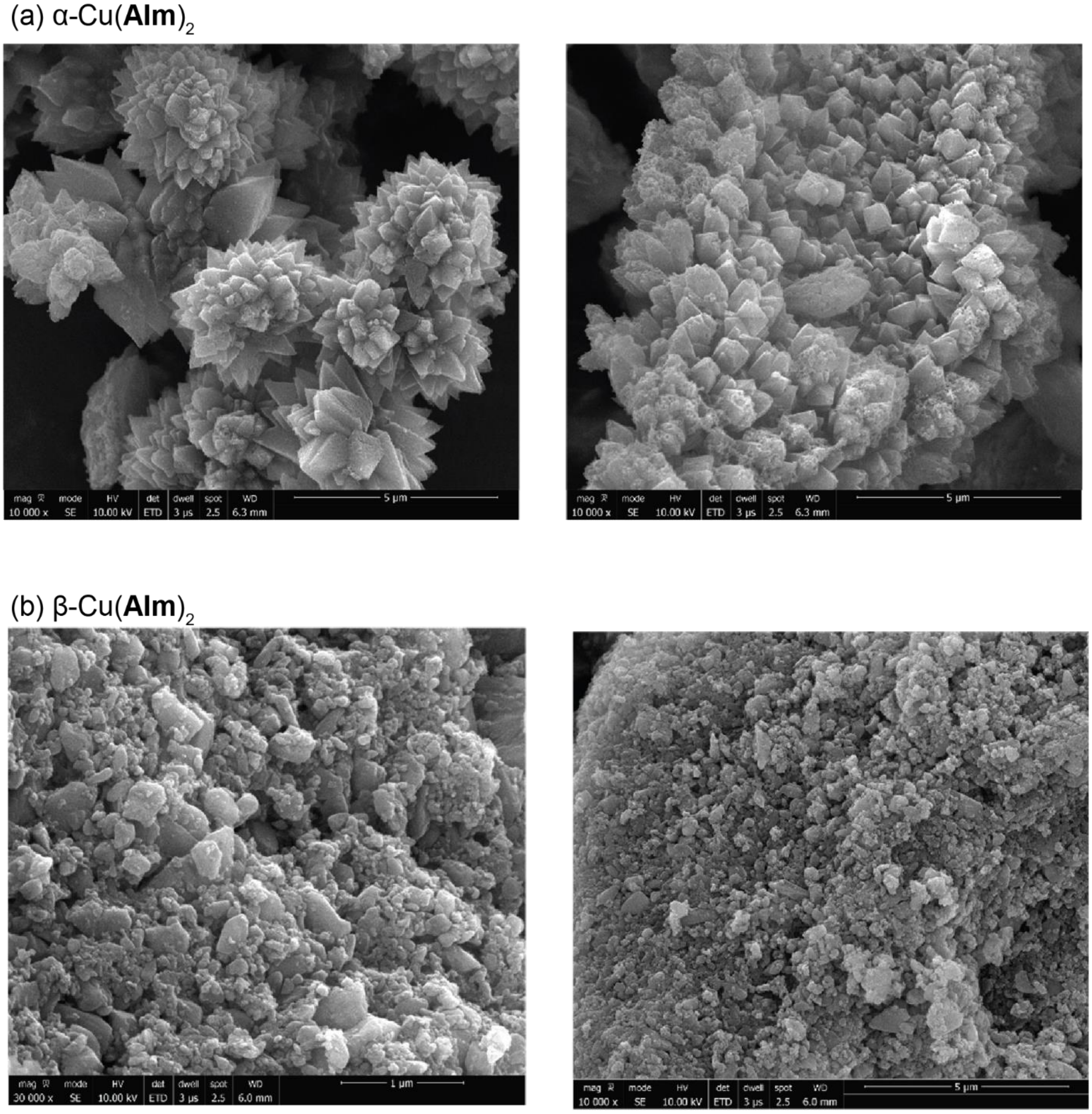

Figure S24. SEM images for samples of: (a) α-Cu(**AIm**)$_2$ and (b) β-Cu(**AIm**)$_2$.

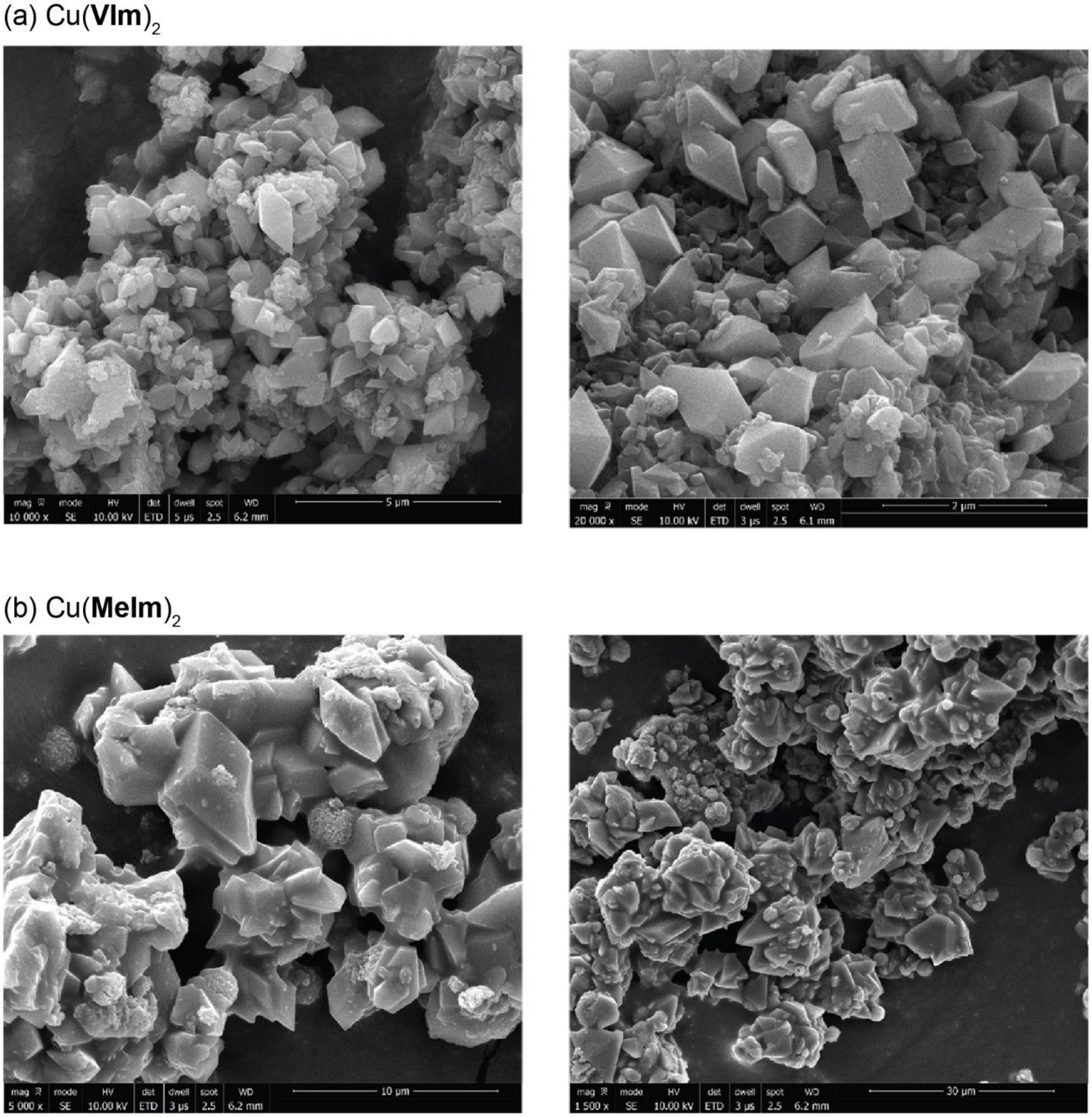

Figure S25. SEM images for samples of: (a) Cu(**VIm**)$_2$ and (b) Cu(**MeIm**)$_2$.

S.13. References

1. Pickard, C. J.; Needs, R. *Ab initio* random structure searching. *J. Phys. Condens. Matter.* **2011**, *23*, 05320.
2. Darby, J. P.; Arhangelskis, M.; Katsenis, A. D.; Marrett, J. M.; Friščić, T; Morris, A. J. Ab initio prediction of metal-organic framework structures. *Chem. Mater.* **2020**, *32,* 5835-5844.
3. Clark, S. J.; Segall, M. D.; Pickard, C. J.; Hasnip, P. J.; Probert, M. I. J.; Refson, K.; Payne, M.C. First principles methods using CASTEP. *Z. Kristallogr.* **2005**, *220*, 567-570.
4. Perdew, J. P.; Burke, K.; Ernzerhof, M. Generalized Gradient Approximation Made Simple. *Phys. Rev. Lett.* **1996**, *77*, 3865-3868.
5. Grimme, S. Semiempirical GGA-type density functional constructed with a long-range dispersion correction. *J. Comput. Chem.* **2006**, *27*, 1787–1799.
6. Chisholm, J. A.; Motherwell, S. COMPACK: a program for identifying crystal structure similarity using distances. *J. Appl. Cryst.* **2005**, *38*, 228-231.
7. Groom, C. R.; Bruno, I. J.; Lightfoot, M. P.; Ward, S. C. The Cambridge Structural Database. *Acta Crystallogr.* **2016**, *B72*, 171-179.
8. Arhangelskis, M.; Katsenis, A. D.; Novendra, N.; Akimbekov, Z; Gandrath, D.; Marrett, J. M.; Ayoub, G.; Morris, A. J.; Farha, O. K.; Friščić, T.; Navrotsky, A. Theoretical Prediction and Experimental Evaluation of Topological Landscape and Thermodynamic Stability of a Fluorinated Zeolitic Imidazolate Framework. *Chem. Mater.* **2019**, *31*, 3777–3783.
9. Tkatchenko, A.; DiStasio, R. A.; Car, R.; Scheffler, M. Accurate and Efficient Method for Many-Body van der Waals Interactions. *Phys. Rev. Lett.* **2012**, *103*, 236402.
10. Ambrosetti, A.; Reilly, A. M.; DiStasio, A.; Tkatchenko, A. Long-range correlation energy calculated from coupled atomic response functions. *J. Chem. Phys.* **2014**, *140*, 18A508.
11. Reilly A. M.; Tkatchenko, A. van der Waals dispersion interactions in molecular materials: beyond pairwise additivity. *Chem. Sci.* **2015**, *6*, 3289–3301.
12. Spek, A. L. Structure validation in chemical crystallography. *Acta Crystallogr.* **2009**, *D65*, 148-155.
13. Yang, L.; Powell, D. R.; Houser, R. P. Structural variation in copper(I) complexes with pyridylmethylamide ligands: structural analysis with a new four-coordinate geometry index, τ_4. *Dalton Trans.* **2007**, 955–964 .
14. Blatov, V. A.; Shevchenko, A. P.; Proserpio, D. M. Applied topological analysis of crystal structures with the program package ToposPro. *Crystal Growth & Design.* **2014**, *14*, 3576-3586.
15. Sun, Q.; He, H.; Gao, W.; Aguila, B.; Wojtas, L.; Dai, Z.; Li, J.; Chen, Y.; Xiao, F.; Ma, S. Imparting amphiphobicity on single-crystalline porous materials. *Nat. Commun.* **2016**, *7*, 13300.
16. Dirat, O.; Clipson, A.; Elliott, J. M.; Garrett, S.; Jones, A. B.; Reader, M.; Shaw, D. Synthesis of 4-(2-alkyl-5-methyl-2*H*-pyrazol-3-yl)-piperidines. *Tetrahedron Lett.* **2006**, *47*, 1729–1731.
17. Mazzi, F. The crystal structure of cupric tetrammine sulfate monohydrate, $Cu(NH_3)_4SO_4.H_2O$. *Acta Crystallogr.* **1955**, *8*, 137-141.
18. Park, K. S.; Ni, Z.; Côté, A. P.; Choi, J. Y.; Huang, R.; Uribe-Romo, F. J.; Chae H. K.; O'Keefe, M.; Yaghi, O. M. Exceptional chemical and thermal stability of zeolitic imidazolate frameworks. *Proc. Natl. Acad. Sci. U.S.A.* **2006**, *103*, 10186-10191.
19. Morris, W.; Doonan, C. J; Furukawa, H.; Banerjee, R.; Yaghi, O. M. Crystals as Molecules: Postsynthesis Covalent Functionalization of Zeolitic Imidazolate Frameworks. *J. Am. Chem. Soc.* **2008**, *130*, 12626-12627.
20. Sheldrick, G. SHELXT - Integrated space-group and crystal-structure determination. *Acta Crystallogr.* **2015**, *A71*, 3-8.
21. Sheldrick, G. Crystal structure refinement with SHELXL. *Acta Crystallogr.* **2015**, *C71*, 3-8.
22. Rietveld, H. M. Line profiles of neutron powder-diffraction peaks for structure refinement. *Acta Crystallogr.* **1967**, *22*, 151–152.
23. Coelho, A. TOPAS and TOPAS-Academic: an optimization program integrating computer algebra and crystallographic objects written in C++. *J. Appl. Cryst.* **2018**, *51*, 210-218.
